# Metabolomics provides novel understanding of Melissa officinalis mechanism of action ensuring its calming effect on dogs

**DOI:** 10.1186/s12917-025-04904-8

**Published:** 2025-07-11

**Authors:** Anne-Sophie Roy, Fatima Zohra Aberkane, Sekhou Cisse, Aurélie Guibert, Damien Richard, Marie Lerouzic, Sorphon Suor-cherer, Séverine Boisard, David Guilet, Mohammed El Amine Benarbia Benarbia, Mohamed Yassine Mallem

**Affiliations:** 1 Nutrition, PathoPhysiology and Pharmacology (NP3) Unit, 101 Rte de Gachet, Oniris, 44300 Nantes France; 2 Nor-Feed SAS, 3 Rue Amedeo Avogadro, Beaucouzé, 49070 France; 3 Univ Angers, SONAS, SFR QUASAV, Angers, F-49000 France; 4 Labcom FeedInTech, 42 Rue Georges Morel, Beaucouzé, 49070 France; 5 Pharmacology and Biological Toxicology Unit, UMR-INSERM 1107, CHU Clermont Ferrand, 58 rue Montalembert, CLERMONT-FERRAND Cedex 1, 63003 France

**Keywords:** *Melissa officinalis*, Behavior, Metabolomics, Dog’s welfare

## Abstract

**Background:**

Animal welfare encompasses both its physical and mental states. This latter could be altered by several psychological-related disorders including stress and anxiety. To address these issues, *Melissa officinalis*, a *Lamiaceae* plant, ensuring the anxiolytic-type effects, is widely used. In this study, the main aim was to explore the effect of a commercial hydro-alcoholic *Melissa officinalis* extract (MOE) and its major compound rosmarinic acid (RA) on dogs’ behavior and metabolome. To do so, twenty healthy beagle dogs were randomly assigned to 4 dietary supplements (5 dogs/group): the first group received a placebo supplemented with maltodextrose (200 mg/kg), the second group was supplemented with MOE (200 mg/kg), the third group received RA at a dose of 10.6 mg/kg, and the fourth group was administered α-casozepine (AC) at a dose of 225 mg in capsule form. Dogs’ behavior was monitored after 4 weeks of treatment using a standardized evaluation grid developed by Oniris. In addition, 4-hydroxybyturic acid (GHB) was quantified to study the effect of the supplementations on the metabolites of γ-aminobutyric acid (GABA) biosynthetic pathway. Moreover, the impact of all supplementations on dogs’ metabolome was assessed using untargeted metabolomics at the end of the supplementation period.

**Results:**

Results demonstrated significant differences between the mean behavioral score of placebo group (-3.4) compared to MOE (2.0), RA (1.4), and AC (0.8) groups. In addition, GHB measurement revealed a decrease in its quantity in all supplemented groups compared to the control. Moreover, untargeted metabolomics uncovered several metabolic pathways, that were impacted by MOE supplementation linked to lipids and bile acids metabolism. Furthermore, RA supplementation impacted fatty acids and lipids metabolism pathways while supplementation with AC affected pathways linked to lysine and sphingolipids metabolism.

**Conclusions:**

Our study demonstrated a calming effect of MOE on beagles and proposes a novel hypothesis that sheds new light on its potential mechanism of action. This study underlines metabolomics as an effective tool for gaining deep insights into the metabolic changes associated with supplementation.

**Supplementary Information:**

The online version contains supplementary material available at 10.1186/s12917-025-04904-8.

## Background

Stress is a common feature that appears throughout dogs’ life, which can lead to negative impacts on dogs’ welfare and health. In fact, many stressful events can generate several emotional reactions leading to behavioral disorders [1–3]. Therefore, the need for management options that decrease anxiety and stress reactions in dogs is imperative, both for the well-being of the animals and the comfort of the canine caregivers.

Among solutions to manage behavioral disorders, in addition to the behavioral change programs, pharmaceutical agents can be used in dogs [4, 5]. However, their potential contraindications, side and/or adverse effects, and the need for a veterinarian prescription make their use complicated in practice. This has driven researchers towards non-pharmaceuticals agents which are suggested to decrease anxiety and stress reactions in dogs. Feed supplements and/or additives can be effective alternatives to drugs, meeting the needs of consumers concerning the quality and safety of the products they use [6]. Indeed, the perception of dog owners against psychoactive medicine and alternative treatments was evaluated in a recent study and results showed that most of the canine owners felt more comfortable using alternative treatments, compared to drugs [7].

In veterinary behavioral medicine, an integrated approach is commonly adopted, incorporating behavioral modification programs alongside the administration of medications or nutraceuticals [8]. Several nutraceuticals have been studied for their potential calming and anti-stress effects [6, 8–11]. In a recent study, it has been shown by Titeux et al., 2021 that dietary supplements based on fish hydrolysate combined with a concentrate of melon juice resulted in a decrease of associated-stress behavior [6]. Recent evidence also suggests that the use of α-casozepine, a tryptic milk casein hydrolysate, has an anxiolytic-like activity [12, 13]. Various plants and plant extracts can also be used to promote calming behavior in dogs, thanks to the diversity of their bioactive specialized metabolites that guaranty the calming properties [14–16]. Among plants, lemon balm (*Melissa officinalis*), belonging to the *Lamiaceae* family, has been used since antiquity for its soothing properties [17–19]. *Melissa officinalis* contains a large amount of rosmarinic acid (RA), which has been reported to exhibit anxiolytic properties [20]. The leaf of lemon balm also contains flavonoids (quercitrin, rhamnocitrin, luteolin) and phenolic acids (RA, caffeic acid, and protocatechuic acid) [21] which have therapeutic potential in the management of behavioral disorders. Calming and anxiolytic effects of *Melissa officinalis* are widely documented in the literature, especially in humans [22–24]. However, to the best of our knowledge, few or no studies have been performed on the anxiolytic effect of *Melissa officinalis* and its mechanism of action in dogs. Moreover, it remains unclear whether the potential beneficial effect of *Melissa officinalis* involves synergistic interaction or multifactorial effects between RA and other ingredients present in lemon balm.

Therefore, this study aimed to evaluate the effect of a hydroalcoholic *Melissa officinalis* leaf extract (MOE) long-term dietary supplementation on the behavior of healthy beagle dogs exposed to mild stress. MOE effect on mild stress was compared to RA, considered as the major active substance of MOE. We hypothesized that MOE supplementation’s effect on dogs’ behavior would produce a greater beneficial effect than that produced by RA, at a dose being the level of RA present in the MOE. Characterization was also performed on MOE to better understand the relationship between composition and mode of action. The impact of MOE and RA supplementation was also investigated on dogs’ metabolome using untargeted metabolomics, as it is considered as a powerful tool to provide new insight into their mechanism of action by elucidating the impacted behavior-related pathways.

## Methods

### Animals

Sixteen healthy males and four healthy females beagles (Isoquimen, SL, Spain) aged 2.7 ± 0.1 years and each weighing between 9 and 16 kg were used in this study. Dogs with an unsuitable temperament (as evaluated by the manager of the kennel) were excluded. Prior to the study, dogs were already familiar with each other and were housed in groups of three to five. Dogs had daily access to a fenced park with trees for at least 40 min, allowing exercise and social interaction. Additionally, each dog was taken for a weekly 30-minute walk on a leash. The study included two females as well as three male beagles that were not neutered. In each enclosure of the kennel, three to four dogs were housed according to their affinities with free access to water in a large outdoor/indoor kennel that was cleaned once a day. A walk for 45 min in a park was organized once a day. The dogs were weighed once a week and observed at least twice daily for any physical abnormalities or clinical signs.

### Diet and protocol

Each dog was fed once a day with the same commercial dry food (Ultima-affinity^®^) for one month. Pet food fulfilled the recommendations for protein, carbohydrate and fat regarding dog daily energy needs (Nutritional Guidelines for Complete and Complementary Pet Food for Cats and Dogs—The European Pet Food Industry Federation). Briefly, the nutrient composition was 25% crude protein, 16% crude fat, 2.75% crude fiber, and 7% crude ash, with an energy value of 3855 (kcal/kg). The dog’s ration was calculated according to their energy requirements. The dogs were randomly divided into 4 groups (5 dogs per group). The first group, composed by five males including 2 un-neutered, was supplemented with the standard *Melissa officinalis* extract (MOE) at 200 mg/kg of feed. The second group, composed by 4 males and 1 female, was supplemented with placebo composed by maltodextrose, at the same concentration as MOE. The third group, composed by 3 males and 2 un-neutered females, was supplemented with rosmarinic acid (RA) in powder form (Sigma-Aldrich, Saint Louis, USA) in food at 10.6 mg/kg of feed which was the quantity found in the dose of 200 mg/kg of MOE. The last group, composed by 4 males and 1 female, was daily supplemented with 225 mg of a dietary complement named Zylkene^®^ composed by α-casozepine (AC) with anxiolytic properties [25, 26]. This group served as reference. The different supplements were administered once a day, in the morning, during meals, and were placed directly in the kibble rations by animal technicians. The duration of supplementation was 4 weeks.

### Behavioral observation

A grid of the dog’s behavior was produced according to various evaluation criteria to assess the dog’s behavior in the kennel and under walking conditions (Table 1). The behavioral tests were based on literature procedures used to evaluate behavioral reactions in dogs faced with a variety of mild stress situations [27, 28]. The evaluation of the behavioral responses of dogs in the kennel allowed us to ensure their good health and that their behavior during the tests was only a reflection of their personality. The various tests carried out make it possible to analyze the behavior of dogs faced with an unusual situation. Each item is scored from 0 to 4. The highest scores corresponded to the normal behavior of the animal. The tests by this grid were carried out double-blind the week before supplementation and then at 4 weeks after the supplementation.

**Table 1:** Evaluation grid of dog’s behavior in the kennel environment and in walking. The total score for the kennel part corresponded to a score of 40, and for the walk part a score of 24. The total score for both parts corresponded to a score of 64

	Domain	Item	Rating criteria				
			0	1	2	3	4
**At the kennel**	**Physical domain**	**Underweight**	anorexia, dehydrated	Weight loss between > 5 kg	Weight loss between > 3 kg	Weight loss between > 1 kg	Normal
		**Overweight**	Severe overweight	Weight gain > 5 kg	Weight gain > 3 kg	Weight gain > 1 kg	Normal
		**Locomotion**	Can’t move alone	Moves with difficulty, less than 10 steps	Moves slowly, less than 20 steps	Less than 30 steps	Mobile, more than 40 steps
	**Psychological domain**	**Temperament**,** mood**	Aggressive	Menacing, growling	Sad look	Less cuddly	Normal, unchanged
		**Activity (motivation**,** curiosity)**	Downcast, lethargic	Stay in their corner, don’t come when called	Doesn’t play, come when called	unmotivated to play	Normal (play, explore)
	**Field of social relations**	**Interaction with animal technician (the animal technician entered each pen and waited for the dogs to come to them.)**	No interaction, stay in your corner	Lack of enthusiasm, stay seated	No interaction, doesn’t jump but wag tail	Less interaction than usual	Normal, unchanged (jump on animal technician, enthusiastic tail wagging)
		**Interaction with other dogs**	Fights, bites, threats with any individual including the appearance of vocalizations (e.g. barking)	A few fights, targeted threats (gender, height…)	Punctual aggressions (threats)	No physical aggression but growling or barking	Normal (no growling, no biting or indifferent to other dogs)
	**Behavior domain**	**Unfamiliar person test**	Bad interaction, the dog is aggressive	Growling, some barking	Fearful (away from abroad), some grunts	Indifferent to an unfamiliar person (don’t look at him, don’t approach or go away from the unfamiliar person)	Good interaction, the dog approaches a stranger, no growling or barking
		**Stranger noise test (bicycle horn)**	Aggressive (growling, barking)	Growling, some barking, fear of noise	Fearful (moves away from noise), some growling	Indifferent to unknown noise, does not react	Curious, no growling or barking
		**Unfamiliar object test (a yellow trash can)**	Aggressive (growling, barking, biting the object)	Growling, some barking, fear of object	Fearful (moves away from object), some growling	Indifferent to unknown object (do not look at the object)	Curious (plays with the object, sniffs it), no growling or barking
							**Total at the kennel /40**
**Walking**	**Behavior domain**	**Unfamiliar person test**	Bad interaction, the dog is aggressive	Growling, some barking	Fearful (get away from abroad), some grunts	Indifferent to an unfamiliar person (don’t look at him, don’t approach or go away from the unfamiliar person)	Good interaction, the dog approaches the stranger, no growling or barking
		**Unfamiliar animals test (cows**,** horses)**	Aggressive (growling, barking)	Growling, some barking, fear of the animal	Fearful (get away from the animal), some grunts	Indifferent to an unknown animal (don’t look at it)	Curious about the animal
		**Stranger noise test (bicycle horn)**	Aggressive (growling, barking)	Growling, some barking, fear of noise	Fearful (moves away from noise), some growling	Indifferent to unknown noise, does not react	Curious, no growling or barking
		**Unfamiliar object test (remote controlled car)**	Aggressive (growling, barking, bite the object)	Growling, some barking, fear of object	Fearful (moves away from object), some growling	Indifferent to unknown object (do not look at the object)	Curious (plays with the object, sniffs it), no growling or barking
	**Physical domain**	**Physical exercise in a park**	No play, stay in a corner, lying down	Walk in the park, but don’t jump or run	Does not play with other dogs but runs in the park	Play, run and jump less than usual	Normal, the dog runs, jumps, plays with other dogs
		**Exercise tolerance**	Intolerant (inability to provide and sustain physical effort)	Very little tolerant (very tired from the walk, shows no motivation)	Not very tolerant (walks slowly, fatigue)	Walks slower than usual but good endurance	Optimal (shows enthusiasm for walking, good stamina)
							**Total in walking /24**
							**Total /64**

#### Interaction with an unfamiliar person

The test was carried out with different people at each assessment to avoid habituation effects. The unfamiliar person approached the kennel from the outside and stood in front of each box. Throughout the duration of the observation, the unfamiliar person maintained a stationary and immobile stance. No eye contact was made with the dogs. The two observers stood next to an unfamiliar person and assessed the behavior of the dogs.

#### Loud noise test

A bicycle horn noise was emitted in front of each enclosure by one of the observers for 20 s. On walks, the sound was emitted at a given time and in a different place each time without anyone around so as not to disturb the dogs during the evaluation.

#### Response to an unfamiliar object

*A* yellow trash can was the object chosen for the test. The dogs had never seen this object before the protocol. They were placed inside the enclosure and the two observers evaluated the behavior of the dogs in front of the object.

#### Unfamiliar animals test

During the walk, the dogs encountered unfamiliar animals such as horses or cows but also dog owners. All the assessed dogs were secured on a leash, and the two observers assessed their behavior in front of one of these animals.

### Characterization of MOE hydroxycinnamic acids

#### Solvents, analytical standards, and sample preparation of MOE

MOE as weighed (50 mg) and solubilized in 1 mL of methanol. After 5 min of sonication bath (S30H, Elmasonic, Germany), the mixture was centrifuged for 10 min at 13,000 g and the supernatant was recovered for High-Performance Liquid Chromatography coupled with mass spectrometry (HPLC-MS) analysis and High-Performance Liquid Chromatography-Ultra-Violet (HPLC-UV) analysis. All solvents used in High-Performance Liquid Chromatography (HPLC) were of HPLC grade. Methanol and acetonitrile were purchased from Sigma-Aldrich. Deionized water from a Millipore Milli-Q water system was used to prepare mobile chromatographic phases. To confirm MOE active compounds identification, analytical standards (chlorogenic acid, caffeic acid, p-coumaric acid, ferulic acid and rosmarinic acid) were purchased from Extrasynthese (Genay, France).

#### High-performances liquid chromatography analysis

HPLC-UV analysis (Waters Separations Module 2695 equipped with a dual-wavelength UV/Visible Detector 2489 (Waters, Manchester, UK)) was performed following the methodology employed by Dastmalchi et al., 2008 and using a Zorbax SB-C18 (250 mm x 4.6 mm, 5 μm) analytical column (Agilent, Les Ulis, France) [21]. The objective here was to determine the HPLC-UV profile of MOE hydroxycinnamic acids. Each analysis was performed on 5 different batches of MOE. The injection volume was 10 µL. The mobile phase was composed of 0.1% of formic acid in deionized water (A) and 0.1% of formic acid in acetonitrile (B) at a flow rate of 1 mL/min. The initial condition was 90%(A), 10%(B). A gradient program was performed as follows: (0–20 min) 10–35% B; (20–23 min) 35% B; (23–26 min) 35–100% B; (26–27 min) 100% B; (27–28 min) 10% B. The identification of MOE active compounds was confirmed by comparing their retention times to those of authentic standards under identical analysis conditions and by comparison of their UV spectra with the profile of each MOE peak.

#### Quantification of MOE hydroxycinnamic acids

Different concentrations of rosmarinic acid (0.5, 0.25, 0.12, 0.06, 0.03, and 0.0125 mg/mL) were prepared and analyzed following the same methodology as described in HPLC-UV part. The area under the curve was monitored for each concentration and the calibration curve was done by plotting the absorbance (A280nm) as a function of the area under the curve. Then, a 60 mg quantity of MOE was prepared and analyzed following the same methodology as described in the HPLC-UV part. The area under the curve was monitored for each active compound identified in MOE. Then, the compounds were quantified using the calibration curve prepared previously. The concentration of each compound was determined following the formula below:

$$\:C=\frac{\text{a}\text{r}\text{e}\text{a}\:\text{u}\text{n}\text{d}\text{e}\text{r}\:\text{c}\text{u}\text{r}\text{v}\text{e}-\text{b}}{a}$$

With:

b = intercept of the calibration curve.

a = slope of the calibration curve.

Results are expressed in rosmarinic acid equivalent.

### Sample collection, biochemical analysis and hematology

To assess the safety of the supplementations for the dogs, jugular vein blood samples were collected from each dog at week 0 (W0) and at week 4 (W4) of supplementation to perform biochemical and hematology analysis. For biochemical analysis, heparinized plasma samples were centrifuged at 2100 g for 10 min at 4 °C and stored at -20 °C up to until analysis for the levels of alanine aminotransferase (ALT), aspartate aminotransferase (AST), alkaline phosphatase (ALP), urea and creatinine. The analysis was performed following the manufacturer’s guidelines of the automated analyzer RX Daytona + purchased from Randox laboratories Ltd. EDTA blood samples were collected for hematology analysis: leukocytes (Leu), red blood cell (RBC) count, hemoglobin (Hb), hematocrit (HCT), mean corpuscular volume (MCV), mean corpuscular hemoglobin (MHC), mean corpuscular hemoglobin concentration (MCHC), red cell distribution index (RDI), platelets (PLT), neutrophils (Neu), lymphocytes (Lymphs), monocytes (Mono), eosinophils (Eos). The hematology analysis was performed using a Sysmex XN-1000 V automated analyzer (Sysmex Corporation, Kobe, Japan) according to the manufacturer’s instructions.

### γ-Hydroxybutyric acid (GHB) level in plasma

#### Stock solution and sample preparation

Stock solutions of GHB and GHB-d6 (internal standard, IS) were prepared at 1 g/L and 0.1 g/L in methanol respectively. All standard solutions were stored at -20 °C. Working solutions of GHB were prepared at 10 mg/L and 1 mg/L in methanol and IS was at 10 mg/L in methanol.

Six calibration curve points were constructed in the concentration range of 50–1000 µg/L as well as different quality controls (25, 150, 500 and 2000 µg/L). The analyzed samples were prepared by spiking 50 µL of IS (1 mg/L) into 1000 µL of biological sample. Sample solutions (standards, controls and samples) were extracted using 5 mL of ethyl acetate. After mixing and centrifuging, the upper organic layer was collected and evaporated under a stream of nitrogen at room temperature. The extract was reconstituted in 75 µL acetonitrile and 25 µL N-tert-butyldimethylsilyl-N-methyltrifluoroacetamide (MTBSTFA). The mixture was vortexed for 30 s. The derivatization step was carried out at 60 °C for 30 min. The cooled final solution was transferred into a crimp top vial with insert and loaded onto the GC-MS auto-sampler.

#### Gas chromatography and mass spectrometry

The analysis was performed on a HP5973 MS with a HP6890 series GC (Agilent Technologies, Atlanta, GA, USA). Automatic injections were made using a HP6890 autosampler. The temperature of the injector and the transfer line detector were 180 °C and 280 °C, respectively. The glass liner was equipped with a Siltek deactivated inlet glass liner (Restek, Bellefonte, PA). The GC was operated in the splitless injection mode with a constant flow of 1.7 mL/min helium through the HP-5MS column (30 m x 0.25 mm ID with 0.25-ím film thickness (J&W, Folsom, CA)). The oven temperature GC was programmed starting from 110 °C and increased to 280 °C at 9.5 °C/min. GHB-d6-TMS eluted at 8.26 min and GHB-TMS eluted at 8.22 min. Ions were detected by selective ion monitoring (SIM). An HP Chemstation^®^ software system controlled the equipment and carried out the data processing.

Three and two multiple reactions monitoring (MRM) transitions (GHB: m/z 275, 201, 185 and GHB-d6: m/z 281 and 323) were monitored for GHB-TMS and GHB-d6-TMS, respectively.

The sensitivity of the method was determined to have a limit of quantitation (LOQ) of 15 µg/L and a limit of detection of (LOD) 5 µg/L. The result limit is assigned to the lowest control level, i.e. 25 µg/L.

### Metabolomic analysis

#### Metabolite extraction

Plasma samples collected after 4 weeks of supplementation were analyzed. Metabolites were extracted according to Zhang et al., 2019 with some modifications [29]. Briefly, 100µL of plasma was mixed with 2 µL of each of proteinase K (20 mg/mL) and CaCl_2_ solution (250 mmol/L). The mixture was then incubated in a water bath at 37 °C for 15 min. Then, for protein precipitation, 300 µL of chloroform/methanol (3/1:v/v) was added, vortexed for 3 min and incubated overnight at -20 °C. The mixture was centrifuged, and the supernatant was collected, evaporated using a Genevac TM EZ-2 evaporator, and resuspended in dichloromethane/methanol (1/1:v/v) (hydrophobic extracts, HPO). The pellet was used for a second extraction step using 300 µL of methanol/water (3/1:v/v). The mixture was centrifuged, and the supernatant was collected to be evaporated and resuspended in acetonitrile/water (1/1:v/v ) (hydrophilic extracts, HPI).

#### UPLC-QTOF-HRMS analysis and data processing

The extracts were analyzed using an ultra-high performance liquid chromatography (UPLC) ACQUITY H-Class PLUS (Waters, Manchester, UK) coupled to a quadrupole time-of-flight-high resolution mass spectrometry (QTOF-HRMS) Xevo G2-XS (Waters, Manchester, UK). Chromatographic separation was performed using a BEH C18 column 2.1 × 150 mm with 1.7 μm particle size (Waters). The column and autosampler temperatures were maintained at 25 °C and 15 °C, respectively. The mobile phase A consisted of water with 0.1% (v/v) formic acid and the mobile phase B was composed of acetonitrile with 0.1% (v/v) formic acid. The elution gradient was applied as the following: (0–1 min) 2% B; (1–9 min) 2–35% B; (9–17 min) 35–100% B with an elution rate of 0.4mL/min. A sequence was constructed to minimize the alterations in signal detection. A mix of each of the hydrophobic and the hydrophilic extracts was prepared separately for quality control (QC) samples. Electrospray ionization (ESI) was used in both positive and negative ionization. Mass spectrometry conditions for both ionization modes were set as the following: capillary voltage: 0.5 kV; sampling cone: 40 kV; source offset: 80; source: 120 °C; desolvation: 500 °C; cone gas: 100 L/h; desolvation gas: 1000 L/h. Data was recorded using MS^E^ mode. Progenesis QI (Waters, Manchester, UK) was used for data processing, identification, relative quantification and statistical analysis.

#### Metabolites identification and pathway analysis

The metabolites lists were based on the variable importance of projection (VIP) > 1 and *p*-value < 0.05. The identifications were accepted based on the following criteria: score > 35, mass error (ppm) < 5, isotope similarity > 70. To our knowledge, studies using metabolic pathway databases of dogs are scarce. Furthermore, several metabolome characteristics of dogs showed high similarity to those in human studies, suggesting shared biochemical mechanisms between dogs and humans [30, 31]. Therefore, the Human Metabolome database (HMDB) was exported from (https://hmdb.ca/) and from MassBank of north America (MoNA) (https://mona.fiehnlab.ucdavis.edu/) since this latter contains files compatible with progenesis QI (.sdf). Databases were imported to Progenesis^®^ QI and used for metabolite identification. Integrated Molecular Pathway Level Analysis (IMPaLA, (http://impala.molgen.mpg.de/) was used for pathway analysis. IMPaLA permits to search the input metabolites in several pathway databases. Results are presented in a table containing the name of the database in which the pathway is found (pathway source); the number of the entered metabolites which are found in that pathway (overlapping metabolites), the number of all the metabolites of the pathway (all metabolites) and *p*-value calculated based on the number of metabolites overlapped on all the metabolites (Pmetabolite). Values of *p*-value < 0.05 were considered statistically significant [32].

### Statistical analysis

For behavioral observations with the evaluation grid, Shapiro-Wilk test was used to assess the normality of data. The data were expressed as mean values ± SEM. One-way analysis of variance (ANOVA) was performed followed by Tukey’s post hoc test for multiple comparisons. All statistical analyses were performed using GraphPad Prism software version 9. For metabolomics, permutation tests to validate orthogonal partial least squares discriminant analysis (OPLS-DA) were performed using Ropls package in R studio [33]. ANOVA tests were performed on Progenesis^®^ QI and values of *p* < 0.05 were considered statistically significant.

## Results

### Evaluation of the behavior of the dogs using a grid after 4 weeks of supplementation

**Fig. 1:**
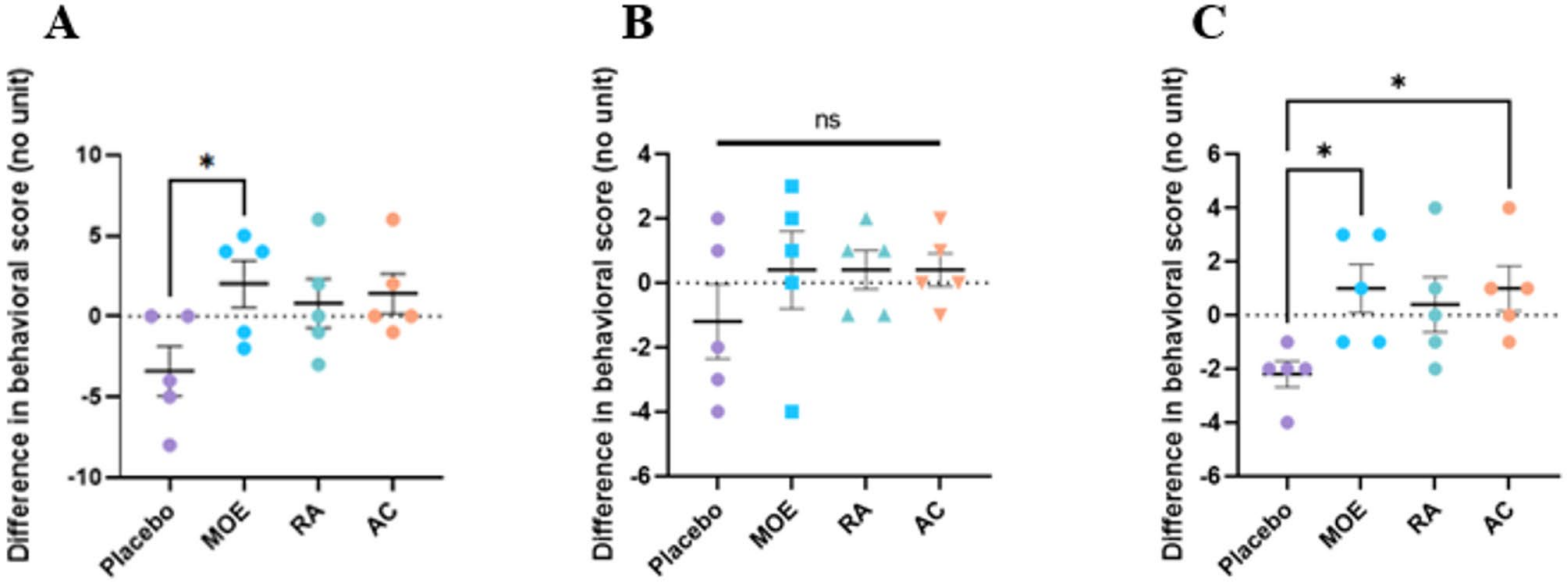
Changes in (**A**) overall behavior of healthy beagle dogs, in (**B**) the kennel and (**C**) during walking before and after 4 weeks of supplementation with MOE, RA or AC. The scores of each dog in each group were added and then averaged, the means of the scores of week 4 were subtracted from those of week 0. The graph represents the difference in total behavior score between week 0 and week 4 (*N* = 20, *n* = 5; *: *p* < 0.05; ns: not significant)

The behavior of the beagles fed with various supplements were assessed using an evaluation grid. The higher the score, the greater the effect. A score from 0 to 4 was used to rate the behavior in the kennel and during the walks (Table 1). Different items were evaluated in relation to the animal’s quality of life (hydration, appetite, activity, etc.) and the dog’s behavior facing stress stimuli (unknown object, person, noise and animal). Scores were added for each group and then averaged [28]. Figure 1 represents the graph corresponding to the difference in mean between week 4 (4 weeks after supplementation) and week 0 (week before supplementation).

After 4 weeks of supplementation, a difference between the overall mean scores of the placebo group and that of those three experimental groups was observed (Fig. 1A). Indeed, the dogs in the placebo group (maltodextrose) had a negative average score (-3.4), indicating that the behavior of the dogs at week 4 deteriorated compared to the week before supplementation in the face of different stress stimuli (Fig. 1A). However, this did not correspond to a deterioration in its physical and psychological state since the mean scores for these items did not change (Table S1). In contrast, a positive average score indicated an improvement in behavior facing stressful stimuli. Indeed, an increase in the experimental groups RA (1.4) and AC (0.8) after 4 weeks of supplementation with a significant difference in the MOE group (2.0) compared to the placebo group (-3.4) was observed (Fig. 1A).

Regarding the difference between dog’s behavior in the kennel and on walks (Fig. 1B and C), results showed an improvement in the MOE, RA, and AC groups compared to the placebo group on walks. Dogs from these groups were more curious and calmer in the face of different stress stimuli compared to dogs from the placebo group which had a rather apprehensive and fearful behavior. Interestingly, only the groups that received MOE and AC supplementation showed a significant difference compared to the placebo group. On the other hand, when the dogs were in the kennel, no significant difference in score was measured (Fig. 1B).

### Characterization of MOE hydroxycinnamic acids

**Fig. 2:**
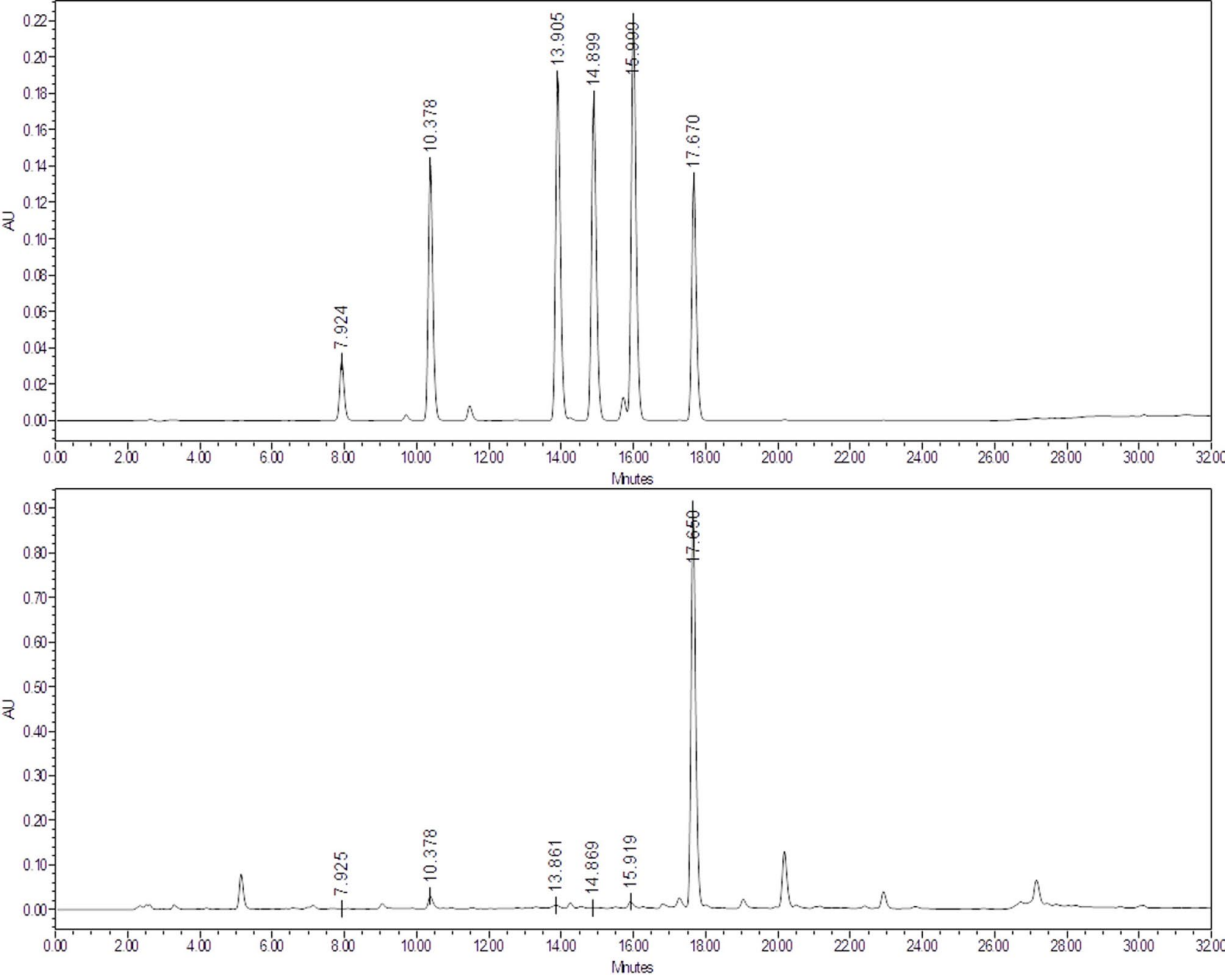
Chromatogram of mixed analytical standards of hydroxycinnamic acids (**A**) and chromatogram of MOE extract recorded in same conditions (**B**)

MOE hydroxycinnamic acid characterization was performed using HPLC-UV method. The HPLC-UV profile of MOE hydroxycinnamic acid (Fig. 2) allowed to identify 6 compounds (Table 2).

**Table 2:** MOE hydroxycinnamic analysis revealing 6 identified components

Peak	Hydroxycinnamic acid derivatives	Rt (min)	Quantity (mg/g)	% in MOE
1	Chlorogenic acid	7.9	0.79	0.08
2	Caffeic acid	10.4	0.68	0.07
3	*p*-Coumaric acid	13.9	< 0.1	< 0.01
4	Ferulic acid	14.8	< 0.1	< 0.01
5	*m*-Coumaric acid	15.8	< 0.1	< 0.01
6	Rosmarinic acid	17.6	54	5.4

Rosmarinic acid has been identified as MOE major hydroxycinnamic compound (54 mg/g), followed by chlorogenic acid (0.79 mg/g), and caffeic acid (0.68 mg/g).

### Analysis of hepatic and renal markers and complete blood count of dogs in the placebo, MOE, RA and AC groups

**Fig. 3:**
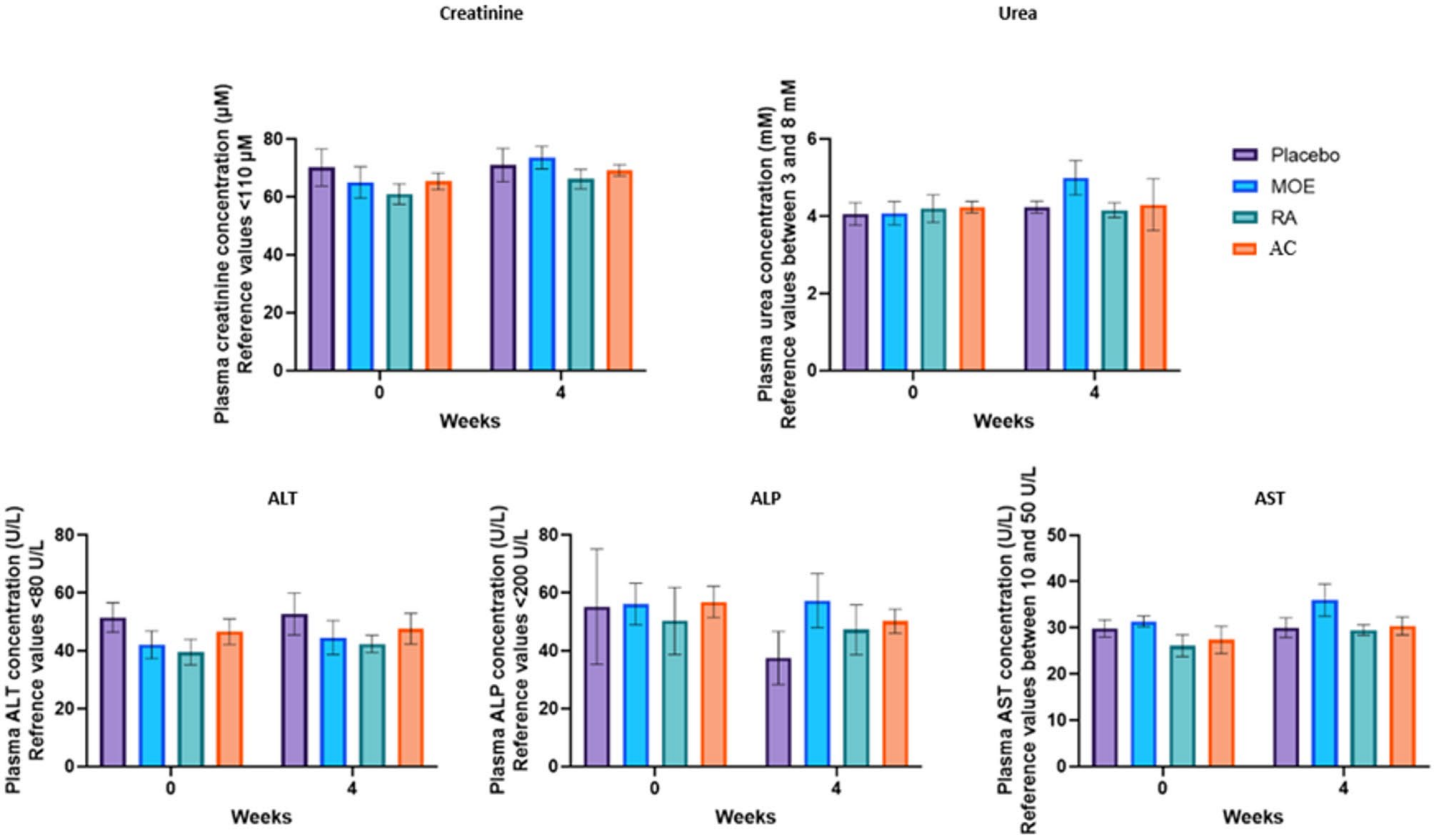
Plasma metabolites levels (Creatinine, Urea, ALT, ALP, and AST) on dogs before and after 4 weeks of supplementation with placebo, *Melissa officinalis* extract (MOE), rosmarinic acid (RA) and α-casozepine (AC). The dosage of the different markers was measured in the plasma of the dogs in each group at 0 (before supplementation) and 4 weeks after supplementation (*N* = 20, *n* = 5 per group, ns: not significant)

Blood samples were collected before and after 4 weeks of supplementation to ensure the safety of treatments administered to dogs. The levels of hepatic (ALT, ALP, and AST), renal biomarkers (creatinine and urea) and complete blood count were measured and are presented in Fig. 3; Table 3, respectively.

**Table 3:** Mean values obtained for each variable at 0 and 4 weeks after placebo, MOE, RA and AC supplementation in dogs. The levels of the different variables were assayed in the plasma of the dogs in each group at 0 and 4 weeks after supplementation. (*N* = 20, *n* = 5). Leukocytes (Leu), red blood cell (RBC) count, hemoglobin (Hb), hematocrit (HCT), mean corpuscular volume (MCV), mean corpuscular hemoglobin (MHC), mean corpuscular hemoglobin concentration (MCHC), red cell distribution index (RDI), platelets (PLT), neutrophils (Neu), lymphocytes (Lymphs), monocytes (Mono), eosinophils (Eos)

	Groups	Placebo		MOE		RA		AC	
Variable	Unit / Weeks	0	4	0	4	0	4	0	4
**Leu**	**10** ^**3**^ **/µL**	8.83 ± 0.58	10.50 ± 1.24	7.39 ± 0.63	8.43 ± 0.81	7.75 ± 0.67	9.32 ± 1.32	7.32 ± 0.20	8.30 ± 0.51
**RBC**	**10** ^**6**^ **/µL**	7.23 ± 0.22	6.92 ± 0.14	6.93 ± 0.19	6.95 ± 0.14	7.06 ± 0.25	6.86 ± 0.31	7.03 ± 0.34	6.87 ± 0.15
**Hb**	**g/dL**	16.42 ± 0.41	15.88 ± 0.39	15.52 ± 0.52	15.77 ± 0.60	16.40 ± 0.56	22.08 ± 0.26	16.12 ± 0.60	16.12 ± 0.39
**HCT**	**%**	48.62 ± 1.28	47.40 ± 1.23	47.10 ± 1.69	47.06 ± 1.52	49.40 ± 1.56	47.50 ± 1.85	48.30 ± 1.84	47.02 ± 1.24
**MCV**	**fL**	67.36 ± 1.46	68.54 ± 0.76	67.94 ± 0.85	67.16 ± 0.84	69.98 ± 0.41	69.32 ± 0.53	68.88 ± 0.90	68.46 ± 1.20
**MHC**	**%**	33.76 ± 0.79	33.52 ± 0.51	32.94 ± 0.66	33.96 ± 0.48	33.18 ± 0.88	33.86 ± 0.48	33.40 ± 0.34	33.88 ± 0.49
**MCHC**	**pg**	22.72 ± 0.64	22.94 ± 0.51	22.40 ± 0.58	22.82 ± 0.80	23.26 ± 0.65	23.46 ± 0.44	22.98 ± 0.74	23.18 ± 0.67
**RDI**	**%**	14.98 ± 1.36	15.52 ± 0.61	14.64 ± 0.96	15.70 ± 0.72	14.34 ± 1.23	14.76 ± 1.59	15.24 ± 1.77	15.02 ± 1.10
**PLT**	**10** ^**6**^ **/µL**	270.40 ± 34.3	298.00 ± 45.2	304.40 ± 65.2	366.00 ± 41.6	328.20 ± 47.9	341.40 ± 33.2	328.00 ± 23.6	371.60 ± 44.4
**Neu**	**10** ^**3**^ **/µL**	5.55 ± 0.66	7.21 ± 1.20	4.71 ± 0.36	5.29 ± 0.66	5.21 ± 0.45	5.91 ± 1.14	4.09 ± 0.23	5.02 ± 0.62
**Lymph**	**10** ^**3**^ **/µL**	2.23 ± 0.18	2.02 ± 0.40	2.18 ± 0.35	2.27 ± 0.32	1.77 ± 0.27	2.51 ± 0.26	2.43 ± 0.47	2.49 ± 0.45
**Mono**	**10** ^**3**^ **/µL**	0.42 ± 0.03	0.57 ± 0.16	0.29 ± 0.07	0.38 ± 0.09	0.44 ± 0.05	0.44 ± 0.09	0.47 ± 0.06	0.26 ± 0.05
**Eos**	**10** ^**3**^ **/µL**	0.36 ± 0.12	0.60 ± 0.10	0.19 ± 0.06	0.45 ± 0.11	0.26 ± 0.09	0.38 ± 0.13	0.30 ± 0.08	0.44 ± 0.07

As shown in Fig. 3, no significant differences between the four groups in plasma creatinine, urea, ALT, ALP, and AST levels after the 4 weeks of supplementation were observed. The values ​​obtained for the levels of the markers measured were similar to the reference values ​​in dogs.

No significant changes were observed among the four groups for the observed different blood parameters in dogs after 4 weeks of supplementation (Table 3).

### Plasma GHB level of each control (placebo) and experimental groups (MOE, RA, and AC)

**Fig. 4:**
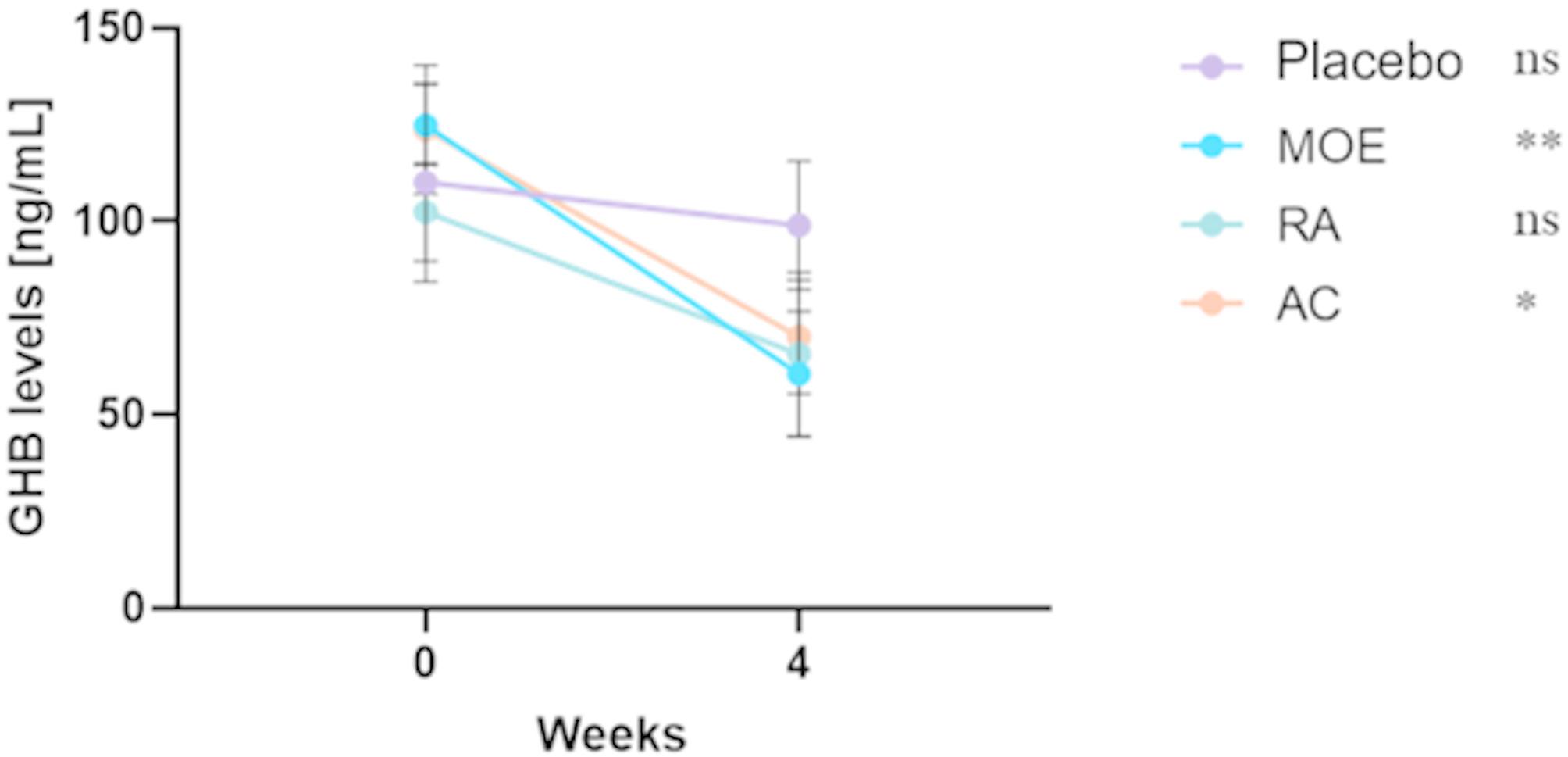
Evolution of the GHB level in plasma of dogs supplemented with placebo, MOE, RA and AC after 4 weeks of supplementation. The plasma GHB level for each group was performed before and after 4 weeks of supplementation and a significant decrease of plasma GBH level was observed for MOE and AC groups (*N* = 20, *n* = 5; **: *p* < 0.01; *: *p* < 0.05; ns: not significant)

To better understand the behavior of dogs supplemented with placebo, MOE, RA and AC, the dosage of GHB, a precursor of GABA, the main “inhibitory” neurotransmitter of the central nervous system, was carried out in plasma before and after 4 weeks of supplementation (Fig. 4).

Results revealed that GHB levels in plasma of placebo group slightly decreased after 4 weeks of supplementation. Surprisingly, a significant decrease in plasma GHB level was observed in the three experimental groups during the same period. Indeed, a decrease of 52%, 44%, and 36% was observed for MOE, AC and RA groups respectively, compared to the week before supplementation (Fig. 4).

### Metabolomic analysis

#### Plasma canine multivariate data analysis

The extraction method based on a two-step approach showed a broad metabolite coverage. The HPO analysis revealed 22,697 and 32,551 features in ESI^−^ and ESI^+^ modes, respectively. Considering these findings, only the ESI^+^ mode was used to analyze the hydrophilic extracts (HPI), revealing 19,658 detected features.

**Fig. 5:**
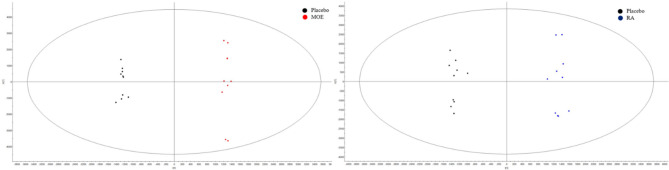
Metabolomic profiles of beagle dogs’ plasma generated by OPLS-DA of HPO extracts in ESI^−^ mode. Placebo and MOE comparison; placebo and RA comparison

Next, to investigate these datasets, orthogonal partial least squares-discriminant analysis (OPLS-DA) was used to compare placebo to each of MOE, RA and AC groups. Permutation tests were used to evaluate OPLS-DA models. Results indicated that the models constructed from HPO extracts in ESI^−^ mode were not overfitted when comparing the placebo group to the MOE group (R2Y = 0.996, Q2Y = 0.883), nor when comparing the placebo group to the RA group (R2Y = 0.993, Q2Y = 0.887) (Fig. 5). Surprisingly, when comparing the placebo group to the AC group, no valid OPLS-DA model could be constructed as the first predictive component was not statistically significant, indicating insufficient discrimination between these groups based on their metabolomic profiles. However, for all the other comparisons (HPO and HPI in ESI^+^), permutation tests validated the OPLS-DA models (Table S2). Moreover, for all the analyses, except the comparison between placebo and AC of HPO extract in ESI^−^, the original models outperformed most of the permuted tests (Fig. S1). These findings indicate that supplementing dogs with either MOE, RA or AC, resulted in alterations to their metabolic profiles.

#### Metabolites identification

Based on the identification criteria, placebo and MOE groups comparison of HPO extracts in ESI^−^ mode revealed 1069 differently expressed features (*p*-value < 0.05 and VIP > 1), of which 607 features were putatively identified and 174 metabolites whose identifiers (ID) were accepted (Table S3). Similarly, placebo and RA groups comparison revealed 982 differently expressed features. Among them, 460 were putatively identified but only 190 identifications were accepted (Table S4).

Regarding HPO extracts in ESI^+^ mode, 2112 features were differently expressed, 640 were identified but only 192 identifications were accepted when comparing placebo to MOE group (Table S5). Moreover, comparison between placebo and RA revealed 1945 differently expressed features, 542 of them were identified and 186 were accepted (Table S6). Furthermore, 814 features were differently expressed between placebo and AC groups. Databases investigation led to the putative identification of 200, of which 63 were accepted (Table S7). As for HPI extracts in ESI^+^ mode, comparison of placebo and MOE groups revealed 1394 differently expressed features (*p*-value < 0.05 and VIP > 1). Of them, 126 identifications were accepted from a total of 536 putatively identified features (Table S8). Regarding placebo and RA groups comparison, the analysis demonstrated 307 differently expressed features. Out of 161 putatively identified features, 72 identifications were accepted (Table S9). Finally, the comparison between Placebo and AC groups uncovered 1949 features with differential expression. The identification approach revealed 681 putatively identifiers of which 197 were accepted (Table S10).

#### Metabolic pathway analysis

To further explore MOE and RA potential mechanism of action, the identified metabolites were employed to perform a pathway analysis using the web tool IMPaLA.

**Table 4:** Metabolic pathways potentially regulated by MOE of HPO extracts in ESI^−^ mode

Pathway name	Pathway source	Overlapping metabolites	All metabolites	Pmetabolites
Bile acid biosynthesis	SMPDB	5	58	1.65e-05
Metabolism of lipids	Reactome	9	352	6.4e-05
Glycosphingolipid biosynthesis	EHMN	3	18	0.000146
Bile secretion	KEGG	5	112	0.000398
Cholesterol metabolism	KEGG	2	7	0.000727
Ganglio sphingolipid metabolism	Wikipathways	2	8	0.000966
Metabolism of spingolipids in ER and Golgi apparatus	Wikipathways	2	10	0.00154
Transport of organic anions	Reactome	2	13	0.00264
Digestion of dietary lipid	Reactome	2	14	0.00307
Sphingolipid metabolism	Reactome	3	54	0.00388
Heme degradation	Reactome	2	18	0.00509
Drug induction of bile acid pathway	Wikipathways	2	18	0.00509
Recycling of bile acids and salts	Reactome	2	20	0.00628
Linoleate metabolism	EHMN	2	21	0.00691
Glucuronidation	Reactome	2	28	0.0121
Metabolism of porphyrins	Reactome	2	34	0.0176

The analyses of the three datasets suggested that MOE regulated several pathways including bile acid biosynthesis, glycosphingolipid biosynthesis, amino acids metabolism, and overall lipid and cholesterol metabolisms (Table 4, Table S11, Table S14).

**Table 5:** Metabolic pathways potentially impacted by RA of HPO extracts in ESI^−^ mode

Pathway name	pathway source		overlapping metabolites	all metabolites	Pmetabolites
Glycosphingolipid biosynthesis		KEGG	2	7 (31)	0.000171
Ganglio sphingolipid metabolism		Wikipathways	2	8 (19)	0.000228
Metabolism of spingolipids in ER and Golgi apparatus		Wikipathways	2	10 (21)	0.000366
Glycosphingolipid biosynthesis - ganglioseries		EHMN	2	18 (38)	0.00123
Glycosphingolipid metabolism		Reactome	2	31 (38)	0.00366
Phase II - conjugation of compounds		Reactome	3	115 (149)	0.00397
Cytosolic sulfonation of small molecules		Reactome	2	35 (47)	0.00465
Sphingolipid metabolism		Reactome	2	54 (65)	0.0109
Biological oxidations		Reactome	3	231 (320)	0.0274

Furthermore, supplementing dogs with RA potentially impacted pathways related to sphingolipid, phosphatidylcholine metabolism and in total lipids and fatty acids metabolisms (Table 5, Table S12, Table S15). Moreover, AC supplementation revealed a potential impact of this molecule on mainly lysine, sphingolipid and phospholipid metabolisms (Table S13, Table S16).

## Discussion

*Melissa officinalis* is widely used in ethnoveterinary practice as a valuable natural remedy for animals experiencing anxiety-related conditions. However, research concerning the impact of its dietary supplementation on canine metabolome is scarce. The purpose of this study was to assess the supplementation effect of *Melissa officinalis* leaf extract on beagles’ behavior and to reveal the metabolic pathways involved in its mechanism of action, using untargeted metabolomics. The results have been compared with those produced by the supplementation with RA, one of the main constituents of MOE, and AC, the active ingredient of Zylkene^®^, frequently prescribed by veterinarians to manage behavioral disorders [26, 34]. The findings from this study highlighted significant behavioral improvements in dogs supplemented with MOE compared to the placebo group. In particular, dogs in the MOE group exhibited notable improvements in overall activity levels, social interactions with other dogs, and responses to unfamiliar humans during walking. Additionally, these dogs exhibited more favorable behavior reactions when presented with an unfamiliar object, clearly setting their responses apart from those seen in the placebo group. These results confirmed the potential of MOE in the reduction of stress-related behaviors in dogs. Likewise, the calming effect of AC was similar to MOE. Results revealed an improvement in dogs’ behavior that was observed in AC group following a four-week period of supplementation. In accordance with literature, D’Angelo et al., 2022 have demonstrated a positive effect of AC in the treatment of obsessive-compulsive disorder in dogs [25]. It has also been demonstrated that using AC, in association with fluoxetine, reduces aggressive behaviors in dogs affected by relational disorders [26]. In addition, we found that the MOE-induced reduction in stress-linked behaviors was higher than that of RA, which is MOE’s main active substance, suggesting a synergistic effect of MOE compounds to improve dogs’ behavior, compared to RA alone. In agreement with this suggestion, several compounds, previously identified in *Melissa officinalis* and supported by the present findings such as chlorogenic acid and caffeic acid have already been described for their calming properties [35, 36]. Thus, it is not unreasonable to assume that their effects could have acted synergistically to enhance the action of RA in MOE group. Nonetheless, it should be pointed out that, *Melissa officinalis* leaf extract also contains other minor bioactive compounds such as flavonoids, terpenoids, tannins, and essential oils [21, 37]. For instance, *Cannabis sativa*, which has garnered considerable interest in veterinary research, also contains a wide range of biologically active compounds, many of which have shown promising therapeutic effects in various veterinary applications [38, 39]. Therefore, we cannot theoretically rule out the possibility that diverse compounds of MOE have contributed to the overall MOE effect by mechanisms that warrant further investigation.

Several *Melissa officinalis* compounds exhibit an anxiolytic effect by inhibiting GABA transaminase (GABA-T), resulting in an increase of GABA levels in the brain [20]. GABA quantification remains difficult to achieve because of its polarity, retention and low quantity [40]. To this end, GHB, one of the main metabolites synthesized from GABA, was quantified to ascertain whether the observed calming effect is linked to GABA biosynthesis pathway. We hypothesized that GABA-T inhibition will lead to an accumulation of GABA, and consequently, a decrease of GABA products, such as GHB. Indeed, results from this study revealed a decrease of GHB level from week 0 to week 4, in dogs supplemented with MOE, AC, and RA, in comparison to the placebo group. To the best of our knowledge, GHB quantification in dogs to evaluate the calming effect of a natural product has never been done. In subsequent research, this parameter could be interesting to measure as an indirect marker of GABA. Further studies are needed to confirm this hypothesis.

Other GABA-independent mechanisms regarding the MOE-induced calming effect were previously reported [18, 41] but their precise contribution remained incompletely elucidated. To gain a larger view, untargeted metabolomics was used to contribute to the characterization of mechanism of action and to investigate the metabolic changes brought by MOE supplementation in comparison to RA. Using HMDB and ImPaLA for pathway analysis, we found that several pathways were impacted by MOE and RA supplementation. We focused, in this discussion, on the pathways that could be related to the calming and anxiolytic-like effects.

Results obtained on the metabolic pathways impacted of HPO extracts analysis from MOE group demonstrated, surprisingly, several impacted pathways mainly associated to bile acids (BAs) and lipids, notably cholesterol and sphingolipid metabolisms. Effectively, research findings have proven alterations in lipids and cholesterol levels following *Melissa officinalis* supplementation [42–44]. A study carried out on hyperlipidemic rats demonstrated that the use of a *Melissa officinalis* extract significantly reduced cholesterol and lipid levels in the serum of treated hyperlipidemic rats compared to the control [42]. Another study published in 2020 revealed that a herbal extract of *Melissa officinalis* reduced serum lipid levels in obese rats [43]. Although these studies highlight the effect of *Melissa officinalis* on hyperlipidemia in rats, research on its impact on lipids levels in dogs is scarce or even non-existent to the best of our knowledge. However, several studies associated lipid homeostasis to psychiatric disorders such as anxiety and depression [45–47]. As an example, patients with mood disorders often exhibit alterations in cholesterol metabolism [48]. Other lipids such as phospholipids and sphingolipids were also related to anxiety and depression [49]. For instance, Puurunen et al., 2016 revealed, using non-targeted metabolomics on the whole blood samples, a decrease in several phospholipids in fearful dogs [30]. Nonetheless, further investigations are needed to clarify the precise correlations and mechanism of action linking lipids to psychiatric disorders. Lipids metabolism, especially cholesterol homeostasis pathways, are also related to bile acids levels. Surprisingly, in this study, the use of MOE as a supplement for dogs impacted mostly conjugated bile acids with taurine. A decrease in the levels of these molecules was observed after 4 weeks of supplementation, suggesting that they might be deconjugated. According to literature, bile acids are synthesized, conjugated to taurine or glycine in the liver, and transported to the gut where they are deconjugated by the action of bile salt hydrolase (BSH), a gastrointestinal bacteria enzyme found in a variety of genera [50, 51]. BAs deconjugation leads to high levels of free taurine and unconjugated BAs [52]. Taurine is considered as an inhibitory neurotransmitter that can be transported to the brain to act as an agonist of the GABA receptors [53–55]. Furthermore, taurine acts as an agonist of glycine receptor, one of the main mediators responsible for synaptic inhibition in several brain regions [56]. In addition, several unconjugated BAs such as cholic acid and chenodeoxycholic acid could cross the blood brain barrier (BBB) and block NMDA, an excitatory receptor [51, 57]. Furthermore, a recent comprehensive review highlighted the significant role of BAs in conveying signals to the brain, thus influencing critical brain functions such as emotional regulation [58]. Another recent study that aligns with these findings demonstrated changes in bile acid concentrations, notably an increase of taurodeoxycholic acid in fearful dogs [59]. All the above findings, along with literature, suggest a mechanism of action of *Melissa officinalis* extract through the gut-brain axis which needs supplementary studies to furthering our understanding of the MOE-induced calming action.

Concordantly, RA supplementation affected fatty acids and lipids metabolism, mainly phospholipids and sphingolipids. Literature showed that RA treatment of high fat diet mice decreased cholesterol and triglycerides levels and impacted fatty acids metabolism by facilitating their oxidation [60]. The behavioral analysis didn’t show any statistically significant behavioral improvement between placebo and RA group, likely due to the use of isolated RA rather than the whole plant extract. This suggests that the presence of additional bioactive compounds in the whole plant might be necessary to produce observable behavioral benefits through synergistic effects. Nevertheless, despite the lack of behavioral changes, metabolomic analysis indicated that RA still had an impact on metabolic pathways. Despite the absence of macroscopic behavioral changes, previous research demonstrated that RA exhibits anxiolytic and antidepressive effects [61–63]. These effects involve a multitude of mechanisms, including the reduction of corticosterone levels, which is crucial for regulating stress responses [54–56]. However, it remains unclear whether the anxiolytic effects of RA could suggest an involvement of lipid metabolism. A study published in 2023 found that while RA enhanced cognitive functions in young adult mice, it also led to disturbances in their lipid metabolism [64]. Nevertheless, the precise nature of this relationship and the underlying mechanisms are not yet fully understood and require further investigation.

Regarding AC, even though HPO extract in ESI^−^ did not reveal any significant metabolome differences, HPO and HPI in ESI^+^ demonstrated that AC supplementation affected pathways related to sphingolipids biosynthesis. One of AC mechanisms of action responsible of the anxiolytic effect in the brain is the increase of c-Fos expression, a marker for neuronal activity [34]. Interestingly, sphinganine, a metabolite in sphingolipid biosynthesis, has been found to physically interact with c-Fos, therefore, increasing the expression of its target genes [65].

In addition, several articles related sphingolipids to anxiety and depression. For instance, Metabolizing enzymes of sphingolipids were up-regulated in the rat brain with high anxiety-like behavior [59]. Furthermore, sphingolipids are considered as potential biomarkers for several psychiatric disorders [66, 67]. Determining whether the anxiolytic effect of AC is mediated by sphingolipids remains an intriguing question yet to be fully explored.

## Conclusions

Our study demonstrates that dietary supplementation of MOE significantly improved specific aspects of observed dogs’ behavior after four weeks of administration. MOE showed behavioral advantage over RA, which is MOE main hydroxycinnamic acid, and AC. This behavioral improvement correlated with a decrease in MOE group’s plasma GHB blood level, potentially indicating increased GABA accumulation in dogs’ brains. Leveraging a metabolomic approach, our findings suggested a novel mechanism of action of MOE possibly linked to the regulation of lipids and bile acids metabolism via the gut-brain axis. Although the standardized evaluation grid permitted the study of MOE’s effect on dogs’ behavior, the small sample size precluded consideration of sex as variable (Fig. S4). Further experiments with a larger number of dogs are needed to investigate potential sex effect and confirm these results.

## Electronic supplementary material

Below is the link to the electronic supplementary material.

Supplementary Material 1

## Data Availability

All data analyzed during this study are included in this article and in its supplementary information files.

## Abbreviations

AC: α-casozepine
ALP: Alkaline phosphatase
ALT: Alanine aminotransferase
ANOVA: Analysis of variance
AST: Aspartate aminotransferase
BAs: Bile acids
BBB: Blood brain barrier
BH4: Tetrahydrobiopterin
BSH: Bile salt hydrolase
EDTA: Aminopolycarboxylic acid
Eos: Eosinophils
ESI: Electrospray ionization
GABA: γ-aminobutyric acid
GC-MS: Gaz chromatography– mass spectrometry
GHB: 4-hydroxybyturic acid
Hb: Hemoglobin
HCT: Hematocrit
HMDB: Human metabolome database
HPI: Hydrophilic extract
HPLC-MS: High-Performance Liquid Chromatography coupled with Mass Spectrometry
HPLC-UV: High performance liquid chromatography– Ultra-Violet
HPO: Hydrophobic extract
ID: Identifier
ImPALA: Integrated Molecular Pathway Level Analysis
IS: Internal standard
LOD: Limit of detection
LOQ: Limit of quantification
Lymphs: Lymphocytes
MCV: Mean corpuscular volume
MCHC: mean corpuscular hemoglobin concentration
MHC: mean corpuscular hemoglobin
MOE: Melissa officinalis extract
MoNA: MassBank of North America
Mono: Monocytes
MRM: Multiple Reaction monitoring
MTBSTFA: N-tert-butyldimethylsilyl-N-methyltrifluoroacetamide
Neu: Neutrophils
NMDA: N-methyl-D-aspartate
NO: Nitric oxide
OPLS-DA: Orthogonal partial least squares-discriminant analysis
PLS-DA: Partial least squares-discriminant analysis
PLT: Platelets
QC: Quality control
QTOF-HRMS: Quadrupole time-of-flight-high resolution mass spectrometry
RA: Rosmarinic acid
RBC: Red blood cell
RDI: Red cell distribution index
Rt: Retention time
UPLC: Ultra-high performance liquid chromatography

## References

[1] Fatjó J, Ruiz-de-la-Torre J, Manteca X. The epidemiology of behavioural problems in dogs and cats: A survey of veterinary practitioners. Anim Welf 1 Mai 2006;15.

[2] Mikkola S, Salonen M, Puurunen J, Hakanen E, Sulkama S, Araujo C et al. Aggressive behaviour is affected by demographic, environmental and behavioural factors in purebred dogs. Sci Rep. 3 mai. 2021;11(1):9433. 10.1038/s41598-021-88793-5 PMC8093277 33941802

[3] Wells DL, Hepper PG. Prevalence of behaviour problems reported by owners of dogs purchased from an animal rescue shelter. Appl Anim Behav Sci 1 Août. 2000;69(1):55–65. 10.1016/s0168-1591(00)00118-0 10856784

[4] Seksel K, Lindeman MJ. Use of Clomipramine in treatment of obsessive-compulsive disorder, separation anxiety and noise phobia in dogs: a preliminary, clinical study. Aust Vet J Avr. 2001;79(4):252–6. 10.1111/j.1751-0813.2001.tb11976.x 11349411

[5] Gaultier E, Bonnafous L, Bougrat L, Lafont C, Pageat P. Comparison of the efficacy of a synthetic dog-appeasing pheromone with Clomipramine for the treatment of separation-related disorders in dogs. Vet Rec 23 Avr. 2005;156(17):533–8. 10.1136/vr.156.17.533 15849342

[6] Titeux E, Padilla S, Paragon BM, Gilbert C. Effects of a new dietary supplement on behavioural responses of dogs exposed to mild stressors. Vet Med Sci Sept. 2021;7(5):1469–82. 10.1002/vms3.560 PMC8464231 34236774

[7] van Haaften KA, Grigg EK, Kolus C, Hart L, Kogan LR. A survey of dog owners’ perceptions on the use of psychoactive medications and alternatives for the treatment of canine behavior problems. J Veterinary Behav 1 Janv. 2020;35:27–33.

[8] Cannas S, Tonini B, Belà B, Di Prinzio R, Pignataro G, Di Simone D, et al. Effect of a novel nutraceutical supplement (Relaxigen pet dog) on the fecal Microbiome and stress-related behaviors in dogs: A pilot study. J Veterinary Behav Mars. 2021;42:37–47.

[9] Masic A, Landsberg G, Milgram B, Merali Z, Durst T, Sanchez Vindas P et al. Efficacy of Souroubea-Platanus Dietary Supplement Containing Triterpenes in Beagle Dogs Using a Thunderstorm Noise-Induced Model of Fear and Anxiety. Molecules. 3 avr. 2021;26(7):2049. 10.3390/molecules26072049 PMC8038379 33916654

[10] Sechi S, Di Cerbo A, Canello S, Guidetti G, Chiavolelli F, Fiore F et al. Effects in dogs with behavioural disorders of a commercial nutraceutical diet on stress and neuroendocrine parameters. Vet Rec. 7 janv. 2017;180(1):18. 10.1136/vr.103865 PMC5284471 27885066

[11] Scandurra A, Mastellone V, Pero ME, Musco N, Iommelli P, Di Lucrezia A et al. Effects of a Nutritional Supplement (DìRelaxTM) on Anxiety in Dogs in a Randomized Control Trial Design. Animals. 11 févr. 2022;12(4):435. 10.3390/ani12040435 PMC8868118 35203143

[12] Benoit S, Chaumontet C, Violle N, Boulier A, Hafeez Z, Cakir-Kiefer C et al. The Anxiolytic-like Properties of a Tryptic Hydrolysate of Bovine αs1 Casein Containing α-Casozepine Rely on GABAA Receptor Benzodiazepine Binding Sites but Not the Vagus Nerve. Nutrients. 26 mai. 2022;14(11):2212. 10.3390/nu14112212 PMC9182760 35684011

[13] Dela Peña IJI, Kim HJ, de la Peña JB, Kim M, Botanas CJ, You KY, et al. A tryptic hydrolysate from bovine milk αs1-casein enhances pentobarbital-induced sleep in mice via the GABAA receptor. Behav Brain Res 15 Oct. 2016;313:184–90. 10.1016/j.bbr.2016.07.013 27401107

[14] Greathead H. Plants and plant extracts for improving animal productivity. Proc Nutr Soc Mai. 2003;62(2):279–90. 10.1079/pns2002197 14506875

[15] Kuralkar P, Kuralkar SV. Role of herbal products in animal production - An updated review. J Ethnopharmacol 5 Oct. 2021;278:114246. 10.1016/j.jep.2021.114246 34052352

[16] Schlieck TMM, Petrolli TG, Bissacotti BF, Copetti PM, Bottari NB, Morsch VM, et al. Addition of a blend of essential oils (cloves, Rosemary and oregano) and vitamin E to replace conventional chemical antioxidants in dog feed: effects on food quality and health of Beagles. Arch Anim Nutr Oct. 2021;75(5):389–403. 10.1080/1745039X.2021.1960091 34445901

[17] Bounihi A, Hajjaj G, Alnamer R, Cherrah Y, Zellou A. In vivo potential Anti-Inflammatory activity of Melissa officinalis L. Essential oil. Adv Pharmacol Sci. 2013;2013:101759. 24381585 10.1155/2013/101759 PMC3870089

[18] Feliú-Hemmelmann K, Monsalve F, Rivera C. Melissa officinalis and Passiflora caerulea infusion as physiological stress decreaser. Int J Clin Exp Med. 26 juin. 2013;6(6):444–51. PMC3703115 23844268

[19] Zarei A, Changizi-Ashtiyani S, Taheri S, Hossaini N. A brief overview of the effects of Melissa officinalis L. Extract on the function of various body organs. Zahedan J Res Med Sci 25 juill 2015;17.

[20] Awad R, Muhammad A, Durst T, Trudeau VL, Arnason JT. Bioassay-guided fractionation of lemon balm (Melissa officinalis L.) using an in vitro measure of GABA transaminase activity. Phytother Res. 2009;23(8):1075–81. 19165747 10.1002/ptr.2712

[21] Dastmalchi K, Damien Dorman HJ, Oinonen PP, Darwis Y, Laakso I, Hiltunen R. Chemical composition and in vitro antioxidative activity of a lemon balm (Melissa officinalis L.) extract. LWT - Food Sci Technol 1 Avr. 2008;41(3):391–400.

[22] Haybar H, Javid AZ, Haghighizadeh MH, Valizadeh E, Mohaghegh SM, Mohammadzadeh A. The effects of Melissa officinalis supplementation on depression, anxiety, stress, and sleep disorder in patients with chronic stable angina. Clin Nutr ESPEN Août. 2018;26:47–52. 10.1016/j.clnesp.2018.04.015 29908682

[23] Kennedy DO, Little W, Scholey AB. Attenuation of laboratory-induced stress in humans after acute administration of Melissa officinalis (Lemon Balm). Psychosom Med Août. 2004;66(4):607–13. 10.1097/01.psy.0000132877.72833.71 15272110

[24] Wang Q, Mei J, Xie J. The effects of lemon balm (Melissa officinalis L.) essential oil on the stress response, Anti-Oxidative ability, and kidney metabolism of sea bass during live transport. Anim (Basel) 29 Janv. 2022;12(3):339. 10.3390/ani12030339 PMC8833459 35158663

[25] d’Angelo D, Sacchettino L, Carpentieri R, Avallone L, Gatta C, Napolitano F. An interdisciplinary approach for compulsive behavior in dogs: A case report. Front Vet Sci. 2022;9:801636. 35400099 10.3389/fvets.2022.801636 PMC8988433

[26] Sacchettino L, Giuliano VO, Avallone L, Napolitano F, d’Angelo D. Combining α-s1 Casozepine and Fluoxetine treatment with a behavioral therapy improves symptoms in an aggressive dog: an Italian case report. Vet Sci 4 Juill. 2023;10(7):435. 10.3390/vetsci10070435 PMC10384918 37505840

[27] Diederich C, Giffroy JM. Behavioural testing in dogs: A review of methodology in search for standardisation. Appl Anim Behav Sci Mars. 2006;97(1):51–72.

[28] Marcato M, Kenny J, O’Riordan R, O’Mahony C, O’Flynn B, Galvin P. Assistance dog selection and performance assessment methods using behavioural and physiological tools and devices. Appl Anim Behav Sci 1 Sept. 2022;254:105691.

[29] Zhang Q, Nong Y, Liu Z, Gong L. Proteinase K combining Two-Step liquid–Liquid extraction for plasma untargeted liquid Chromatography–Mass Spectrometry-Based metabolomics to discover the potential mechanism of colorectal adenoma. Anal Chem 19 Nov. 2019;91(22):14458–66. 10.1021/acs.analchem.9b03121 31613596

[30] Puurunen J, Tiira K, Lehtonen M, Hanhineva K, Lohi H. Non-targeted metabolite profiling reveals changes in oxidative stress, Tryptophan and lipid metabolisms in fearful dogs. Behav Brain Funct Déc. 2016;12(1):7. 10.1186/s12993-016-0091-2 PMC4751666 26867941

[31] Wu T, Chen Y, Yang M, Wang S, Wang X, Hu M, et al. Comparative plasma and urine metabolomics analysis of juvenile and adult canines. Front Vet Sci 9 Janv. 2023;9:1037327. 10.3389/fvets.2022.1037327 PMC9868312 36699333

[32] Kamburov A, Cavill R, Ebbels TMD, Herwig R, Keun HC. Integrated pathway-level analysis of transcriptomics and metabolomics data with IMPaLA. Bioinf 15 Oct. 2011;27(20):2917–8. 10.1093/bioinformatics/btr499 21893519

[33] Thévenot EA, Roux A, Xu Y, Ezan E, Junot C. Analysis of the human adult urinary metabolome variations with age, body mass index, and gender by implementing a comprehensive workflow for univariate and OPLS statistical analyses. J Proteome Res 7 Août. 2015;14(8):3322–35. 10.1021/acs.jproteome.5b00354 26088811

[34] Benoit S, Chaumontet C, Schwarz J, Cakir-Kiefer C, Boulier A, Tomé D et al. Anxiolytic Activity and Brain Modulation Pattern of the α-Casozepine-Derived Pentapeptide YLGYL in Mice. Nutrients. 21 mai. 2020;12(5):1497. 10.3390/nu12051497 PMC7285003 32455588

[35] Takeda H, Tsuji M, Inazu M, Egashira T, Matsumiya T. Rosmarinic acid and caffeic acid produce antidepressive-like effect in the forced swimming test in mice. Eur J Pharmacol 9 Août. 2002;449(3):261–7. 10.1016/s0014-2999(02)02037-x 12167468

[36] Bouayed J, Rammal H, Dicko A, Younos C, Soulimani R. Chlorogenic acid, a polyphenol from Prunus domestica (Mirabelle), with coupled anxiolytic and antioxidant effects. J Neurol Sci 15 Nov. 2007;262(1):77–84. 10.1016/j.jns.2007.06.028 17698084

[37] Miraj S, Rafieian-Kopaei KS. Melissa officinalis L: A review study with an antioxidant prospective. J Evid Based Complement Altern Med Juill. 2017;22(3):385–94. 10.1177/2156587216663433 PMC5871149 27620926

[38] Della Rocca G, Di Salvo A. Hemp in veterinary medicine: from feed to drug. Front Vet Sci 28 Juill. 2020;7:387. 10.3389/fvets.2020.00387 PMC7399642 32850997

[39] Sacchettino L, Gatta C, Maruccio L, Boncompagni C, Napolitano F, Avallone L, et al. Combining cannabis and melatonin treatment with a rehabilitation program improved symptoms in a dog with compulsive disorder: A case report. Res Veterinary Sci Juill. 2023;160:26–9. 10.1016/j.rvsc.2023.05.007 37245289

[40] Olesti E, Rodríguez-Morató J, Gomez-Gomez A, Ramaekers JG, de la Torre R, Pozo OJ. Quantification of endogenous neurotransmitters and related compounds by liquid chromatography coupled to tandem mass spectrometry. Talanta Janv. 2019;192:93–102. 10.1016/j.talanta.2018.09.034 30348434

[41] Yoo DY, Choi JH, Kim W, Yoo KY, Lee CH, Yoon YS, et al. Effects of Melissa officinalis L. (Lemon Balm) extract on neurogenesis associated with serum corticosterone and GABA in the mouse dentate gyrus. Neurochem Res 1 Févr. 2011;36(2):250–7. 10.1007/s11064-010-0312-2 21076869

[42] Bolkent S, Yanardag R, Karabulut-Bulan O, Yesilyaprak B. Protective role of Melissa officinalis L. extract on liver of hyperlipidemic rats: a morphological and biochemical study. J Ethnopharmacol 14 Juill. 2005;99(3):391–8. 10.1016/j.jep.2005.02.038 15946812

[43] Lee D, Shin Y, Jang J, Park Y, Ahn J, Jeong S, et al. The herbal extract ALS-L1023 from Melissa officinalis alleviates visceral obesity and insulin resistance in obese female C57BL/6J mice. J Ethnopharmacol Mai. 2020;253:112646. 10.1016/j.jep.2020.112646 32027997

[44] Lee D, Shin Y, Roh JS, Ahn J, Jeoong S, Shin SS, et al. Lemon balm extract ALS-L1023 regulates obesity and improves insulin sensitivity via activation of hepatic PPARα in High-Fat Diet-Fed obese C57BL/6J mice. Int J Mol Sci 15 Juin. 2020;21(12):4256. 10.3390/ijms21124256 PMC7352304 32549364

[45] Mehdi SMA, Costa AP, Svob C, Pan L, Dartora WJ, Talati A et al. Depression and cognition are associated with lipid dysregulation in both a multigenerational study of depression and the National Health and Nutrition Examination Survey. Transl Psychiatry. 12 mars. 2024;14(1):1–9. 10.1038/s41398-024-02847-6 PMC10928164 38467624

[46] Han AL. Association between lipid ratio and depression: a cross-sectional study. Sci Rep 13 Avr. 2022;12(1):6190. 10.1038/s41598-022-10350-5 PMC9007956 35418704

[47] Schneider M, Levant B, Reichel M, Gulbins E, Kornhuber J, Müller CP. Lipids in psychiatric disorders and preventive medicine. Neurosci Biobehavioral Reviews Mai. 2017;76:336–62. 10.1016/j.neubiorev.2016.06.002 27317860

[48] Papakostas GI, Petersen T, Mischoulon D, Hughes ME, Alpert JE, Nierenberg AA, et al. Serum cholesterol and serotonergic function in major depressive disorder. Psychiatry Res 30 Mai. 2003;118(2):137–45. 10.1016/s0165-1781(03)00066-0 12798978

[49] Müller CP, Reichel M, Mühle C, Rhein C, Gulbins E, Kornhuber J. Brain membrane lipids in major depression and anxiety disorders. Biochimica et Biophysica Acta (BBA) - Molecular and Cell Biology of Lipids. août. 2015;1851(8):1052–65. 10.1016/j.bbalip.2014.12.014 25542508

[50] Kiriyama Y, Nochi H. Physiological role of bile acids modified by the gut Microbiome. Microorganisms Janv. 2022;10(1):68. 10.3390/microorganisms10010068 PMC8777643 35056517

[51] Xing C, Huang X, Wang D, Yu D, Hou S, Cui H, et al. Roles of bile acids signaling in neuromodulation under physiological and pathological conditions. Cell Bioscience 12 Juin. 2023;13(1):106. 10.1186/s13578-023-01053-z PMC10258966 37308953

[52] Duszka K. Versatile triad alliance: bile acid, taurine and microbiota. Cells 29 Juill. 2022;11(15):2337. 10.3390/cells11152337 PMC9367564 35954180

[53] Behar TN, Smith SV, Kennedy RT, Mckenzie JM, Maric I, Barker JL. GABAB receptors mediate motility signals for migrating embryonic cortical cells. Cereb Cortex 1 Août. 2001;11(8):744–53. 10.1093/cercor/11.8.744 11459764

[54] Wu JY, Prentice H. Role of taurine in the central nervous system. J Biomed Sci 24 Août. 2010;17(Suppl 1):S1. 10.1186/1423-0127-17-S1-S1 PMC2994408 20804583

[55] Ochoa-de la Paz L, Zenteno E, Gulias-Cañizo R, Quiroz-Mercado H. Taurine and GABA neurotransmitter receptors, a relationship with therapeutic potential? Expert Rev Neurother 3 Avr. 2019;19(4):289–91. 10.1080/14737175.2019.1593827 30892104

[56] Breitinger U, Breitinger HG. Modulators of the inhibitory Glycine receptor. ACS Chem Neurosci 17 Juin. 2020;11(12):1706–25. 10.1021/acschemneuro.0c00054 32391682

[57] Schubring SR, Fleischer W, Lin JS, Haas HL, Sergeeva OA. The bile steroid Chenodeoxycholate is a potent antagonist at NMDA and GABAA receptors. Neurosci Lett Janv. 2012;506(2):322–6. 10.1016/j.neulet.2011.11.036 22155097

[58] Chen S, Shao Q, Chen J, Lv X, Ji J, Liu Y, et al. Bile acid signalling and its role in anxiety disorders. Front Endocrinol (Lausanne) 23 Nov. 2023;14:1268865. 10.3389/fendo.2023.1268865 PMC10710157 38075046

[59] Sacchettino L, Costanzo M, Veneruso I, D’Argenio V, Mayer M, Napolitano F et al. Altered microbiome and metabolome profiling in fearful companion dogs: An exploratory study. Brundage CM, éditeur. PLoS ONE. 15 janv. 2025;20(1):e0315374. 10.1371/journal.pone.0315374 PMC11734960 39813205

[60] Nyandwi JB, Ko YS, Jin H, Yun SP, Park SW, Kim HJ. Rosmarinic acid exhibits a lipid-Lowering effect by modulating the expression of reverse cholesterol transporters and lipid metabolism in High-Fat Diet-Fed mice. Biomolecules Oct. 2021;11(10):1470. 10.3390/biom11101470 PMC8533102 34680102

[61] Makhathini KB, Mabandla MV, Daniels WMU. Rosmarinic acid reverses the deleterious effects of repetitive stress and Tat protein. Behav Brain Res Nov. 2018;353:203–9. 10.1016/j.bbr.2018.07.010 30029998

[62] Alegría-Herrera E, Herrera-Ruiz M, Román-Ramos R, Zamilpa A, Santillán-Urquiza MA, Aguilar MI, et al. Effect of *Ocimum basilicum*, *Ocimum selloi*, and Rosmarinic acid on cerebral vascular damage in a chronic hypertension model. Biol Pharm Bull 1 Févr. 2019;42(2):201–11. 10.1248/bpb.b18-00574 30713252

[63] Nie H, Peng Z, Lao N, Wang H, Chen Y, Fang Z, et al. Rosmarinic acid ameliorates PTSD-like symptoms in a rat model and promotes cell proliferation in the hippocampus. Progress Neuro-Psychopharmacology Biol Psychiatry Juin. 2014;51:16–22. 10.1016/j.pnpbp.2014.01.002 24418162

[64] Musillo C, Giona L, Ristow M, Zarse K, Siems K, Di Francesco A, et al. Rosmarinic acid improves cognitive abilities and glucose metabolism in aged C57Bl/6 N mice while disrupting lipid profile in young adults in a Sex-Dependent fashion. Nutrients Janv. 2023;15(15):3366. 10.3390/nu15153366 PMC10421458 37571303

[65] Ma S, Sandhoff R, Luo X, Shang F, Shi Q, Li Z, et al. Serine enrichment in tumors promotes regulatory T cell accumulation through sphinganine-mediated regulation of c-Fos. Sci Immunol 19 Avr. 2024;9(94):eadg8817. 10.1126/sciimmunol.adg8817 38640251

[66] Zoicas I, Mühle C, Schmidtner AK, Gulbins E, Neumann ID, Kornhuber J. Anxiety and depression are related to higher activity of sphingolipid metabolizing enzymes in the rat brain. Cells 17 Mai. 2020;9(5):1239. 10.3390/cells9051239 PMC7290887 32429522

[67] van Kruining D, Luo Q, van Echten-Deckert G, Mielke MM, Bowman A, Ellis S, et al. Sphingolipids as prognostic biomarkers of neurodegeneration, neuroinflammation, and psychiatric diseases and their emerging role in lipidomic investigation methods. Adv Drug Delivery Reviews 1 Janv. 2020;159:232–44. 10.1016/j.addr.2020.04.009 PMC7665829 32360155

