# Supplementary material for: Metabolomics provides novel understanding of Melissa officinalis mechanism of action ensuring its calming effect on dogs
Source: BMC Vet Res. 2025 Jul 11;21:459. doi: 10.1186/s12917-025-04904-8 (PMC12247294; doi:10.1186/s12917-025-04904-8)
Supplement: Supplementary file 1 — Supplementary Material 1 [file 12917_2025_4904_MOESM1_ESM.docx]

**Supplementary data:**


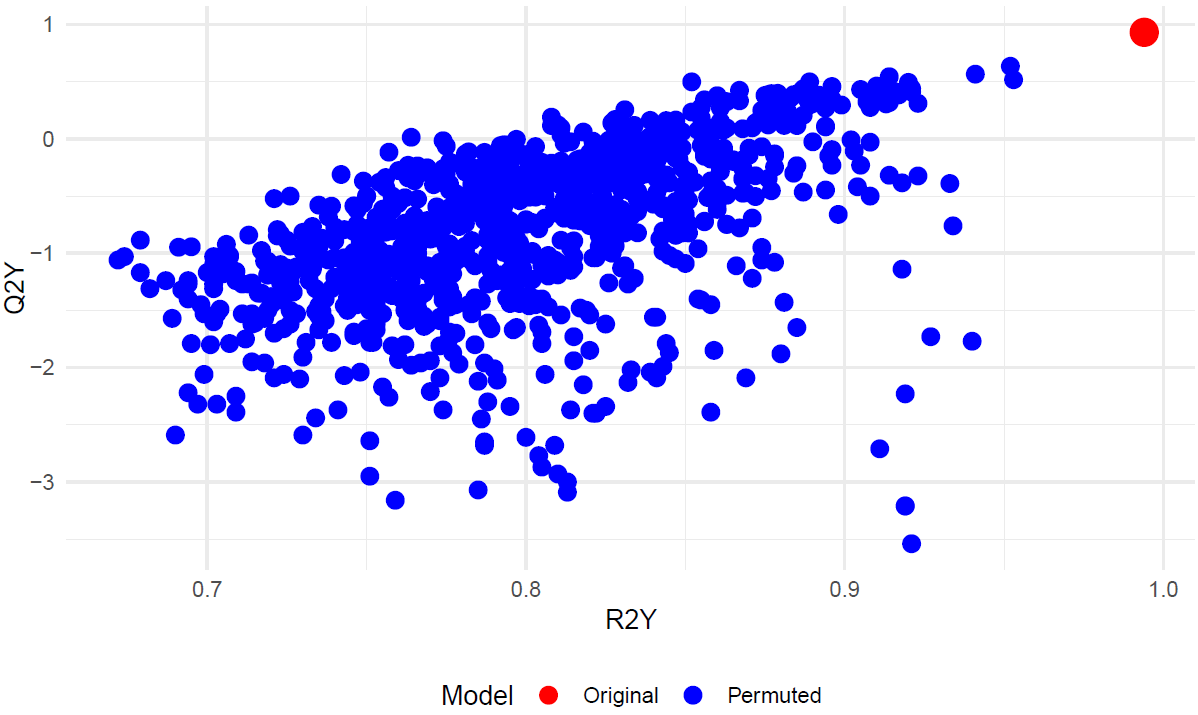

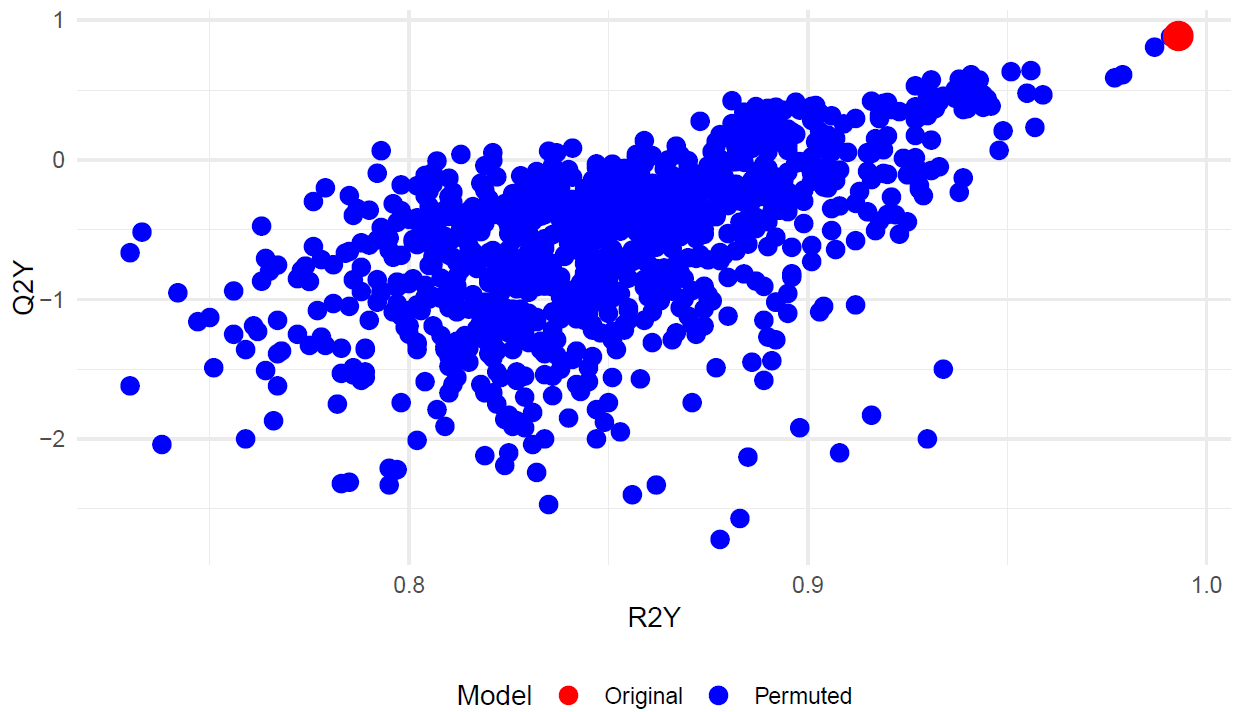


B


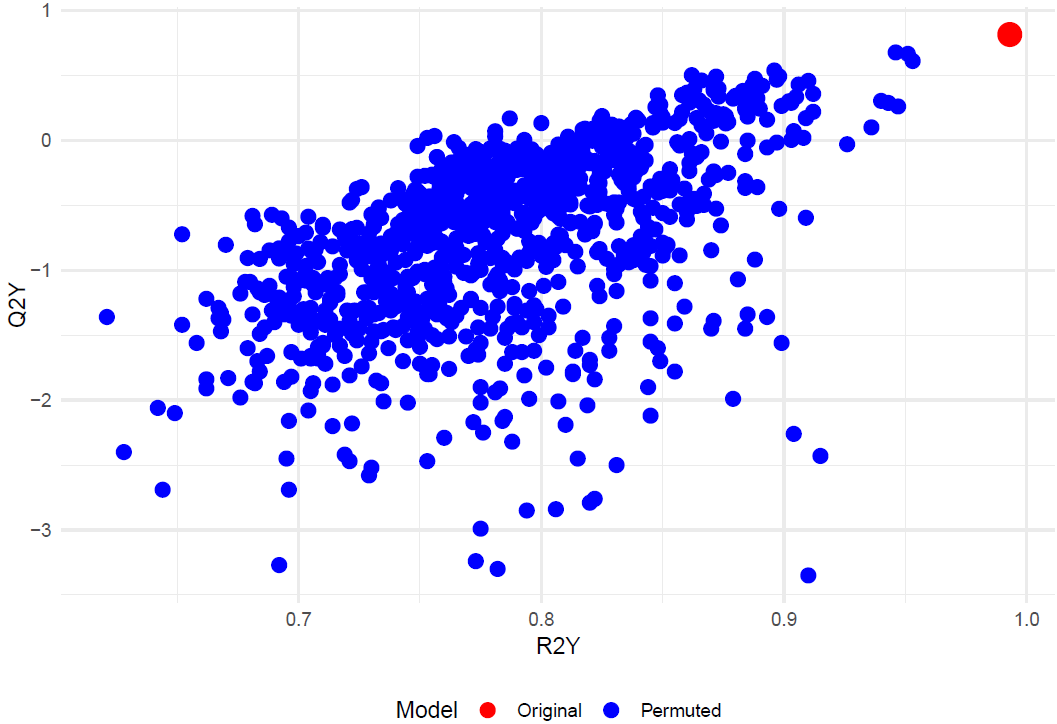


C


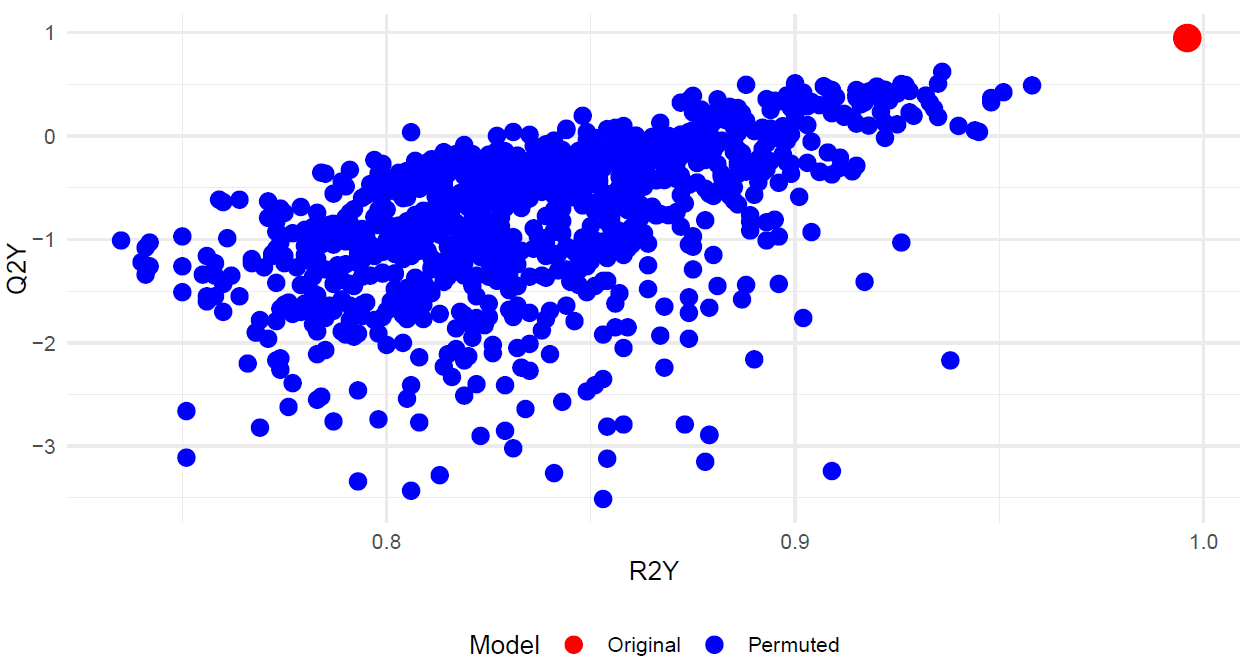


D

E


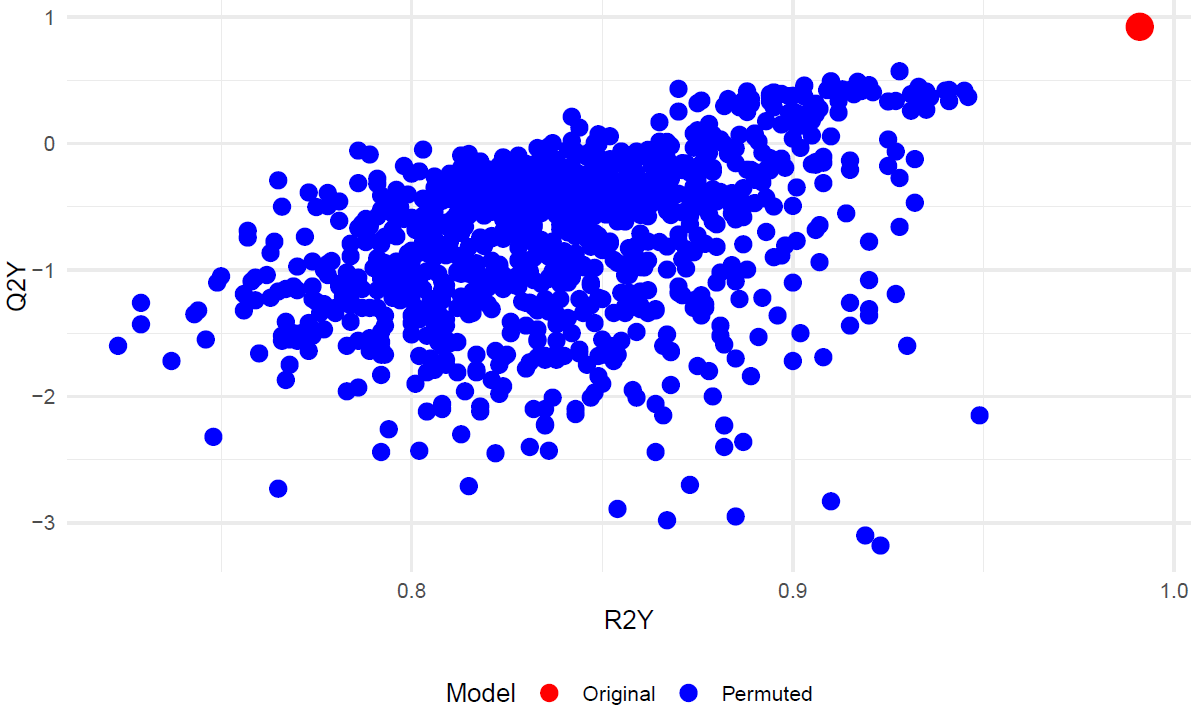


F


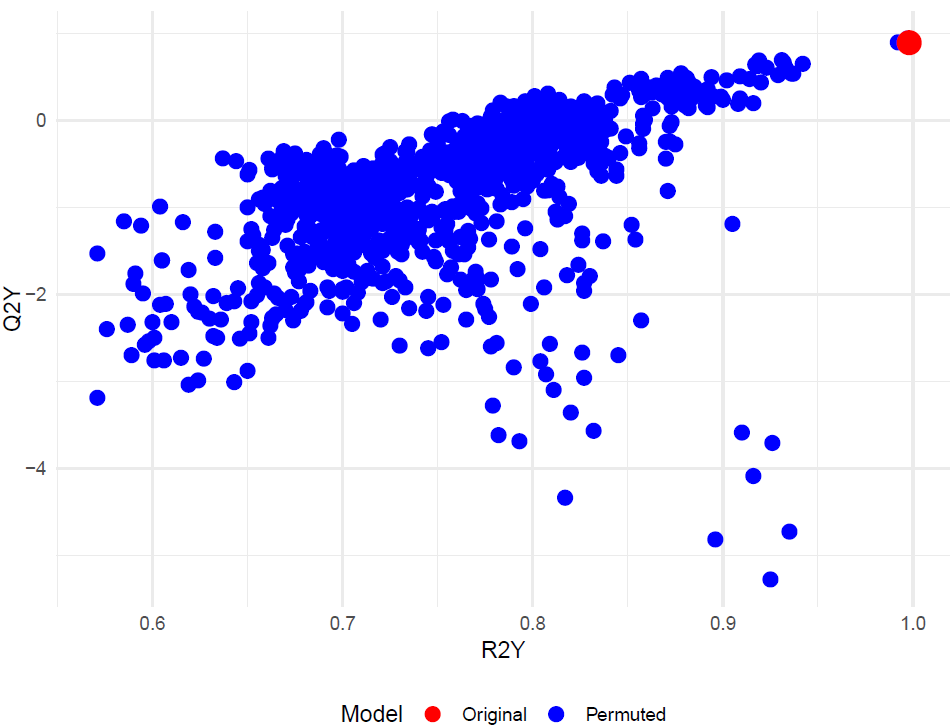


G


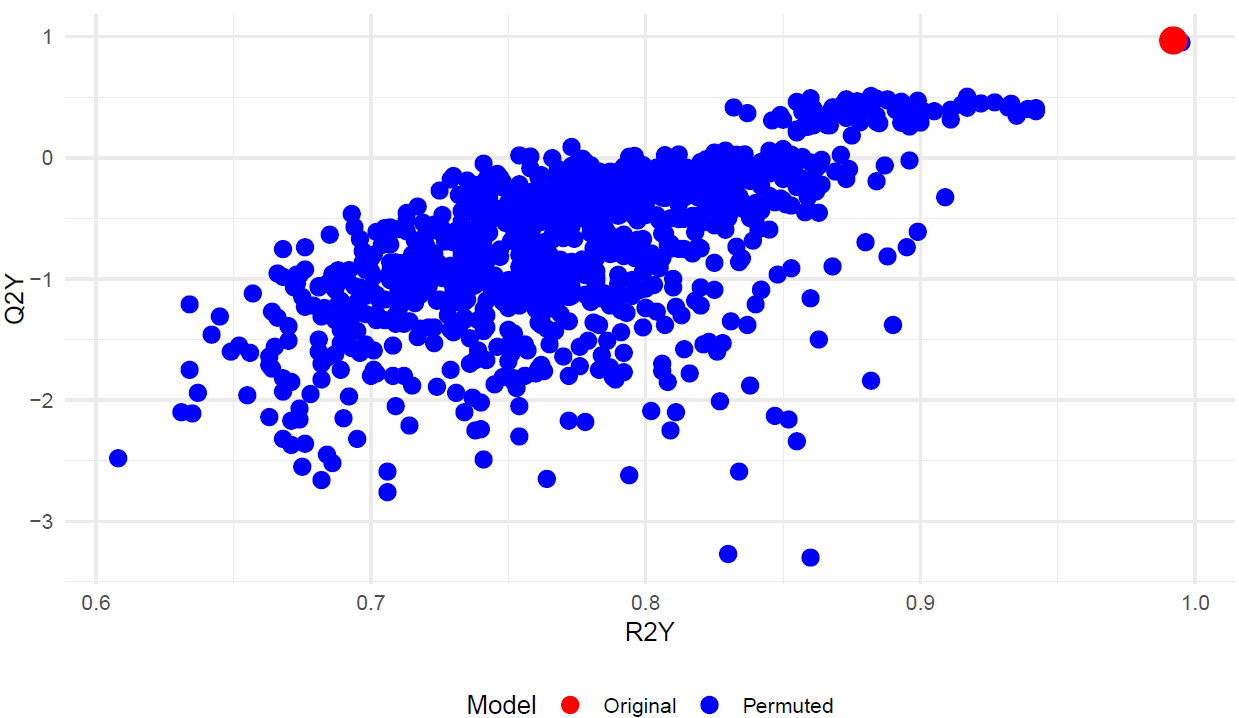


H


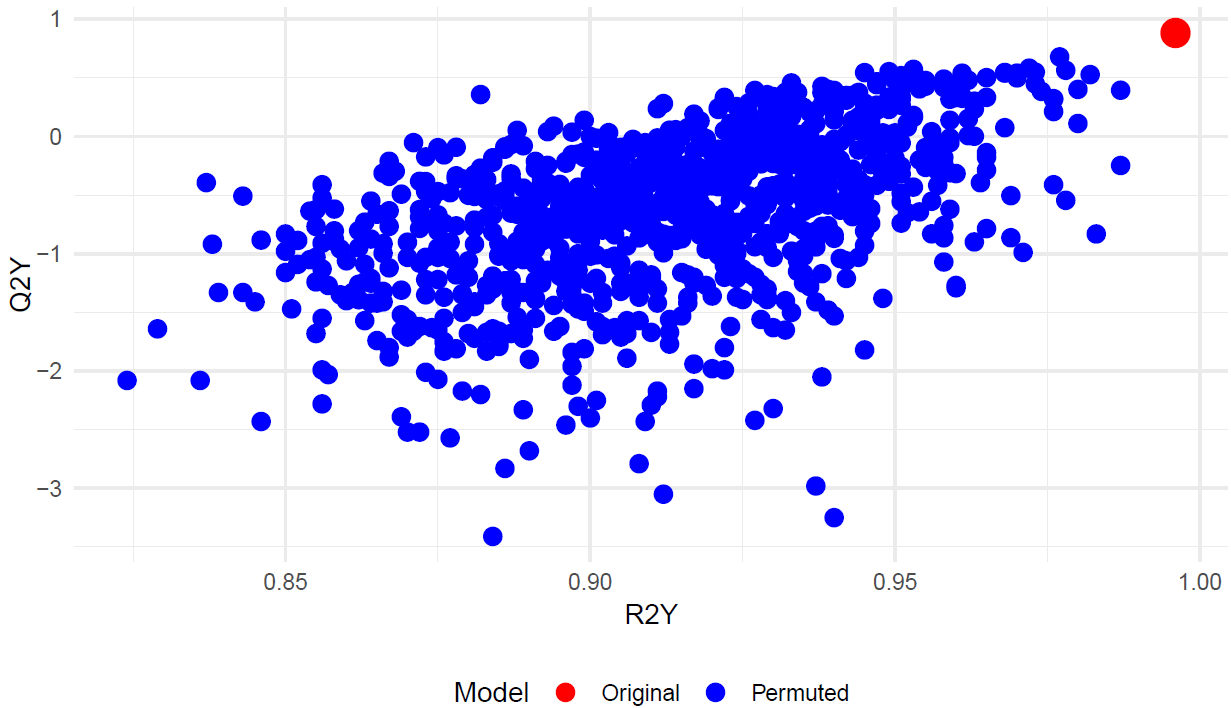


A

S1: Permutations tests of hydrophobic extracts (HPO) in ESI^+^ and ESI^-^ and hydrophilic extracts (HPI) in ESI^+^ modes. (A) Placebo and *Melissa officinalis* extract (MOE) comparison of HPO extracts in ESI^-^ mode. (B) Placebo and rosmarinic acid (RA) comparison of HPO extracts in ESI^-^ mode. (C) Placebo and MOE comparison of HPO extracts in ESI^+^ mode. (D) Placebo and RA comparison of HPO extracts in ESI^+^ mode. (E) Placebo and α-casozepine (AC) comparison of HPO extracts in ESI^+^ mode. (F) Placebo and MOE comparison of HPI extracts in ESI^+^ modes. (G) Placebo and RA comparison of HPI extracts in ESI^+^ mode. (H) Placebo and AC comparison of HPI extracts in ESI^+^ mode.


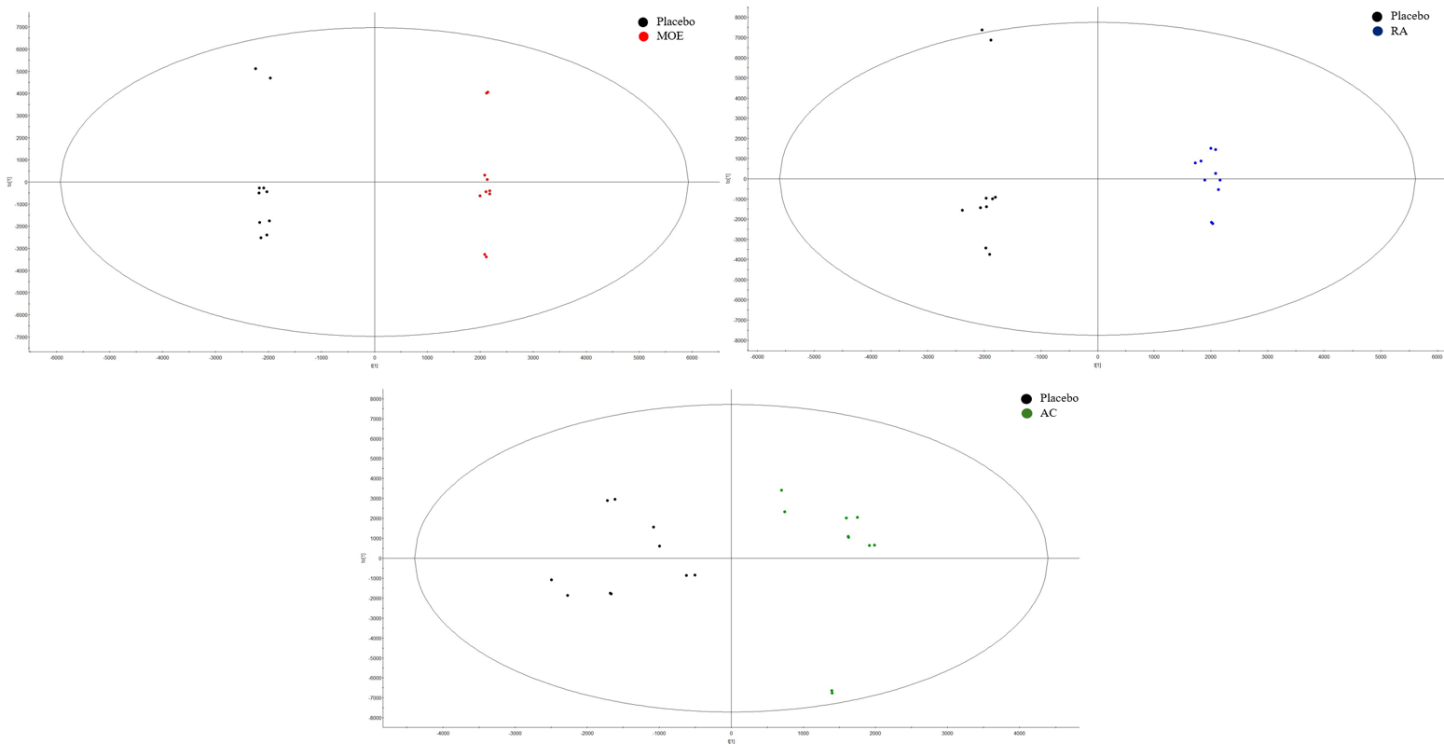


Figure S2 : Metabolomic profiles of beagle dogs’ plasma generated by OPLS-DA of HPO extracts in ESI^+^ mode. (A) Placebo and MOE comparison; (B) placebo and AC comparison; (C) placebo and RA comparison.


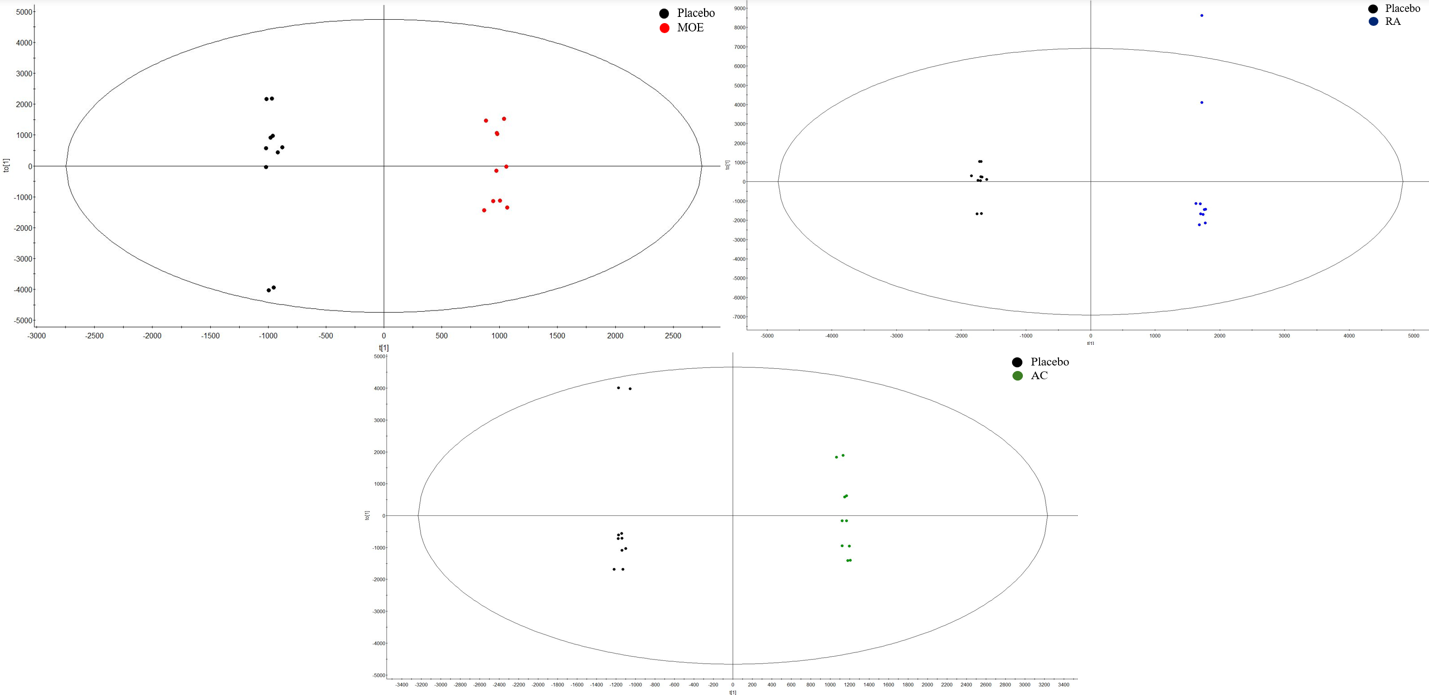


Figure S3: Metabolomic profiles of beagle dogs’ plasma generated by OPLS-DA of HPI extracts in ESI^+^ mode. (A) Placebo and MOE comparison; (B) placebo and AC comparison; (C) placebo and RA comparison.


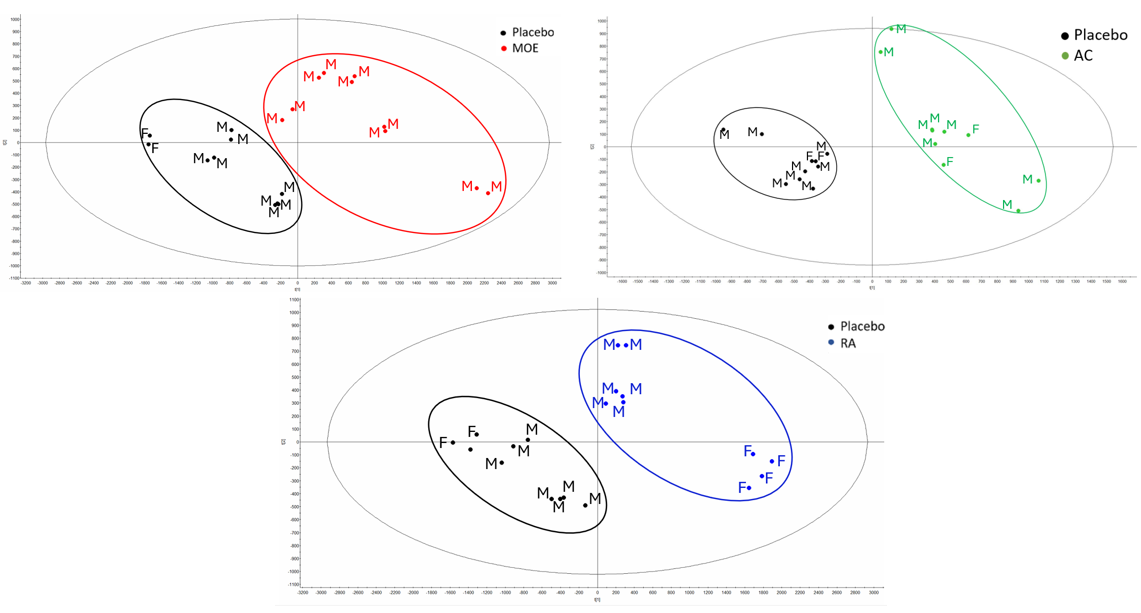


**A**

**C**

**B**

Figure S4: Metabolomic profiles of females (F) and males (M) beagle dogs’ plasma generated by PLS-DA of HPO extracts in ESI^-^ mode, with (A) Placebo and MOE comparison; (B) placebo and AC comparison; (C) placebo and RA comparison.

Table S1: Evaluation of MOE, RA, and AC effects on Beagle dogs’ behavior in physical and physiological domains. Data are presented as a means of scores for week 0 and week 4 (n=5).

| Groups | Weeks | Physical domain | Psychological domain |
| --- | --- | --- | --- |
| **Placebo** | 0 | 3.95 | 3.90 |
|  | 4 | 3.90 | 3.90 |
| **MOE** | 0 | 3.95 | 3.90 |
|  | 4 | 4.00 | 4.00 |
| **RA** | 0 | 4.00 | 4.00 |
|  | 4 | 4.00 | 3.80 |
| **AC** | 0 | 4.00 | 3.90 |
|  | 4 | 3.90 | 4.00 |

Table S2: Permutation tests parameters for comparisons of HPO and HPI extracts in ESI^+^ mode.

| HPO in ESI^+^ | Placebo vs MOE | R2Y=0.993 and Q2Y=0.812 |
| --- | --- | --- |
|  | Placebo vs RA | R2Y=0.996 and Q2Y=0.948 |
|  | Placebo vs AC | R2Y=0.994 and Q2Y=0.931 |
| HPI in ESI^+^ | Placebo vs MOE | R2Y=0.991, Q2Y=0.924 |
|  | Placebo vs RA | R2Y=0.998, Q2Y=0.895 |
|  | Placebo vs AC | R2Y=0.992, Q2Y=0.968 |

Table S3: List of identified metabolites between placebo and MOE of HPO extracts in ESI^-^ mode.

| **m/z** | **RT (min)** | **HMDB ID** | **Accepted Description** | **ppm** | **Score** | **Trend** |
| --- | --- | --- | --- | --- | --- | --- |
| 538.2256 | 0.47 | HMDB0248586 | Arginyl-glycyl-aspartyl-phenylalanine | -2.19 | 38.5 | ▼ |
| 284.0874 | 0.48 | HMDB0005923 | N4-Acetylcytidine | -4.80 | 39.1 | ▼ |
| 205.0839 | 0.48 | HMDB0029049 | Serylthreonine | 4.51 | 44.4 | ▼ |
| 547.3117 | 0.48 | HMDB0304815 | Val-Tyr-Leu-Arg | -3.15 | 40 | ▼ |
| 127.0519 | 0.55 | HMDB0062558 | Pyroglutamine | 4.69 | 40.3 | ▼ |
| 644.3207 | 0.65 | HMDB0013059 | S-(9-hydroxy-PGA1)-glutathione | -2.36 | 42 | ▼ |
| 459.2057 | 1.18 | HMDB0241920 | N-Palmitoyl Arginine | -0.17 | 40.6 | ▼ |
| 217.1200 | 1.18 | HMDB0029042 | Serylisoleucine | 2.72 | 42.9 | ▼ |
| 563.7725 | 1.28 | HMDB0279487 | PIP(LTE4/20:0) | 5.11 | 42 | ▲ |
| 448.9988 | 1.50 | HMDB0000295 | Uridine 5'-diphosphate | -4.07 | 38.2 | ▼ |
| 300.1938 | 2.07 | HMDB0028945 | Lysylarginine | 4.24 | 39.7 | ▲ |
| 535.2725 | 2.09 | HMDB0028734 | Asparaginylisoleucine | -1.67 | 42.1 | ▲ |
| 821.3792 | 2.25 | HMDB0274844 | PGP(18:1(9Z)-O(12,13)/i-12:0) | 1.72 | 40.5 | ▲ |
| 437.0545 | 2.52 | HMDB0001117 | 4'-Phosphopantothenoylcysteine | -2.64 | 44.9 | ▼ |
| 296.1002 | 2.60 | HMDB0005862 | 2-Methylguanosine | 0.44 | 41.7 | ▲ |
| 286.1795 | 2.63 | HMDB0031885 | 6-Hydroxypentadecanedioic acid | -0.40 | 45.1 | ▲ |
| 478.2668 | 2.93 | HMDB0012932 | Dynorphin A (6-8) | 1.26 | 44.2 | ▲ |
| 701.3424 | 2.96 | HMDB0262739 | PA(10:0/PGF1alpha) | -2.10 | 47.8 | ▲ |
| 286.1780 | 3.14 | HMDB0248583 | Arginine ornithine | 3.70 | 43.6 | ▲ |
| 595.3031 | 3.21 | HMDB0266620 | PA(PGJ2/8:0) | -1.69 | 43.6 | ▲ |
| 804.4465 | 3.28 | HMDB0285033 | PE(20:5(7Z,9Z,11E,13E,17Z)-3OH(5,6,15)/DiMe(9,3)) | 0.94 | 46.4 | ▲ |
| 662.2942 | 3.32 | HMDB0002579 | Glycochenodeoxycholic acid 3-glucuronide | -1.04 | 48.4 | ▼ |
| 1050.5348 | 3.33 | HMDB0277932 | PI(22:4(10Z,13Z,16Z,19Z)/LTE4) | -3.25 | 43.1 | ▲ |
| 1007.5884 | 3.64 | HMDB0286968 | PC(20:0/LTE4) | -4.94 | 45.3 | ▲ |
| 616.3641 | 3.81 | HMDB0011493 | LysoPC(22:4(7Z-10Z-13Z-16Z)/0:0) | 3.63 | 52 | ▼ |
| 502.2508 | 3.88 | HMDB0247848 | Lysyl-aspartyl-glutamyl-leucine | -2.13 | 40.6 | ▲ |
| 734.9162 | 4.13 | HMDB0004914 | Ganglioside GD3 (d18:1/18:0) | 4.76 | 40.6 | ▲ |
| 738.4513 | 4.13 | HMDB0009203 | PE(18:4(6Z,9Z,12Z,15Z)/20:5(5Z,8Z,11Z,14Z,17Z)) | 1.21 | 49.7 | ▲ |
| 1064.4825 | 4.18 | HMDB0290906 | CDP-DG(TXB2/16:1(9Z)) | -3.94 | 44.4 | ▼ |
| 486.2746 | 4.20 | HMDB0242015 | N-Docosahexaenoyl Histidine | 1.76 | 46.6 | ▲ |
| 649.3540 | 4.28 | HMDB0262728 | PA(22:6(4Z,7Z,11E,13Z,15E,19Z)-2OH(10S,17)/10:0) | 4.28 | 47.1 | ▼ |
| 867.4932 | 4.30 | HMDB0285722 | PC(14:1(9Z)/LTE4) | -3.62 | 48.9 | ▲ |
| 683.3561 | 4.40 | HMDB0262736 | PA(PGE1/10:0) | 2.89 | 48.3 | ▼ |
| 295.1129 | 4.42 | HMDB0029001 | Phenylalanylmethionine | 2.39 | 43.5 | ▲ |
| 769.4050 | 4.45 | HMDB0264379 | PA(20:4(8Z,11Z,14Z,17Z)-2OH(5S,6R)/18:4(6Z,9Z,12Z,15Z)) | -1.62 | 42.2 | ▲ |
| 996.5194 | 4.65 | HMDB0278247 | PI(22:5(7Z,10Z,13Z,16Z,19Z)/TXB2) | -3.30 | 45.7 | ▼ |
| 958.5090 | 4.76 | HMDB0283361 | PS(22:6(5Z,8E,10Z,13Z,15E,19Z)-2OH(7S, 17S)/22:5(4Z,7Z,10Z,13Z,16Z)) | 0.28 | 50.4 | ▲ |
| 620.3602 | 4.81 | HMDB0297131 | DG(8:0/LTE4/0:0) | -3.90 | 46.5 | ▼ |
| 1053.5525 | 4.83 | HMDB0115993 | CDP-DG(20:4(5Z,8Z,11Z,14Z)/20:1(11Z)) | 5.07 | 44.2 | ▼ |
| 1038.3911 | 4.86 | HMDB0293327 | CDP-DG(i-12:0/22:6(5Z,8E,10Z,13Z,15E,19Z)-2OH(7S, 17S)) | 0.96 | 44.6 | ▼ |
| 1077.5334 | 4.91 | HMDB0280323 | PIP(22:3(10Z,13Z,16Z)/20:4(7E,9E,11Z,13E)-3OH(5S,6R,15S)) | 1.13 | 48.2 | ▲ |
| 1028.4563 | 4.93 | HMDB0276166 | PI(16:1(9Z)/LTE4) | -1.49 | 44.6 | ▼ |
| 745.4207 | 4.98 | HMDB0266724 | PA(PGJ2/P-16:0) | -1.37 | 46.4 | ▲ |
| 920.4832 | 5.00 | HMDB0116782 | PS(22:4(7Z,10Z,13Z,16Z)/22:6(4Z,7Z,10Z,13Z,16Z,19Z)) | -1.94 | 52.1 | ▲ |
| 617.1779 | 5.01 | HMDB0304275 | biliverdin-IX-alpha | -4.92 | 47.6 | ▼ |
| 392.1849 | 5.08 | HMDB0241247 | 3-Hydroxydodeca-5,7-dienoylcarnitine | 1.11 | 41.5 | ▲ |
| 690.3304 | 5.08 | HMDB0013055 | S-(11-hydroxy-9-deoxy-delta12-PGD2)-glutathione | 4.14 | 45.2 | ▲ |
| 955.4133 | 5.09 | HMDB0273149 | PGP(18:3(9Z,12Z,15Z)/PGD2) | -1.41 | 47.1 | ▲ |
| 469.2298 | 5.09 | HMDB0011180 | Prolylproline | -1.46 | 38.9 | ▲ |
| 852.4553 | 5.16 | HMDB0262371 | PE(PGJ2/20:4(5Z,8Z,11Z,14Z)) | -4.28 | 52.7 | ▲ |
| 831.5179 | 5.20 | HMDB0265826 | PA(22:2(13Z,16Z)/22:6(5Z,8E,10Z,13Z,15E,19Z)-2OH(7S, 17S)) | -0.28 | 42.8 | ▲ |
| 726.4518 | 5.21 | HMDB0266718 | PA(PGD2/P-16:0) | 4.26 | 44.2 | ▲ |
| 1002.4854 | 5.25 | HMDB0293613 | CDP-DG(20:3(8Z,11Z,14Z)-2OH(5,6)/i-15:0) | -0.82 | 43.8 | ▲ |
| 998.4578 | 5.37 | HMDB0293449 | CDP-DG(i-13:0/22:5(4Z,7Z,10Z,13Z,19Z)-O(16,17)) | 2.78 | 48.9 | ▼ |
| 608.2321 | 5.37 | HMDB0006730 | QYNAD | -0.18 | 42.5 | ▼ |
| 619.2308 | 5.40 | HMDB0000054 | Bilirubin | -3.56 | 46.5 | ▲ |
| 972.5233 | 5.40 | HMDB0277304 | PI(6 keto-PGF1alpha/20:3(5Z,8Z,11Z)) | 0.64 | 50.3 | ▲ |
| 809.4042 | 5.51 | HMDB0265414 | PA(20:5(7Z,9Z,11E,13E,17Z)-3OH(5,6,15)/20:5(5Z,8Z,11Z,14Z,17Z)) | 3.92 | 48.4 | ▲ |
| 1037.5543 | 5.51 | HMDB0277823 | PI(22:3(10Z,13Z,16Z)/6 keto-PGF1alpha) | -4.02 | 53 | ▲ |
| 893.5068 | 5.52 | HMDB0276118 | PI(16:0/18:3(9,11,15)-OH(13)) | 4.07 | 39.3 | ▲ |
| 753.4485 | 5.57 | HMDB0010586 | PG(16:1(9Z)/16:1(9Z)) | 0.83 | 40.8 | ▲ |
| 865.5138 | 5.60 | HMDB0281403 | PS(22:6(4Z,7Z,11E,13Z,15E,19Z)-2OH(10S,17)/18:0) | 2.05 | 53.3 | ▲ |
| 1064.5061 | 5.71 | HMDB0291836 | CDP-DG(PGJ2/20:2(11Z,14Z)) | 3.82 | 52.7 | ▲ |
| 845.4714 | 5.76 | HMDB0269191 | PG(18:2(9Z,12Z)/20:4(5Z,8Z,11Z,14Z)-OH(17)) | -3.39 | 50.7 | ▲ |
| 698.4178 | 5.79 | HMDB0267556 | PA(20:4(6Z,8E,10E,14Z)-2OH(5S,12R)/i-14:0) | 0.59 | 49.6 | ▲ |
| 810.4321 | 5.81 | HMDB0285732 | PC(14:1(9Z)/5-iso PGF2VI) | -1.09 | 48.5 | ▲ |
| 514.2902 | 5.86 | HMDB0256155 | PC(16:1(9E)/0:0) | -2.70 | 43.4 | ▲ |
| 650.3877 | 5.88 | HMDB0000824 | Propionylcarnitine | 1.16 | 37.2 | ▲ |
| 838.5009 | 5.88 | HMDB0112651 | PS(20:4(5Z,8Z,11Z,14Z)/22:5(7Z,10Z,13Z,16Z,19Z)) | -2.32 | 50.9 | ▲ |
| 1002.5245 | 5.90 | HMDB0291028 | CDP-DG(18:1(12Z)-O(9S,10R)/18:0) | 1.79 | 48.9 | ▲ |
| 560.2751 | 5.91 | HMDB0061698 | 1-Stearoylglycerophosphoserine | -1.88 | 44.5 | ▲ |
| 737.4561 | 5.93 | HMDB0263530 | PA(18:1(12Z)-O(9S,10R)/17:0) | 4.42 | 44.2 | ▲ |
| 873.4412 | 5.98 | HMDB0282844 | PS(20:5(7Z,9Z,11E,13E,17Z)-3OH(5,6,15)/20:5(5Z,8Z,11Z,14Z,17Z)) | -3.64 | 49.9 | ▲ |
| 1008.4611 | 6.06 | HMDB0279166 | PIP(PGE2/18:2(9Z,12Z)) | -1.72 | 50.2 | ▼ |
| 934.4965 | 6.13 | HMDB0116075 | CDP-DG(a-13:0/i-19:0) | 0.06 | 46.5 | ▲ |
| 666.4191 | 6.16 | HMDB0290080 | Cer(d18:2(4E,14Z)/20:5(7Z,9Z,11E,13E,17Z)-3OH(5,6,15)) | 7.87 | 44.9 | ▲ |
| 484.2855 | 6.18 | HMDB0000631 | Deoxycholic acid glycine conjugate | 4.46 | 47.3 | ▲ |
| 406.2342 | 6.18 | HMDB0013024 | Neurotensin 11-13 | -1.42 | 46.6 | ▲ |
| 840.4250 | 6.18 | HMDB0261785 | PE(20:5(7Z,9Z,11E,13E,17Z)-3OH(5,6,15)/18:4(6Z,9Z,12Z,15Z)) | 3.23 | 50.4 | ▲ |
| 1020.4633 | 6.34 | HMDB0274507 | PGP(a-17:0/LTE4) | -2.17 | 46.8 | ▲ |
| 408.1968 | 6.35 | HMDB0254492 | Met-leu-phe | 1.26 | 45.9 | ▲ |
| 376.2245 | 6.39 | HMDB0013631 | Oleoyl glycine | -4.29 | 41.2 | ▲ |
| 1120.5819 | 6.41 | HMDB0293082 | CDP-DG(PGF2alpha/a-21:0) | -3.32 | 44.6 | ▲ |
| 1098.5490 | 6.49 | HMDB0293121 | CDP-DG(a-21:0/20:5(7Z,9Z,11E,13E,17Z)-3OH(5,6,15)) | 4.64 | 42.1 | ▲ |
| 1114.5760 | 6.51 | HMDB0294324 | CDP-DG(PGD2/i-22:0) | 0.80 | 52.8 | ▲ |
| 723.4393 | 6.55 | HMDB0115161 | PA(20:4(5Z,8Z,11Z,14Z)/20:5(5Z,8Z,11Z,14Z,17Z)) | -0.35 | 44.7 | ▲ |
| 376.2246 | 6.57 | HMDB0241210 | 4-Dodecenoylcarnitine | -4.24 | 40.3 | ▲ |
| 737.4586 | 6.57 | HMDB0270585 | PG(18:1(12Z)-2OH(9,10)/a-13:0) | -3.35 | 44 | ▲ |
| 767.4658 | 6.61 | HMDB0265004 | PA(22:6(5Z,7Z,10Z,13Z,16Z,19Z)-OH(4)/20:3(5Z,8Z,11Z)) | 0.10 | 45.3 | ▲ |
| 683.3962 | 6.62 | HMDB0262874 | PA(13:0/PGE2) | 4.55 | 49.8 | ▲ |
| 586.3093 | 6.69 | HMDB0252757 | Glu-Ile-Leu-Asp-Val | -0.15 | 45.6 | ▲ |
| 978.4157 | 6.73 | HMDB0278693 | PIP(16:2(9Z,12Z)/20:5(7Z,9Z,11E,13E,17Z)-3OH(5,6,15)) | -0.17 | 41.8 | ▼ |
| 576.2310 | 6.74 | HMDB0002581 | Taurocholic acid 3-sulfate | 0.65 | 42.3 | ▼ |
| 773.4394 | 6.86 | HMDB0266009 | PA(22:5(4Z,7Z,10Z,13Z,16Z)/5-iso PGF2VI) | -0.60 | 45.7 | ▲ |
| 874.4314 | 6.90 | HMDB0281910 | PS(20:4(6E,8Z,11Z,13E)-2OH(5S,15S)/18:3(9Z,12Z,15Z)) | 4.29 | 49.2 | ▲ |
| 455.3261 | 6.93 | HMDB0242018 | N-Docosahexaenoyl Lysine | -4.07 | 44.1 | ▲ |
| 971.4556 | 6.98 | HMDB0278569 | PIP(16:1(9Z)/18:2(10E,12Z)+=O(9)) | 1.77 | 45.3 | ▲ |
| 881.4610 | 7.00 | HMDB0276294 | PI(16:2(9Z,12Z)/18:1(9Z)-O(12,13)) | 2.52 | 48.5 | ▲ |
| 574.3259 | 7.11 | HMDB0011490 | LysoPE(0:0/22:0) | -4.00 | 41.2 | ▲ |
| 405.2245 | 7.17 | HMDB0258096 | Adenosine, 8-(butylamino)-N-cyclopentyl- | -2.65 | 45.6 | ▲ |
| 285.1828 | 7.17 | HMDB0028712 | Arginylisoleucine | 4.06 | 50 | ▲ |
| 821.4624 | 7.17 | HMDB0269309 | PG(18:3(6Z,9Z,12Z)/PGD2) | 1.61 | 43.7 | ▲ |
| 716.4146 | 7.18 | HMDB0294683 | DG(12:0/0:0/LTE4) | -4.55 | 43.4 | ▲ |
| 477.2729 | 7.27 | HMDB0242012 | N-Docosahexaenoyl Glutamine | -1.28 | 45.9 | ▲ |
| 204.0673 | 7.32 | HMDB0001190 | Indoleacetaldehyde | 4.20 | 40 | ▼ |
| 803.4120 | 7.37 | HMDB0264332 | PA(18:4(6Z,9Z,12Z,15Z)/6 keto-PGF1alpha) | 0.47 | 44.8 | ▲ |
| 1004.4689 | 7.46 | HMDB0293561 | CDP-DG(PGD1/i-14:0) | 3.29 | 52.6 | ▲ |
| 854.4059 | 7.46 | HMDB0267657 | PA(i-15:0/LTE4) | 1.07 | 47.4 | ▲ |
| 1069.5190 | 7.56 | HMDB0280184 | PIP(20:4(5Z,8Z,11Z,14Z)-OH(20)/22:2(13Z,16Z)) | -0.10 | 52.3 | ▲ |
| 850.5424 | 7.57 | HMDB0285680 | PC(14:0/PGF1alpha) | -3.41 | 44.4 | ▲ |
| 779.5032 | 7.62 | HMDB0115370 | PA(22:5(4Z,7Z,10Z,13Z,16Z)/22:4(7Z,10Z,13Z,16Z)) | 1.42 | 40.2 | ▲ |
| 439.2090 | 7.64 | HMDB0253027 | Phenylalanyl-prolyl-arginine | 3.56 | 46.5 | ▲ |
| 870.4886 | 7.69 | HMDB0282441 | PS(20:3(5Z,8Z,11Z)/22:6(4Z,7Z,11E,13Z,15E,19Z)-2OH(10S,17)) | -4.56 | 48.5 | ▲ |
| 429.2513 | 7.71 | HMDB0242070 | N-Eicosapentaenoyl Glutamic acid | -4.20 | 43.4 | ▲ |
| 899.5070 | 7.71 | HMDB0271807 | PG(PGF2alpha/i-19:0) | 1.41 | 49.7 | ▲ |
| 866.4980 | 7.78 | HMDB0261326 | PE(18:1(9Z)/6 keto-PGF1alpha) | 2.87 | 49.9 | ▲ |
| 876.4094 | 7.85 | HMDB0249908 | Ala-Thr-Trp-Leu-Pro-Pro-Arg | -5.45 | 45.5 | ▲ |
| 912.5031 | 7.88 | HMDB0284422 | PE(22:6(4Z,7Z,10Z,13Z,16Z,19Z)/22:6(4Z,7Z,11E,13Z,15E,19Z)-2OH(10S,17)) | -0.11 | 51.9 | ▲ |
| 555.2298 | 7.92 | HMDB0266528 | PA(20:3(8Z,11Z,14Z)-2OH(5,6)/2:0) | -8.03 | 44.1 | ▲ |
| 813.4871 | 7.92 | HMDB0264485 | PA(22:6(5Z,7Z,10Z,13Z,16Z,19Z)-OH(4)/19:0) | 3.60 | 47.6 | ▲ |
| 884.5174 | 7.93 | HMDB0283955 | PE(PGJ2/22:2(13Z,16Z)) | -4.68 | 48.9 | ▲ |
| 1057.5144 | 7.95 | HMDB0280341 | PIP(22:3(10Z,13Z,16Z)/18:1(9Z)-O(12,13)) | -4.62 | 43.1 | ▼ |
| 900.4921 | 7.95 | HMDB0281931 | PS(18:3(9Z,12Z,15Z)/PGD1) | 4.81 | 49.2 | ▲ |
| 1107.4999 | 7.97 | HMDB0280365 | PIP(22:3(10Z,13Z,16Z)/22:6(4Z,7Z,10Z,13E,15E,19Z)-OH(17)) | 1.50 | 42.9 | ▲ |
| 833.4373 | 8.05 | HMDB0275109 | PGP(i-15:0/20:4(5Z,8Z,11Z,14Z)-OH(17)) | -0.24 | 52 | ▼ |
| 897.4353 | 8.32 | HMDB0276281 | PI(5-iso PGF2VI/16:2(9Z,12Z)) | -3.40 | 44.4 | ▲ |
| 633.3262 | 8.44 | HMDB0288833 | PC(PGF2alpha/2:0) | -4.98 | 43.6 | ▲ |
| 581.3077 | 8.69 | HMDB0240261 | LysoPI(18:0/0:0) | -3.15 | 40.5 | ▲ |
| 1092.5575 | 8.78 | HMDB0294017 | CDP-DG(PGF2alpha/i-19:0) | 2.91 | 45.4 | ▲ |
| 783.4629 | 8.85 | HMDB0266189 | PA(22:6(4Z,7Z,10Z,13Z,16Z,19Z)/20:4(5Z,8Z,11Z,13E)-OH(15S)) | 2.89 | 42 | ▲ |
| 542.3348 | 8.92 | HMDB0241871 | 7-[(1R,2R,3R)-3-Hydroxy-2-[(1E,3S)-3-Hydroxyoct-1-en-1-yl]-5-oxocyclopentyl]heptanoylcarnitine | 2.76 | 46.1 | ▲ |
| 1073.9479 | 9.46 | HMDB0047637 | TG(24:0/20:5(5Z,8Z,11Z,14Z,17Z)/24:1(15Z)) | -0.25 | 39 | ▼ |
| 1040.5124 | 9.48 | HMDB0116058 | CDP-DG(18:2(9Z,11Z)/i-18:0) | -2.55 | 51.9 | ▼ |
| 1118.4687 | 9.48 | HMDB0292986 | CDP-DG(TXB2/a-17:0) | -4.81 | 52 | ▼ |
| 986.5538 | 10.08 | HMDB0289184 | PC(20:5(7Z,9Z,11E,13E,17Z)-3OH(5,6,15)/DiMe(13,5)) | 0.79 | 39.6 | ▲ |
| 961.9716 | 10.27 | HMDB0255686 | Nocistatin | -0.21 | 46.3 | ▲ |
| 651.4094 | 10.71 | HMDB0294753 | DG(12:0/20:5(7Z,9Z,11E,13E,17Z)-3OH(5,6,15)/0:0) | -3.25 | 43.4 | ▲ |
| 514.2849 | 12.09 | HMDB0000036 | Taurocholic acid | 0.99 | 50.7 | ▼ |
| 243.1611 | 12.22 | HMDB0002327 | 1,11-Undecanedicarboxylic acid | 3.83 | 49.6 | ▼ |
| 420.0390 | 12.46 | HMDB0304473 | R-4'-phosphopantothenoyl-L-cysteine | 3.98 | 39.7 | ▼ |
| 540.2727 | 12.89 | HMDB0241613 | (5Z,7S,8E,10Z,13Z,15E,17S,19Z)-7,17-Dihydroxydocosa-5,8,10,13,15,19-hexaenoylcarnitine | -1.16 | 42.4 | ▲ |
| 498.2886 | 13.23 | HMDB0000951 | Taurochenodesoxycholic acid | -1.73 | 37.5 | ▼ |
| 892.4905 | 13.31 | HMDB0012448 | PS(22:6(4Z,7Z,10Z,13Z,16Z,19Z)/20:3(8Z,11Z,14Z)) | 0.51 | 37.7 | ▲ |
| 498.2903 | 13.53 | HMDB0000896 | Taurodeoxycholic acid | 1.54 | 45 | ▼ |
| 687.5449 | 13.61 | HMDB0240617 | SM(d16:1/17:0) | 0.39 | 44.5 | ▲ |
| 544.2662 | 13.67 | HMDB0240603 | LysoPS(18:1(9Z)/0:0) | 1.02 | 49.1 | ▼ |
| 588.3311 | 13.72 | HMDB0010395 | LysoPC(20:4(5Z,8Z,11Z,14Z)/0:0) | 0.80 | 50.5 | ▼ |
| 295.2287 | 14.26 | HMDB0004705 | 12,13-DHOME | 2.59 | 42.6 | ▲ |
| 1151.7065 | 14.43 | HMDB0004844 | Ganglioside GM3 (d18:1/16:0) | 0.54 | 45.5 | ▼ |
| 492.9908 | 14.75 | HMDB0252943 | Phosphatidylinositol 4,5-diphosphate | -2.11 | 38.8 | ▼ |
| 568.3623 | 15.01 | HMDB0258493 | 2-Lysophosphatidylcholine | 0.65 | 54.4 | ▼ |
| 636.3503 | 15.01 | HMDB0252831 | Glycyl-prolyl-arginyl-valyl-valyl-glutamic acid | 4.23 | 48.8 | ▼ |
| 585.3525 | 15.01 | HMDB0115691 | PA(8:0/i-18:0) | -2.22 | 55 | ▼ |
| 568.3624 | 15.23 | HMDB0010384 | LysoPC(18:0/0:0) | 0.70 | 55.1 | ▼ |
| 668.2884 | 15.25 | HMDB0012993 | Leukotriene C5 | 4.01 | 37.1 | ▼ |
| 640.2927 | 15.25 | HMDB0013058 | S-(9-deoxy-delta9,12-PGD2)-glutathione | 2.75 | 38.5 | ▼ |
| 482.2958 | 15.42 | HMDB0000722 | Lithocholyltaurine | 2.57 | 42.6 | ▼ |

Table S4: List of identified metabolites between placebo and RA of HPO extracts in ESI^-^ mode.

| m/z | RT (min) | HMDB ID | **Accepted Description** | Score | ppm | Trend |
| --- | --- | --- | --- | --- | --- | --- |
| 955.4815 | 0.36 | HMDB0276788 | PI(PGJ2/18:3(6Z,9Z,12Z)) | 54.2 | -1.11 | ▲ |
| 318.1333 | 0.55 | HMDB0028890 | Histidyllysine | 47.6 | -1.88 | ▼ |
| 421.1586 | 0.60 | HMDB0006029 | N-Acetylglutamine | 41.4 | 2.53 | ▲ |
| 339.1381 | 0.61 | HMDB0245157 | 2-Hydroxyestriol | 44.8 | 3.93 | ▲ |
| 478.2202 | 0.65 | HMDB0010338 | 11-Oxo-androsterone glucuronide | 50.8 | -3.33 | ▲ |
| 321.1311 | 0.65 | HMDB0033106 | N6-Galacturonyl-L-lysine | 50 | 2.30 | ▲ |
| 258.1468 | 0.86 | HMDB0028952 | Lysylhydroxyproline | 45.2 | 3.40 | ▲ |
| 321.1311 | 0.86 | HMDB0033105 | N2-Galacturonyl-L-lysine | 53.2 | 2.34 | ▲ |
| 351.1413 | 0.86 | HMDB0258925 | tetranor-PGFM | 54.3 | -3.70 | ▲ |
| 700.3629 | 0.89 | HMDB0252831 | Glycyl-prolyl-arginyl-valyl-valyl-glutamic acid | 51 | -1.02 | ▲ |
| 251.1045 | 1.07 | HMDB0010727 | 3-Oxododecanoic acid | 49.9 | -4.45 | ▲ |
| 543.1523 | 1.14 | HMDB0010356 | Estriol 3-sulfate 16-glucuronide | 47.3 | -3.37 | ▲ |
| 888.4057 | 1.16 | HMDB0281968 | PS(PGD2/18:4(6Z,9Z,12Z,15Z)) | 49.2 | -1.61 | ▲ |
| 215.0335 | 1.38 | HMDB0249118 | beta-D-galactosyl | 38.9 | 4.30 | ▲ |
| 231.1359 | 1.40 | HMDB0029065 | Threonylleucine | 48.2 | 3.89 | ▲ |
| 368.0829 | 1.48 | HMDB0254974 | Myo-inositol glutamate | 40.2 | -1.65 | ▲ |
| 370.0812 | 1.48 | HMDB0029082 | Tryptophyl-Glutamate | 52.2 | 0.31 | ▲ |
| 944.5046 | 1.55 | HMDB0116799 | CL(8:0/8:0/8:0/11:0) | 38.2 | 0.29 | ▲ |
| 357.2159 | 1.60 | HMDB0241206 | Undecanedioylcarnitine | 40.4 | -2.27 | ▲ |
| 322.1414 | 1.63 | HMDB0241685 | 3-Hydroxyhept-4-enoylcarnitine | 40.5 | -4.40 | ▲ |
| 964.5955 | 1.79 | HMDB0288145 | PC(20:5(7Z,9Z,11E,13E,17Z)-3OH(5,6,15)/22:3(10Z,13Z,16Z)) | 38.5 | 3.74 | ▲ |
| 535.2725 | 2.09 | HMDB0028734 | Asparaginylisoleucine | 43.2 | -1.67 | ▲ |
| 391.2353 | 2.49 | HMDB0242050 | N-Myristoyl Glutamine | 42.9 | -4.43 | ▲ |
| 409.2193 | 2.57 | HMDB0012109 | 5,6-Dihydroxyprostaglandin F1a | 47 | -3.70 | ▲ |
| 997.5394 | 2.62 | HMDB0277849 | PI(22:3(10Z,13Z,16Z)/18:1(12Z)-2OH(9,10)) | 48.4 | -3.22 | ▲ |
| 809.4997 | 2.67 | HMDB0290438 | SM(d17:2(4E,8Z)/22:6(5Z,7Z,10Z,13Z,16Z,19Z)-OH(4)) | 55 | -1.20 | ▲ |
| 221.0940 | 2.75 | HMDB0028995 | Phenylalanylglycine | 51.1 | 3.85 | ▲ |
| 1076.3639 | 2.77 | HMDB0255943 | Oleoyl coenzyme A | 42.4 | 4.99 | ▲ |
| 389.1963 | 2.82 | HMDB0001908 | 19-Hydroxy-PGE2 | 49.1 | 4.62 | ▲ |
| 971.5520 | 2.89 | HMDB0277839 | PI(22:3(10Z,13Z,16Z)/5-iso PGF2VI) | 49.6 | 1.83 | ▲ |
| 658.3363 | 2.93 | HMDB0288899 | PC(PGF1alpha/2:0) | 50.4 | 3.92 | ▲ |
| 701.3424 | 2.96 | HMDB0262739 | PA(10:0/PGF1alpha) | 50.5 | -2.10 | ▲ |
| 868.4521 | 2.96 | HMDB0284382 | PE(22:6(4Z,7Z,10Z,13Z,16Z,19Z)/5-iso PGF2VI) | 53.3 | -1.89 | ▲ |
| 836.3882 | 3.09 | HMDB0116068 | CDP-DG(a-13:0/i-12:0) | 54.8 | 1.56 | ▲ |
| 880.5340 | 3.13 | HMDB0284054 | PE(22:4(7Z,10Z,13Z,16Z)/6 keto-PGF1alpha) | 52.8 | -0.63 | ▲ |
| 286.1780 | 3.14 | HMDB0248583 | Arginine ornithine | 46.4 | 3.70 | ▲ |
| 783.3985 | 3.14 | HMDB0264130 | PA(PGJ2/18:3(6Z,9Z,12Z)) | 52.6 | -3.22 | ▲ |
| 595.3031 | 3.21 | HMDB0266620 | PA(PGJ2/8:0) | 48.8 | -1.69 | ▲ |
| 735.4399 | 3.32 | HMDB0263562 | PA(18:3(10,12,15)-OH(9)/17:0) | 50.1 | 3.63 | ▲ |
| 507.2324 | 3.47 | HMDB0258743 | Taurodehydrocholic acid | 37.3 | 3.44 | ▲ |
| 426.2358 | 3.79 | HMDB0259877 | Valyl-prolyl-glycyl-valyl-glycine | 48.1 | -0.07 | ▲ |
| 948.4420 | 3.88 | HMDB0292828 | CDP-DG(18:3(10,12,15)-OH(9)/a-13:0) | 53.1 | 2.84 | ▲ |
| 587.2691 | 3.89 | HMDB0010320 | Cortolone-3-glucuronide | 51.4 | -3.31 | ▲ |
| 971.5515 | 3.94 | HMDB0277840 | PI(5-iso PGF2VI/22:3(10Z,13Z,16Z)) | 52.7 | 1.24 | ▲ |
| 717.3957 | 4.08 | HMDB0262820 | PA(12:0/20:4(8Z,11Z,14Z,17Z)-2OH(5S,6R)) | 54.5 | -4.00 | ▲ |
| 961.5177 | 4.08 | HMDB0276423 | PI(22:6(4Z,7Z,10Z,13E,15E,19Z)-OH(17)/18:0) | 53.5 | -4.03 | ▲ |
| 756.4257 | 4.11 | HMDB0270563 | PG(PGJ2/a-13:0) | 48 | 3.73 | ▲ |
| 654.4146 | 4.13 | HMDB0008822 | PE(14:0/14:1(9Z)) | 47.3 | 4.76 | ▲ |
| 738.4513 | 4.13 | HMDB0009203 | PE(18:4(6Z,9Z,12Z,15Z)/20:5(5Z,8Z,11Z,14Z,17Z)) | 50.1 | 1.21 | ▲ |
| 748.3447 | 4.21 | HMDB0060168 | beta-Casomorphin (1-6) | 53.5 | -0.01 | ▲ |
| 939.5870 | 4.25 | HMDB0289038 | PC(DiMe(11,5)/6 keto-PGF1alpha) | 53 | 1.92 | ▲ |
| 405.2487 | 4.27 | HMDB0304784 | Lysyl-Glycine | 44.1 | 5.00 | ▲ |
| 932.5185 | 4.27 | HMDB0274644 | PGP(20:5(6E,8Z,11Z,14Z,17Z)-OH(5)/a-21:0) | 55.3 | -1.13 | ▲ |
| 867.4932 | 4.30 | HMDB0285722 | PC(14:1(9Z)/LTE4) | 50.3 | -3.62 | ▲ |
| 1017.4742 | 4.33 | HMDB0278130 | PI(PGE2/22:5(4Z,7Z,10Z,13Z,16Z)) | 52.7 | -0.67 | ▲ |
| 1057.4801 | 4.33 | HMDB0280373 | PIP(22:3(10Z,13Z,16Z)/18:3(9,11,15)-OH(13)) | 44.7 | -2.46 | ▲ |
| 423.2265 | 4.37 | HMDB0252829 | Gly-pro-arg-pro | 41.5 | 4.48 | ▲ |
| 1099.5837 | 4.40 | HMDB0294297 | CDP-DG(i-22:0/20:3(5Z,11Z,14Z)-O(8,9)) | 45.7 | -4.79 | ▲ |
| 757.4809 | 4.42 | HMDB0116668 | PG(i-12:0/i-20:0) | 44.1 | 2.43 | ▲ |
| 1011.6025 | 4.42 | HMDB0277059 | PI(20:0/PGF1alpha) | 53.1 | -0.18 | ▼ |
| 1012.5009 | 4.49 | HMDB0116049 | CDP-DG(18:2(9Z,11Z)/a-17:0) | 49.1 | -3.76 | ▲ |
| 911.4813 | 4.55 | HMDB0269871 | PG(22:6(5Z,8E,10Z,13Z,15E,19Z)-2OH(7S, 17S)/20:3(8Z,11Z,14Z)) | 52.3 | -3.80 | ▲ |
| 957.4499 | 4.65 | HMDB0278601 | PIP(16:1(9Z)/22:6(4Z,7Z,10Z,13E,15E,19Z)-OH(17)) | 39.3 | -3.78 | ▲ |
| 539.3191 | 4.67 | HMDB0032673 | 15-Octadecene-9,11,13-triynoic acid | 45.2 | 4.41 | ▲ |
| 846.4392 | 4.67 | HMDB0280929 | PS(PGD2/14:1(9Z)) | 50.6 | -2.28 | ▲ |
| 789.4482 | 4.69 | HMDB0263755 | PA(18:1(11Z)/20:4(8Z,11Z,14Z,17Z)-2OH(5S,6R)) | 51.2 | 0.46 | ▲ |
| 705.3769 | 4.72 | HMDB0262883 | PA(PGJ2/13:0) | 46.5 | 2.91 | ▲ |
| 974.5027 | 4.72 | HMDB0276062 | PI(16:0/LTE4) | 48.2 | -4.37 | ▲ |
| 516.2736 | 4.77 | HMDB0242441 | Deoxycholylserine | 47 | 0.71 | ▼ |
| 719.4136 | 4.79 | HMDB0267358 | PA(20:3(8Z,11Z,14Z)-2OH(5,6)/i-12:0) | 43 | -0.77 | ▲ |
| 678.2859 | 4.79 | HMDB0013061 | S-(PGA1)-glutathione | 44.7 | 4.13 | ▲ |
| 1038.3911 | 4.86 | HMDB0293327 | CDP-DG(i-12:0/22:6(5Z,8E,10Z,13Z,15E,19Z)-2OH(7S, 17S)) | 47.4 | 0.96 | ▼ |
| 449.2405 | 4.88 | HMDB0062180 | N-Lactoylisoleucine | 46 | 3.20 | ▲ |
| 445.2333 | 4.88 | HMDB0243591 | Tyrosyl-prolyl-leucyl-glycinamide | 53 | -1.80 | ▼ |
| 1140.5901 | 4.89 | HMDB0294340 | CDP-DG(20:3(8Z,11Z,14Z)-2OH(5,6)/i-22:0) | 49.8 | 1.55 | ▲ |
| 459.2470 | 4.89 | HMDB0029128 | Valylhydroxyproline | 49.6 | 2.12 | ▲ |
| 392.2188 | 4.91 | HMDB0003752 | LysoPC(10:0/0:0) | 46.8 | -4.76 | ▲ |
| 803.4844 | 4.91 | HMDB0030407 | Merodesmosine | 45.9 | -4.94 | ▲ |
| 1077.5334 | 4.91 | HMDB0280323 | PIP(22:3(10Z,13Z,16Z)/20:4(7E,9E,11Z,13E)-3OH(5S,6R,15S)) | 50.6 | 1.13 | ▲ |
| 789.4349 | 4.93 | HMDB0265372 | PA(6 keto-PGF1alpha/20:5(5Z,8Z,11Z,14Z,17Z)) | 54.8 | 0.09 | ▲ |
| 1028.4563 | 4.93 | HMDB0276166 | PI(16:1(9Z)/LTE4) | 49.6 | -1.49 | ▼ |
| 362.1369 | 4.96 | HMDB0028874 | Hydroxyprolyl-Tryptophan | 49.6 | 3.61 | ▼ |
| 599.3233 | 4.98 | HMDB0288825 | PC(20:4(5Z,7E,11Z,14Z)-OH(9)/2:0) | 46 | -0.95 | ▲ |
| 392.1849 | 5.08 | HMDB0241247 | 3-Hydroxydodeca-5,7-dienoylcarnitine | 43.2 | 1.11 | ▲ |
| 415.2259 | 5.09 | HMDB0011579 | MG(20:4(8Z,11Z,14Z,17Z)/0:0/0:0) | 47.6 | 0.70 | ▲ |
| 366.1499 | 5.11 | HMDB0253872 | L-Arginine, L-asparaginylglycyl- | 52 | -2.33 | ▲ |
| 713.3818 | 5.13 | HMDB0263112 | PA(14:1(9Z)/18:1(12Z)-2OH(9,10)) | 44.6 | 2.47 | ▲ |
| 912.4231 | 5.20 | HMDB0265378 | PA(LTE4/20:5(5Z,8Z,11Z,14Z,17Z)) | 48.7 | -3.02 | ▲ |
| 933.4522 | 5.21 | HMDB0278482 | PIP(20:5(6E,8Z,11Z,14Z,17Z)-OH(5)/16:0) | 53.3 | -1.46 | ▲ |
| 758.4656 | 5.27 | HMDB0295262 | DG(LTE4/0:0/15:0) | 49.9 | 1.20 | ▲ |
| 427.1724 | 5.30 | HMDB0041578 | 7,8-Dihydro-3b,6a-dihydroxy-alpha-ionol 9-glucoside | 49.9 | -4.14 | ▲ |
| 729.3741 | 5.35 | HMDB0267412 | PA(PGF1alpha/i-12:0) | 50.3 | -1.41 | ▲ |
| 886.3930 | 5.37 | HMDB0282011 | PS(18:4(6Z,9Z,12Z,15Z)/20:5(7Z,9Z,11E,13E,17Z)-3OH(5,6,15)) | 50.6 | 1.85 | ▼ |
| 555.2453 | 5.44 | HMDB0029113 | Tyrosyl-Proline | 47 | -1.32 | ▲ |
| 693.3176 | 5.46 | HMDB0262714 | PA(20:5(7Z,9Z,11E,13E,17Z)-3OH(5,6,15)/10:0) | 52.1 | 0.02 | ▲ |
| 846.4457 | 5.67 | HMDB0009698 | PE(22:6(4Z,7Z,10Z,13Z,16Z,19Z)/20:5(5Z,8Z,11Z,14Z,17Z)) | 49.3 | -2.99 | ▲ |
| 594.2798 | 5.74 | HMDB0240597 | LysoPI(18:2(9Z,12Z)/0:0) | 46.3 | -3.75 | ▲ |
| 698.4178 | 5.79 | HMDB0267556 | PA(20:4(6Z,8E,10E,14Z)-2OH(5S,12R)/i-14:0) | 51.6 | 0.59 | ▲ |
| 790.4532 | 5.79 | HMDB0280888 | PS(18:3(10,12,15)-OH(9)/14:0) | 55.1 | 2.68 | ▲ |
| 571.3448 | 5.84 | HMDB0253397 | Ile-Ile-Ala-Glu-Lys | 50.6 | -2.18 | ▲ |
| 514.2902 | 5.86 | HMDB0256155 | PC(16:1(9E)/0:0) | 45 | -2.70 | ▲ |
| 838.5009 | 5.88 | HMDB0112651 | PS(20:4(5Z,8Z,11Z,14Z)/22:5(7Z,10Z,13Z,16Z,19Z)) | 53.6 | -2.32 | ▲ |
| 1002.5245 | 5.90 | HMDB0291028 | CDP-DG(18:1(12Z)-O(9S,10R)/18:0) | 56.5 | 1.79 | ▲ |
| 737.4561 | 5.93 | HMDB0263530 | PA(18:1(12Z)-O(9S,10R)/17:0) | 47.5 | 4.42 | ▲ |
| 873.4412 | 5.98 | HMDB0282844 | PS(20:5(7Z,9Z,11E,13E,17Z)-3OH(5,6,15)/20:5(5Z,8Z,11Z,14Z,17Z)) | 53.3 | -3.64 | ▲ |
| 1008.4611 | 6.06 | HMDB0279166 | PIP(PGE2/18:2(9Z,12Z)) | 50.7 | -1.72 | ▼ |
| 1053.4595 | 6.08 | HMDB0279270 | PIP(PGE2/18:3(6Z,9Z,12Z)) | 48 | 0.06 | ▲ |
| 1108.5269 | 6.10 | HMDB0292524 | CDP-DG(PGF1alpha/22:5(4Z,7Z,10Z,13Z,16Z)) | 55 | -1.12 | ▲ |
| 271.1671 | 6.13 | HMDB0028722 | Arginylvaline | 50.8 | 4.13 | ▲ |
| 961.4652 | 6.18 | HMDB0002504 | 3-Sulfodeoxycholic acid | 44.9 | -0.76 | ▼ |
| 840.4250 | 6.18 | HMDB0261785 | PE(20:5(7Z,9Z,11E,13E,17Z)-3OH(5,6,15)/18:4(6Z,9Z,12Z,15Z)) | 54.4 | 3.23 | ▲ |
| 757.4093 | 6.22 | HMDB0270589 | PG(18:1(9Z)-O(12,13)/a-13:0) | 52.8 | 4.13 | ▲ |
| 808.4904 | 6.39 | HMDB0285319 | PE(5-iso PGF2VI/P-18:0) | 56.4 | 0.37 | ▲ |
| 627.3712 | 6.44 | HMDB0012789 | 4-Oxo-13-cis-retinoate | 43.3 | 3.37 | ▲ |
| 610.2909 | 6.49 | HMDB0288891 | PC(18:3(10,12,15)-OH(9)/2:0) | 48.7 | -1.37 | ▲ |
| 507.2821 | 6.51 | HMDB0241823 | (9E)-10-Nitrooctadec-9-enoylcarnitine | 46.6 | -4.47 | ▲ |
| 1114.5760 | 6.51 | HMDB0294324 | CDP-DG(PGD2/i-22:0) | 54.4 | 0.80 | ▲ |
| 737.4586 | 6.57 | HMDB0270585 | PG(18:1(12Z)-2OH(9,10)/a-13:0) | 48.6 | -3.35 | ▲ |
| 767.4658 | 6.61 | HMDB0265004 | PA(22:6(5Z,7Z,10Z,13Z,16Z,19Z)-OH(4)/20:3(5Z,8Z,11Z)) | 49.5 | 0.10 | ▲ |
| 586.3093 | 6.69 | HMDB0252757 | Glu-Ile-Leu-Asp-Val | 46.1 | -0.15 | ▲ |
| 217.0184 | 6.69 | HMDB0041785 | Tyrosol 4-sulfate | 40.5 | 3.74 | ▼ |
| 543.2150 | 6.79 | HMDB0041542 | N2-Maltulosylarginine | 42.3 | -1.15 | ▼ |
| 1084.6023 | 6.86 | HMDB0294396 | CDP-DG(20:3(6,8,11)-OH(5)/i-22:0) | 47.1 | 1.25 | ▲ |
| 855.4438 | 6.98 | HMDB0266212 | PA(TXB2/22:6(4Z,7Z,10Z,13Z,16Z,19Z)) | 55.8 | 1.03 | ▲ |
| 285.1828 | 7.17 | HMDB0028712 | Arginylisoleucine | 52.1 | 4.06 | ▲ |
| 234.9748 | 7.29 | HMDB0259720 | uric acid oxalate | 37.8 | 1.31 | ▲ |
| 534.2815 | 7.53 | HMDB0241888 | 7-[(1R,2R,5S)-5-Hydroxy-2-[(1E,3S)-3-Hydroxyoct-1-en-1-yl]-3-oxocyclopentyl]heptanoylcarnitine | 42.8 | -4.68 | ▼ |
| 850.5424 | 7.57 | HMDB0285680 | PC(14:0/PGF1alpha) | 45.1 | -3.41 | ▲ |
| 779.5032 | 7.62 | HMDB0115370 | PA(22:5(4Z,7Z,10Z,13Z,16Z)/22:4(7Z,10Z,13Z,16Z)) | 40.7 | 1.42 | ▲ |
| 469.3398 | 7.68 | HMDB0006461 | Linoelaidylcarnitine | 41.3 | -2.53 | ▼ |
| 870.4886 | 7.69 | HMDB0282441 | PS(20:3(5Z,8Z,11Z)/22:6(4Z,7Z,11E,13Z,15E,19Z)-2OH(10S,17)) | 53.2 | -4.56 | ▲ |
| 429.2513 | 7.71 | HMDB0242070 | N-Eicosapentaenoyl Glutamic acid | 46.4 | -4.20 | ▼ |
| 835.5119 | 7.83 | HMDB0269523 | PG(20:1(11Z)/PGJ2) | 49.4 | -1.38 | ▲ |
| 825.4720 | 7.90 | HMDB0266050 | PA(22:6(4Z,7Z,11E,13Z,15E,19Z)-2OH(10S,17)/22:5(4Z,7Z,10Z,13Z,16Z)) | 50.5 | 0.96 | ▲ |
| 813.4871 | 7.92 | HMDB0264485 | PA(22:6(5Z,7Z,10Z,13Z,16Z,19Z)-OH(4)/19:0) | 50.2 | 3.60 | ▲ |
| 1057.5144 | 7.95 | HMDB0280341 | PIP(22:3(10Z,13Z,16Z)/18:1(9Z)-O(12,13)) | 44.7 | -4.62 | ▼ |
| 900.4921 | 7.95 | HMDB0281931 | PS(18:3(9Z,12Z,15Z)/PGD1) | 51.1 | 4.81 | ▲ |
| 675.2885 | 8.15 | HMDB0001272 | Nicotine glucuronide | 47.5 | 0.24 | ▲ |
| 897.4353 | 8.32 | HMDB0276281 | PI(5-iso PGF2VI/16:2(9Z,12Z)) | 49 | -3.40 | ▲ |
| 908.5453 | 8.44 | HMDB0284591 | PE(5-iso PGF2VI/24:1(15Z)) | 51.1 | 3.22 | ▲ |
| 1092.5575 | 8.78 | HMDB0294017 | CDP-DG(PGF2alpha/i-19:0) | 49.2 | 2.91 | ▲ |
| 783.4629 | 8.85 | HMDB0266189 | PA(22:6(4Z,7Z,10Z,13Z,16Z,19Z)/20:4(5Z,8Z,11Z,13E)-OH(15S)) | 44.8 | 2.89 | ▼ |
| 742.5399 | 8.99 | HMDB0009090 | PE(18:2(9Z,12Z)/18:0) | 49 | 0.95 | ▲ |
| 768.5544 | 9.07 | HMDB0261876 | PE(20:0/18:1(9Z)-O(12,13)) | 47.9 | -0.56 | ▲ |
| 854.5908 | 9.59 | HMDB0289549 | PC(P-18:0/20:4(5E,8Z,12Z,14Z)-OH(11R)) | 52 | -1.04 | ▲ |
| 857.4574 | 9.92 | HMDB0271716 | PG(i-18:0/5-iso PGF2VI) | 44 | -1.69 | ▼ |
| 986.5538 | 10.08 | HMDB0289184 | PC(20:5(7Z,9Z,11E,13E,17Z)-3OH(5,6,15)/DiMe(13,5)) | 49.3 | 0.79 | ▲ |
| 961.9716 | 10.27 | HMDB0255686 | Nocistatin | 47.6 | -0.21 | ▲ |
| 227.1300 | 10.85 | HMDB0000413 | 3-Hydroxydodecanedioic acid | 41.9 | 4.34 | ▼ |
| 245.0134 | 11.12 | HMDB0003332 | 3-Methoxy-4-Hydroxyphenylglycol sulfate | 42.2 | 3.18 | ▼ |
| 512.3003 | 12.89 | HMDB0061691 | 1-Heptadecanoylglycerophosphoethanolamine | 46.8 | 1.95 | ▼ |
| 452.2789 | 13.09 | HMDB0241461 | 7-Hydroxyhexadecanoylcarnitine | 48.9 | 1.18 | ▼ |
| 584.2308 | 13.09 | HMDB0002580 | Taurolithocholic acid 3-sulfate | 45.4 | -4.47 | ▼ |
| 526.2960 | 13.21 | HMDB0242438 | Deoxycholylproline | 51.4 | 4.09 | ▼ |
| 586.3154 | 13.21 | HMDB0010397 | LysoPC(20:5(5Z,8Z,11Z,14Z,17Z)/0:0) | 52.2 | 0.63 | ▼ |
| 1031.6319 | 13.36 | HMDB0243799 | 1-(9Z-Nonadecenoyl)-glycero-3-phosphoethanolamine | 49.3 | 0.00 | ▼ |
| 538.3155 | 13.36 | HMDB0241885 | 7-[(1R,2R,3R,5S)-3,5-Dihydroxy-2-[(3S)-3-hydroxyoctyl]cyclopentyl]heptanoylcarnitine | 55.5 | 0.68 | ▼ |
| 476.2787 | 13.50 | HMDB0240768 | 3-Hydroxylinoleoylcarnitine | 55.6 | 0.64 | ▼ |
| 285.2082 | 13.99 | HMDB0000672 | Hexadecanedioic acid | 39.9 | 3.62 | ▼ |
| 880.6071 | 14.01 | HMDB0284641 | PE(PGD1/24:1(15Z)) | 54.7 | -0.18 | ▲ |
| 715.5760 | 14.01 | HMDB0240620 | SM(d17:1/18:0) | 53.2 | 0.01 | ▲ |
| 775.5971 | 14.01 | HMDB0001348 | SM(d18:1/18:0) | 48.4 | 0.06 | ▲ |
| 271.2284 | 14.14 | HMDB0112191 | 13-Hydroxyhexadecanoic acid | 39 | 1.90 | ▼ |
| 478.2946 | 14.16 | HMDB0241534 | (12E)-9-Hydroxyoctadecenoylcarnitine | 52.2 | 1.26 | ▼ |
| 578.2193 | 14.33 | HMDB0242178 | Glycocholenate sulfate | 43.6 | -0.52 | ▼ |
| 526.3515 | 14.35 | HMDB0243890 | 1-O-Hexadecyl-sn-glycero-3-phosphocholine | 46.6 | 0.06 | ▼ |
| 833.5198 | 14.35 | HMDB0012444 | PS(22:6(4Z,7Z,10Z,13Z,16Z,19Z)/18:0) | 46 | -2.88 | ▼ |
| 1151.7065 | 14.43 | HMDB0004844 | Ganglioside GM3 (d18:1/16:0) | 45.5 | 0.54 | ▼ |
| 462.2997 | 14.62 | HMDB0011129 | LysoPE(0:0/18:0) | 47.4 | 1.51 | ▼ |
| 838.5927 | 14.84 | HMDB0283756 | PE(22:0/20:3(8Z,11Z,14Z)-2OH(5,6)) | 52.6 | -4.73 | ▲ |
| 250.1459 | 14.99 | HMDB0241690 | Hepta-2,4-dienoylcarnitine | 44.6 | 3.98 | ▼ |
| 568.3623 | 15.01 | HMDB0258493 | 2-Lysophosphatidylcholine | 55.5 | 0.65 | ▼ |
| 640.2927 | 15.01 | HMDB0013058 | S-(9-deoxy-delta9,12-PGD2)-glutathione | 39.6 | 2.70 | ▼ |
| 527.4475 | 15.23 | HMDB0040921 | Diepomuricanin A | 38.4 | 0.99 | ▼ |
| 668.2884 | 15.25 | HMDB0012993 | Leukotriene C5 | 37.1 | 4.01 | ▼ |
| 514.2766 | 15.37 | HMDB0241876 | (5Z)-7-[(1R,2R,5S)-5-Hydroxy-2-[(1E,3S,5Z)-3-hydroxyocta-1,5-dien-1-yl]-3-oxocyclopentyl]hept-5-enoylcarnitine | 52.8 | -4.18 | ▲ |
| 554.3832 | 15.52 | HMDB0297255 | DG(8:0/PGF1alpha/0:0) | 49.7 | -0.42 | ▼ |

Table S5: List of identified metabolites between placebo and MOE of HPO extracts in ESI^+^ mode.

| m/z | RT (min) | **HMDB ID** | **Accepted Description** | Score | ppm | Trend |
| --- | --- | --- | --- | --- | --- | --- |
| 301.0793 | 0.41 | HMDB0036576 | 4-Hydroxy-2-pyrrolidinecarboxylic acid | 43.3 | -1.17 | ▼ |
| 486.1661 | 0.43 | HMDB0000099 | L-Cystathionine | 37.5 | -5.83 | ▼ |
| 162.1126 | 0.47 | HMDB0000062 | L-Carnitine | 46.2 | 0.58 | ▼ |
| 317.2184 | 0.48 | HMDB0061635 | 3-hydroxynonanoyl carnitine | 40.5 | -4.06 | ▼ |
| 282.1787 | 0.48 | HMDB0028955 | Lysylleucine | 45.9 | 1.91 | ▼ |
| 354.1388 | 0.48 | HMDB0029110 | Tyrosyl-Lysine | 46.1 | -3.92 | ▼ |
| 84.0445 | 0.55 | HMDB0000719 | L-Homoserine | 42.2 | 1.13 | ▼ |
| 130.0501 | 0.55 | HMDB0000267 | Pyroglutamic acid | 41.6 | 2.07 | ▼ |
| 147.0766 | 0.56 | HMDB0003423 | D-Glutamine | 47.1 | 1.39 | ▼ |
| 804.4585 | 0.56 | HMDB0260861 | PE(22:6(4Z,7Z,10Z,13E,15E,19Z)-OH(17)/15:0) | 47.1 | 1.19 | ▼ |
| 487.2982 | 0.61 | HMDB0000736 | Isobutyryl-L-carnitine | 32.4 | -1.70 | ▼ |
| 251.1087 | 0.67 | HMDB0010315 | 4-Hydroxyandrostenedione glucuronide | 42.5 | 1.22 | ▼ |
| 203.1383 | 1.33 | HMDB0028691 | Alanylleucine | 48.6 | -3.53 | ▼ |
| 185.1286 | 1.79 | HMDB0028900 | Isoleucyl-Alanine | 51.7 | 0.51 | ▼ |
| 157.1337 | 1.79 | HMDB0013287 | Ne,Ne dimethyllysine | 54.8 | 0.64 | ▼ |
| 322.8760 | 1.86 | HMDB0112906 | PS(24:0/22:5(7Z,10Z,13Z,16Z,19Z)) | 39.6 | -4.01 | ▲ |
| 343.1973 | 1.87 | HMDB0241082 | Dec-4-enedioylcarnitine | 45.5 | -4.90 | ▲ |
| 166.0865 | 2.18 | HMDB0000159 | L-Phenylalanine | 50.0 | 1.69 | ▼ |
| 223.1082 | 2.43 | HMDB0028848 | Glycyl-Phenylalanine | 43.2 | 2.19 | ▼ |
| 140.0455 | 2.47 | HMDB0003157 | Guanidinosuccinic acid | 42.9 | 0.10 | ▼ |
| 521.2902 | 2.86 | HMDB0060531 | Desmethylnortriptyline | 41.7 | -5.04 | ▲ |
| 277.1192 | 2.94 | HMDB0029106 | Tyrosylhydroxyproline | 46.7 | 3.17 | ▼ |
| 879.4913 | 2.98 | HMDB0282694 | PS(PGE2/20:4(8Z,11Z,14Z,17Z)) | 53.7 | 2.31 | ▲ |
| 262.1437 | 3.04 | HMDB0260707 | PE(PGJ2/14:1(9Z)) | 45.9 | 4.00 | ▲ |
| 369.2303 | 3.09 | HMDB0289854 | Cer(d16:1/LTE4) | 47.7 | 3.34 | ▼ |
| 413.2677 | 3.21 | HMDB0265783 | PA(PGE2/22:2(13Z,16Z)) | 51.6 | 0.50 | ▲ |
| 279.1731 | 3.21 | HMDB0264505 | PA(PGF1alpha/19:0) | 43.2 | -3.06 | ▲ |
| 1070.6180 | 3.25 | HMDB0011516 | LysoPE(20:3(8Z,11Z,14Z)/0:0) | 44.1 | -0.10 | ▲ |
| 825.5267 | 3.25 | HMDB0009788 | PI(16:0/20:3(8Z-11Z-14Z)) | 44.5 | -1.13 | ▲ |
| 445.2768 | 3.35 | HMDB0245688 | 25-Hydroxyvitamin D3 3-sulfate ester | 39.2 | -0.65 | ▼ |
| 478.1999 | 3.42 | HMDB0002200 | Leukotriene E4 | 41.7 | -5.68 | ▼ |
| 330.1951 | 3.82 | HMDB0266312 | PA(LTE4/24:0) | 51.1 | 2.49 | ▼ |
| 702.4125 | 3.89 | HMDB0262890 | PA(13:0/20:4(7E,9E,11Z,13E)-3OH(5S,6R,15S)) | 51.9 | 3.16 | ▲ |
| 227.1757 | 3.96 | HMDB0028911 | Isoleucyl-Leucine | 48.8 | 1.23 | ▼ |
| 942.5216 | 4.00 | HMDB0013526 | PGP(18:1(11Z)/20:3(8Z,11Z,14Z)) | 44.0 | -1.83 | ▲ |
| 740.4659 | 4.15 | HMDB0263506 | PA(PGJ2/17:0) | 41.8 | 4.89 | ▲ |
| 157.1336 | 4.16 | HMDB0013287 | Ne-Ne dimethyllysine | 52.2 | 0.37 | ▼ |
| 533.7536 | 4.20 | HMDB0290906 | CDP-DG(TXB2/16:1(9Z)) | 48.7 | -1.18 | ▼ |
| 869.5095 | 4.30 | HMDB0116467 | PGP(20:4(8Z,11Z,14Z,17Z)/20:1(11Z)) | 47.3 | 0.27 | ▲ |
| 400.2265 | 4.40 | HMDB0005096 | N-Arachidonoylglycine | 46.9 | 4.51 | ▼ |
| 211.1438 | 4.44 | HMDB0011175 | Leucylproline | 51.0 | -1.21 | ▼ |
| 344.2231 | 4.59 | HMDB0028703 | Arginylarginine | 42.8 | -3.72 | ▼ |
| 270.1296 | 4.64 | HMDB0028895 | Histidylthreonine | 38.1 | 3.57 | ▼ |
| 448.2302 | 4.65 | HMDB0013587 | PGP(18:3(9Z,12Z,15Z)/20:4(5Z,8Z,11Z,14Z)) | 39.3 | 3.91 | ▼ |
| 573.9670 | 4.69 | HMDB0004926 | Ganglioside GD2 (d18:1/18:0) | 47.8 | -2.32 | ▼ |
| 488.2708 | 4.76 | HMDB0274632 | PGP(18:1(12Z)-2OH(9,10)/a-21:0) | 45.5 | 3.48 | ▼ |
| 901.5566 | 4.79 | HMDB0281515 | PS(PGE1/18:1(11Z)) | 49.0 | 1.97 | ▲ |
| 132.1021 | 4.83 | HMDB0060650 | N-(2-Hydroxyethyl)-morpholine | 47.8 | 1.58 | ▼ |
| 120.0807 | 4.88 | HMDB0246265 | 3H-Dopamine | 51.2 | -0.46 | ▼ |
| 171.1132 | 4.88 | HMDB0000716 | L-Pipecolic acid | 46.5 | 3.42 | ▼ |
| 143.1178 | 4.88 | HMDB0240689 | N2-Methyl-L-lysine | 47.5 | -0.30 | ▼ |
| 818.4346 | 5.00 | HMDB0252655 | GD2 Ganglioside | 36.5 | -4.60 | ▲ |
| 530.7892 | 5.10 | HMDB0248579 | Arg-Pro-Pro-Gly-Phe-Ser-Pro-Phe-Arg | 48.3 | 2.34 | ▲ |
| 437.2382 | 5.30 | HMDB0265931 | PA(22:6(5Z,8E,10Z,13Z,15E,19Z)-2OH(7S, 17S)/22:4(7Z,10Z,13Z,16Z)) | 44.6 | 4.58 | ▼ |
| 429.2713 | 5.32 | HMDB0003581 | Dethiobiotin | 42.3 | 1.35 | ▼ |
| 403.7159 | 5.35 | HMDB0280935 | PS(PGJ2/14:1(9Z)) | 48.6 | 3.93 | ▲ |
| 367.7326 | 5.37 | HMDB0260615 | PE(5-iso PGF2VI/14:0) | 49.1 | -3.08 | ▼ |
| 882.4309 | 5.39 | HMDB0282834 | PS(20:5(5Z,8Z,11Z,14Z,16E)-OH(18R)/20:5(5Z,8Z,11Z,14Z,17Z)) | 55.1 | -1.09 | ▲ |
| 457.2829 | 5.44 | HMDB0274633 | PGP(a-21:0/18:1(12Z)-O(9S,10R)) | 42.6 | 2.18 | ▲ |
| 476.6059 | 5.51 | HMDB0011898 | Ganglioside GM2 (d18:0/18:1(11Z)) | 45.1 | 2.48 | ▲ |
| 573.3937 | 5.55 | HMDB0000053 | Androstenedione | 45.9 | -0.32 | ▲ |
| 425.2603 | 5.55 | HMDB0276191 | PI(18:1(9Z)-O(12,13)/16:1(9Z)) | 46.0 | 1.13 | ▲ |
| 615.4815 | 5.59 | HMDB0289905 | Cer(d17:1/PGJ2) | 37.2 | -1.12 | ▲ |
| 310.2117 | 5.60 | HMDB0297360 | DG(5-iso PGF2VI/a-13:0/0:0) | 42.0 | -3.23 | ▼ |
| 550.8017 | 5.69 | HMDB0294354 | CDP-DG(18:1(12Z)-O(9S,10R)/i-22:0) | 43.8 | 3.43 | ▲ |
| 976.5109 | 5.69 | HMDB0284997 | PE(LTE4/DiMe(9,3)) | 46.2 | 1.77 | ▲ |
| 801.4111 | 5.72 | HMDB0275058 | PGP(20:5(5Z,8Z,10E,14Z,17Z)-OH(12)/i-14:0) | 45.9 | 1.05 | ▲ |
| 812.4459 | 5.83 | HMDB0260802 | PE(15:0/PGE2) | 52.3 | -1.98 | ▲ |
| 1190.6371 | 5.88 | HMDB0293234 | CDP-DG(22:6(4Z,8Z,10Z,13Z,16Z,19Z)-OH(7)/a-25:0) | 55.6 | -1.88 | ▲ |
| 1125.5326 | 5.88 | HMDB0292626 | CDP-DG(PGD1/22:5(7Z,10Z,13Z,16Z,19Z)) | 51.7 | 2.53 | ▲ |
| 393.2137 | 5.88 | HMDB0061042 | N-desethyloxybutynin | 47.2 | -3.40 | ▼ |
| 385.2428 | 5.90 | HMDB0115176 | PA(20:4(8Z,11Z,14Z,17Z)/18:0) | 44.9 | 3.96 | ▲ |
| 1004.5332 | 5.91 | HMDB0116313 | CDP-DG(i-21:0/a-13:0) | 54.4 | -1.61 | ▲ |
| 349.2039 | 5.98 | HMDB0116644 | PG(a-13:0/i-14:0) | 41.4 | -3.26 | ▲ |
| 797.4374 | 6.15 | HMDB0263500 | PA(PGD2/17:0) | 50.6 | 1.09 | ▲ |
| 1022.4796 | 6.35 | HMDB0274507 | PGP(a-17:0/LTE4) | 47.0 | -0.37 | ▲ |
| 850.5236 | 6.42 | HMDB0282558 | PS(PGF1alpha/20:3(8Z,11Z,14Z)) | 53.9 | 0.81 | ▲ |
| 1082.5083 | 6.51 | HMDB0292314 | CDP-DG(PGD1/20:4(8Z,11Z,14Z,17Z)) | 48.8 | -2.79 | ▲ |
| 1116.5903 | 6.53 | HMDB0294330 | CDP-DG(PGJ2/i-22:0) | 52.3 | 0.60 | ▲ |
| 725.4506 | 6.57 | HMDB0265098 | PA(20:5(6E,8Z,11Z,14Z,17Z)-OH(5)/20:3(8Z,11Z,14Z)) | 42.3 | -4.57 | ▲ |
| 739.4706 | 6.59 | HMDB0264593 | PA(22:6(4Z,7Z,10Z,12E,16Z,19Z)-OH(14)/19:2(10Z,13Z)) | 52.4 | 1.11 | ▲ |
| 769.4797 | 6.62 | HMDB0115416 | PA(22:6(4Z,7Z,10Z,13Z,16Z,19Z)/20:4(5Z,8Z,11Z,14Z)) | 42.1 | -0.72 | ▲ |
| 438.7624 | 6.62 | HMDB0284208 | PE(22:5(4Z,7Z,10Z,13Z,16Z)/22:6(4Z,8Z,10Z,13Z,16Z,19Z)-OH(7)) | 47.4 | 2.87 | ▼ |
| 490.7242 | 6.73 | HMDB0116045 | CDP-DG(a-13:0/18:2(9Z,11Z)) | 44.3 | 2.72 | ▼ |
| 483.2392 | 6.74 | HMDB0262581 | PE(LTE4/20:5(5Z,8Z,11Z,14Z,17Z)) | 50.0 | 1.57 | ▼ |
| 455.2452 | 6.74 | HMDB0272659 | PGP(18:1(12Z)-2OH(9,10)/18:1(11Z)) | 46.4 | -3.75 | ▼ |
| 542.2923 | 6.74 | HMDB0280380 | PIP(PGF1alpha/22:3(10Z,13Z,16Z)) | 53.8 | -0.61 | ▲ |
| 1080.5373 | 6.76 | HMDB0280069 | PIP(PGF1alpha/20:4(5Z,8Z,11Z,14Z)) | 55.1 | -4.56 | ▲ |
| 897.4479 | 6.78 | HMDB0275090 | PGP(PGF1alpha/i-14:0) | 52.4 | -2.53 | ▲ |
| 932.4419 | 6.93 | HMDB0292829 | CDP-DG(a-13:0/18:3(9,11,15)-OH(13)) | 43.7 | -1.46 | ▲ |
| 902.9656 | 6.98 | HMDB0011778 | Ganglioside GD1a (d18:0/14:0) | 41.2 | 2.69 | ▲ |
| 132.0812 | 7.00 | HMDB0000466 | 3-Methylindole | 52.1 | 3.13 | ▼ |
| 532.2601 | 7.00 | HMDB0278823 | PIP(PGF1alpha/18:0) | 54.4 | 2.27 | ▲ |
| 981.5937 | 7.02 | HMDB0284926 | PE(DiMe(13,5)/22:6(5Z,8E,10Z,13Z,15E,19Z)-2OH(7S, 17S)) | 53.0 | -0.24 | ▲ |
| 647.3424 | 7.13 | HMDB0288829 | PC(PGD2/2:0) | 46.6 | 4.85 | ▲ |
| 436.2419 | 7.20 | HMDB0269938 | PG(20:4(5Z,8Z,11Z,14Z)/PGJ2) | 46.0 | 3.92 | ▲ |
| 345.1734 | 7.34 | HMDB0278189 | PI(22:5(4Z,7Z,10Z,13Z,16Z)/22:6(4Z,7Z,11E,13Z,15E,19Z)-2OH(10S,17)) | 38.9 | 3.26 | ▼ |
| 895.1541 | 7.42 | HMDB0301613 | 2-ethylpropanedioyl-CoA | 36.3 | -4.93 | ▲ |
| 649.3576 | 7.48 | HMDB0288895 | PC(PGE1/2:0) | 42.3 | 4.12 | ▲ |
| 1150.5761 | 7.57 | HMDB0293136 | CDP-DG(22:6(4Z,7Z,11E,13Z,15E,19Z)-2OH(10S,17)/a-21:0) | 50.1 | 3.99 | ▲ |
| 852.5537 | 7.59 | HMDB0008518 | PC(20:5(5Z,8Z,11Z,14Z,17Z)/22:6(4Z,7Z,10Z,13Z,16Z,19Z)) | 48.4 | -0.10 | ▲ |
| 1006.5031 | 7.61 | HMDB0059791 | Adrenorphin | 47.3 | 1.60 | ▲ |
| 321.1805 | 7.66 | HMDB0247623 | 9,12-Octadecadiynoic acid | 50.9 | 1.45 | ▲ |
| 470.7560 | 7.66 | HMDB0116190 | CDP-DG(i-14:0/a-17:0) | 48.8 | -1.37 | ▲ |
| 1158.6000 | 7.68 | HMDB0294390 | CDP-DG(PGE1/i-22:0) | 49.5 | 1.96 | ▲ |
| 568.1361 | 7.73 | HMDB0028781 | Cysteinyl-Methionine | 37.4 | -0.34 | ▲ |
| 733.2312 | 7.78 | HMDB0012622 | 2-Hydroxyestrone sulfate | 35.3 | -4.74 | ▲ |
| 749.6167 | 7.85 | HMDB0006737 | CE(22:2(13Z,16Z)) | 37.5 | -2.21 | ▲ |
| 823.6251 | 7.85 | HMDB0267310 | PA(20:3(6,8,11)-OH(5)/a-25:0) | 37.7 | 4.70 | ▲ |
| 769.4258 | 7.85 | HMDB0271103 | PG(i-12:0/18:1(12Z)-2OH(9,10)) | 40.8 | 2.70 | ▲ |
| 838.4274 | 7.85 | HMDB0281039 | PS(PGJ2/15:0) | 42.7 | 0.83 | ▲ |
| 592.3232 | 7.92 | HMDB0011845 | Ganglioside GD2 (d18:0/22:1(13Z)) | 42.6 | -1.56 | ▼ |
| 1052.5205 | 8.02 | HMDB0279899 | PIP(PGF2alpha/20:3(8Z,11Z,14Z)) | 38.4 | 0.68 | ▲ |
| 528.3153 | 8.08 | HMDB0296913 | DG(2:0/0:0/6 keto-PGF1alpha) | 52.9 | -2.83 | ▼ |
| 148.0606 | 8.08 | HMDB0006556 | L-4-Hydroxyglutamate semialdehyde | 44.1 | 1.36 | ▼ |
| 553.4998 | 8.18 | HMDB0005830 | 5a-Androstan-3b-ol | 37.1 | 3.42 | ▲ |
| 871.7334 | 8.18 | HMDB0013434 | PC(O-18:1(9Z)/24:0) | 36.9 | -2.23 | ▼ |
| 845.6540 | 8.20 | HMDB0304011 | 1,5-bisdiphosphoinositol-1D-myo-inositol (2,3,4,6)tetrakisphosphate | 37.5 | -1.77 | ▲ |
| 845.4536 | 8.20 | HMDB0269419 | PG(18:3(9Z,12Z,15Z)/PGJ2) | 42.9 | -4.84 | ▲ |
| 132.1020 | 8.25 | HMDB0061716 | N-methylvaline | 51.8 | 0.56 | ▼ |
| 398.7104 | 8.25 | HMDB0112313 | PS(14:1(9Z)/20:5(5Z,8Z,11Z,14Z,17Z)) | 46.4 | -0.08 | ▼ |
| 1007.5173 | 8.32 | HMDB0293730 | CDP-DG(i-16:0/18:1(12Z)-O(9S,10R)) | 40.1 | -3.46 | ▲ |
| 849.3060 | 8.34 | HMDB0060786 | 6-Hydroxymelatonin glucuronide | 40.6 | 2.75 | ▲ |
| 849.4474 | 8.34 | HMDB0270660 | PG(a-15:0/PGD2) | 40.8 | -3.33 | ▲ |
| 744.5935 | 8.36 | HMDB0009483 | PE(22:0/15:0) | 37.9 | 4.43 | ▼ |
| 1151.0121 | 8.37 | HMDB0094240 | DG(a-21:0/0:0/10:0) | 39.7 | -3.42 | ▲ |
| 756.4074 | 8.37 | HMDB0011890 | Ganglioside GM1 (d18:1/14:0) | 43.4 | -0.33 | ▲ |
| 289.1439 | 8.41 | HMDB0006888 | 5b-Cyprinol sulfate | 40.6 | 4.38 | ▼ |
| 318.1902 | 8.41 | HMDB0010355 | Cholestane-3-7-12-25-tetrol-3-glucuronide | 44.3 | -5.71 | ▼ |
| 1169.6448 | 8.46 | HMDB0002577 | Cholic acid glucuronide | 46.0 | -1.55 | ▲ |
| 622.5181 | 8.48 | HMDB0295724 | DG(18:1(9Z)-O(12,13)/17:0/0:0) | 37.9 | 2.32 | ▼ |
| 689.4203 | 8.56 | HMDB0263137 | PA(22:6(4Z,8Z,10Z,13Z,16Z,19Z)-OH(7)/14:1(9Z)) | 50.6 | 3.70 | ▼ |
| 604.3001 | 8.73 | HMDB0060144 | Neocasomorphin (1-5) | 50.4 | 4.03 | ▲ |
| 1094.5688 | 8.80 | HMDB0294017 | CDP-DG(PGF2alpha/i-19:0) | 45.4 | -0.08 | ▲ |
| 727.2465 | 8.83 | HMDB0001013 | Cotinine glucuronide | 37.6 | 4.55 | ▲ |
| 785.4757 | 8.87 | HMDB0266163 | PA(22:5(7Z,10Z,13Z,16Z,19Z)/PGD1) | 55.2 | 0.56 | ▲ |
| 881.6831 | 8.87 | HMDB0008765 | PC(24:0/18:3(9Z,12Z,15Z)) | 38.7 | -0.17 | ▲ |
| 864.4503 | 8.97 | HMDB0274429 | PGP(a-15:0/20:5(5Z,8Z,11Z,14Z,16E)-OH(18R)) | 41.5 | -1.11 | ▲ |
| 772.4773 | 9.10 | HMDB0260849 | PE(20:5(7Z,9Z,11E,13E,17Z)-3OH(5,6,15)/15:0) | 48.1 | 1.82 | ▲ |
| 763.3393 | 9.22 | HMDB0251550 | 2-Hydroxy-4-carboxybutyrylhistidylprolinamide | 38.3 | 3.09 | ▲ |
| 421.7014 | 9.48 | HMDB0116157 | CDP-DG(i-12:0/i-12:0) | 37.8 | -0.93 | ▼ |
| 478.7735 | 9.48 | HMDB0288455 | PC(22:6(5Z,8E,10Z,13Z,15E,19Z)-2OH(7S, 17S)/22:5(7Z,10Z,13Z,16Z,19Z)) | 51.1 | 0.96 | ▼ |
| 492.2467 | 9.48 | HMDB0277308 | PI(PGJ2/20:3(5Z,8Z,11Z)) | 45.0 | -0.65 | ▼ |
| 1057.4686 | 9.48 | HMDB0280101 | PIP(PGE2/20:4(8Z,11Z,14Z,17Z)) | 51.8 | 2.35 | ▼ |
| 902.4715 | 9.50 | HMDB0280936 | PS(14:1(9Z)/LTE4) | 42.3 | 3.16 | ▼ |
| 1014.5059 | 9.59 | HMDB0278752 | PIP(PGE2/18:0) | 43.6 | -1.73 | ▲ |
| 748.4326 | 9.66 | HMDB0008840 | PE(14:0/20:5(5Z,8Z,11Z,14Z,17Z)) | 46.8 | 1.68 | ▲ |
| 1108.5886 | 9.78 | HMDB0291796 | CDP-DG(PGF1alpha/20:1(11Z)) | 44.4 | 3.61 | ▼ |
| 1060.5458 | 9.85 | HMDB0291781 | CDP-DG(20:1(11Z)/22:6(4Z,7Z,10Z,13E,15E,19Z)-OH(17)) | 43.6 | 3.16 | ▼ |
| 883.6971 | 9.92 | HMDB0253282 | hydroxyoctadecenoylcarnitine | 41.1 | -1.11 | ▲ |
| 1085.5637 | 10.32 | HMDB0291183 | CDP-DG(18:1(9Z)/20:4(5Z,8Z,11Z,14Z)-OH(20)) | 45.9 | 4.78 | ▲ |
| 901.5010 | 10.50 | HMDB0013483 | PGP(16:0/22:4(7Z,10Z,13Z,16Z)) | 48.1 | 4.97 | ▲ |
| 749.6037 | 12.28 | HMDB0056099 | DG(22:0/0:0/20:2n6) | 57.0 | 0.98 | ▲ |
| 978.5736 | 12.68 | HMDB0287590 | PC(LTE4/20:4(8Z,11Z,14Z,17Z)) | 38.2 | 0.93 | ▲ |
| 227.0835 | 12.89 | HMDB0240625 | 5alpha-Androstan-3beta,17alpha-diol disulfate | 48.3 | -3.14 | ▼ |
| 434.1858 | 12.91 | HMDB0259129 | Trh-gly | 37.1 | -3.53 | ▼ |
| 819.5242 | 13.60 | HMDB0290292 | SM(d16:1/PGF1alpha) | 47.2 | 0.95 | ▲ |
| 570.7970 | 13.72 | HMDB0294372 | CDP-DG(20:4(8Z,11Z,14Z,17Z)-2OH(5S,6R)/i-22:0) | 49.2 | -0.39 | ▼ |
| 502.2943 | 13.75 | HMDB0242412 | Deoxycholylalanine | 46.6 | 3.02 | ▼ |
| 524.3015 | 13.91 | HMDB0274706 | PGP(PGE2/a-25:0) | 39.5 | 4.77 | ▼ |
| 496.3406 | 14.11 | HMDB0010382 | LysoPC(16:0/0:0) | 42.4 | 1.71 | ▼ |
| 289.6419 | 14.36 | HMDB0296925 | DG(2:0/0:0/LTE4) | 44.1 | 1.31 | ▼ |
| 522.3563 | 14.36 | HMDB0010385 | LysoPC(18:1(11Z)/0:0) | 41.2 | 1.65 | ▼ |
| 801.6762 | 14.86 | HMDB0003550 | Calcidiol | 48.7 | 0.89 | ▼ |
| 504.5136 | 14.94 | HMDB0011760 | Cer(d18:0/16:0) | 40.0 | -0.51 | ▼ |
| 289.1485 | 15.01 | HMDB0013130 | Glutarylcarnitine | 39.4 | 0.28 | ▼ |
| 397.3179 | 15.01 | HMDB0042069 | TG(14:0/14:0/16:1(9Z)) | 34.6 | -0.99 | ▼ |
| 524.3717 | 15.23 | HMDB0010384 | LysoPC(18:0/0:0) | 45.9 | 1.21 | ▼ |

Table S6: List of identified metabolites between placebo and RA of HPO extracts in ESI^+^ mode.

| m/z | RT (min) | HMDB ID | Accepted Description | Score | ppm | Trend |
| --- | --- | --- | --- | --- | --- | --- |
| 237.8057 | 0.36 | HMDB0115038 | PA(18:4(6Z,9Z,12Z,15Z)/18:4(6Z,9Z,12Z,15Z)) | 37.9 | 4.11 | ▲ |
| 306.5125 | 0.36 | HMDB0116529 | PGP(a-13:0/i-24:0) | 37 | -2.87 | ▲ |
| 118.0862 | 0.58 | HMDB0003355 | 5-Aminopentanoic acid | 43.9 | -0.09 | ▼ |
| 890.4537 | 0.58 | HMDB0282628 | PS(20:5(5Z,8Z,11Z,14Z,16E)-OH(18)/20:4(5Z,8Z,11Z,14Z)) | 52.8 | -2.12 | ▲ |
| 335.2202 | 0.98 | HMDB0297569 | DG(a-15:0/0:0/5-iso PGF2VI) | 44.5 | 3.02 | ▲ |
| 325.1672 | 1.67 | HMDB0273921 | PGP(20:5(7Z,9Z,11E,13E,17Z)-3OH(5,6,15)/22:4(7Z,10Z,13Z,16Z)) | 38.3 | 3.32 | ▲ |
| 192.0656 | 1.86 | HMDB0000735 | Hydroxyphenylacetylglycine | 43.1 | 0.50 | ▼ |
| 343.1973 | 1.87 | HMDB0241082 | Dec-4-enedioylcarnitine | 45.5 | -4.90 | ▲ |
| 247.1293 | 2.37 | HMDB0011172 | gamma-Glutamylvaline | 47.1 | 1.90 | ▼ |
| 382.1617 | 2.49 | HMDB0241084 | (2Z)-dec-2-enedioylcarnitine | 43 | -2.89 | ▼ |
| 278.4939 | 2.65 | HMDB0266063 | PA(22:5(4Z,7Z,10Z,13Z,16Z)/20:3(6,8,11)-OH(5)) | 49.3 | -4.23 | ▲ |
| 298.4972 | 2.81 | HMDB0276117 | PI(18:3(10,12,15)-OH(9)/16:0) | 46.3 | 0.79 | ▲ |
| 374.2131 | 2.91 | HMDB0263403 | PA(PGJ2/16:1(9Z)) | 44.8 | -2.43 | ▲ |
| 213.1598 | 2.96 | HMDB0029130 | Valylisoleucine | 54.9 | 0.17 | ▼ |
| 262.1437 | 3.04 | HMDB0260707 | PE(PGJ2/14:1(9Z)) | 45.9 | 4.00 | ▲ |
| 585.3272 | 3.08 | HMDB0004928 | Ganglioside GD2 (d18:1/22:0) | 43.8 | -2.27 | ▲ |
| 369.2303 | 3.09 | HMDB0289854 | Cer(d16:1/LTE4) | 47.7 | 3.34 | ▼ |
| 313.1781 | 3.09 | HMDB0275646 | PGP(i-20:0/PGJ2) | 38.5 | -0.50 | ▲ |
| 392.2438 | 3.14 | HMDB0267097 | PA(a-17:0/PGD1) | 51.5 | 2.80 | ▲ |
| 263.1429 | 3.20 | HMDB0028977 | Methionyl-Leucine | 47.2 | 2.01 | ▼ |
| 413.2677 | 3.21 | HMDB0265959 | PA(PGF1alpha/22:4(7Z,10Z,13Z,16Z)) | 53.1 | 0.50 | ▲ |
| 286.6905 | 3.32 | HMDB0010401 | LysoPC(22:4(7Z,10Z,13Z,16Z)/0:0) | 45.6 | 4.51 | ▲ |
| 562.2511 | 3.32 | HMDB0266510 | PA(PGE2/2:0) | 39.6 | 1.54 | ▲ |
| 222.6632 | 3.35 | HMDB0241943 | N-Stearoyl Histidine | 40.4 | -1.17 | ▲ |
| 362.8534 | 3.70 | HMDB0291731 | CDP-DG(20:1(11Z)/PGJ2) | 44.6 | 2.69 | ▲ |
| 302.1958 | 3.77 | HMDB0241703 | 2-Hydroxy-5-octenoylcarnitine | 45.4 | -1.19 | ▼ |
| 321.1739 | 3.79 | HMDB0266629 | PA(8:0/20:3(8Z,11Z,14Z)-2OH(5,6)) | 39.9 | -3.27 | ▼ |
| 652.2947 | 3.84 | HMDB0258277 | Sialorphin | 51.4 | 3.67 | ▼ |
| 487.2884 | 3.96 | HMDB0277840 | PI(5-iso PGF2VI/22:3(10Z,13Z,16Z)) | 53.9 | 4.77 | ▲ |
| 443.1813 | 4.13 | HMDB0274855 | PGP(i-12:0/20:5(7Z,9Z,11E,13E,17Z)-3OH(5,6,15)) | 37.9 | 1.90 | ▼ |
| 478.7568 | 4.15 | HMDB0265793 | PA(LTE4/22:2(13Z,16Z)) | 39.6 | 0.68 | ▲ |
| 740.4659 | 4.15 | HMDB0263506 | PA(PGJ2/17:0) | 41.8 | 4.89 | ▲ |
| 310.1828 | 4.20 | HMDB0266629 | PA(8:0/20:3(8Z,11Z,14Z)-2OH(5,6)) | 40.2 | -3.76 | ▼ |
| 345.2200 | 4.21 | HMDB0002925 | Dihomo-gamma-linolenic acid | 48 | 3.12 | ▼ |
| 1035.5089 | 4.27 | HMDB0292781 | CDP-DG(a-13:0/20:3(8Z,11Z,14Z)-2OH(5,6)) | 40.8 | 2.29 | ▲ |
| 450.1812 | 4.37 | HMDB0011667 | gamma-Glutamylglycine | 38.5 | -4.46 | ▼ |
| 904.5276 | 4.42 | HMDB0284781 | PE(PGD2/DiMe(11,5)) | 52.6 | -3.90 | ▲ |
| 552.7878 | 4.45 | HMDB0006984 | CDP-DG(18:0/22:3(10Z,13Z,16Z)) | 38.8 | 4.42 | ▲ |
| 340.5082 | 4.45 | HMDB0278341 | PI(22:6(4Z,7Z,10Z,13Z,16Z,19Z)/6 keto-PGF1alpha) | 51.7 | -0.28 | ▲ |
| 360.2151 | 4.62 | HMDB0267688 | PA(20:5(5Z,8Z,10E,14Z,17Z)-OH(12)/i-15:0) | 38.4 | -4.21 | ▼ |
| 942.4886 | 4.62 | HMDB0283373 | PS(22:6(4Z,7Z,10Z,12E,16Z,19Z)-OH(14)/22:5(4Z,7Z,10Z,13Z,16Z)) | 49.8 | 2.01 | ▲ |
| 304.1678 | 4.65 | HMDB0028976 | Methionyl-Isoleucine | 42.3 | -4.31 | ▼ |
| 448.2302 | 4.65 | HMDB0013587 | PGP(18:3(9Z,12Z,15Z)/20:4(5Z,8Z,11Z,14Z)) | 39.3 | 3.91 | ▼ |
| 901.5566 | 4.79 | HMDB0281515 | PS(PGE1/18:1(11Z)) | 49 | 1.97 | ▲ |
| 297.1274 | 4.81 | HMDB0028980 | Methionyl-Phenylalanine | 42.1 | 2.24 | ▼ |
| 419.7740 | 4.83 | HMDB0009639 | PE(22:5(4Z,7Z,10Z,13Z,16Z)/22:6(4Z,7Z,10Z,13Z,16Z,19Z)) | 41.7 | 3.05 | ▼ |
| 143.1178 | 4.88 | HMDB0240689 | N2-Methyl-L-lysine | 47.5 | -0.30 | ▼ |
| 613.4049 | 4.91 | HMDB0298377 | DG(PGJ2/0:0/i-12:0) | 55.9 | -4.28 | ▼ |
| 394.2337 | 4.93 | HMDB0003752 | LysoPC(10:0/0:0) | 39.2 | -3.93 | ▲ |
| 818.4346 | 5.00 | HMDB0252655 | GD2 Ganglioside | 36.5 | -4.60 | ▲ |
| 409.7321 | 5.00 | HMDB0281274 | PS(16:1(9Z)/20:5(5Z,8Z,11Z,14Z,16E)-OH(18R)) | 50.8 | -1.31 | ▲ |
| 309.1970 | 5.10 | HMDB0272111 | PG(i-22:0/PGE2) | 43.3 | -1.45 | ▼ |
| 415.2829 | 5.18 | HMDB0302621 | Dodecadienoic acid | 46.4 | 2.47 | ▼ |
| 391.1991 | 5.34 | HMDB0271094 | PG(5-iso PGF2VI/i-12:0) | 42.3 | 4.83 | ▼ |
| 434.7472 | 5.44 | HMDB0113505 | PE-NMe(20:5(5Z,8Z,11Z,14Z,17Z)/22:6(4Z,7Z,10Z,13Z,16Z,19Z)) | 37.7 | 0.88 | ▲ |
| 460.2608 | 5.44 | HMDB0281144 | PS(16:0/LTE4) | 46.6 | 3.24 | ▼ |
| 615.4815 | 5.59 | HMDB0289905 | Cer(d17:1/PGJ2) | 37.2 | -1.12 | ▲ |
| 310.2117 | 5.60 | HMDB0297360 | DG(5-iso PGF2VI/a-13:0/0:0) | 42 | -3.23 | ▼ |
| 550.8017 | 5.69 | HMDB0294354 | CDP-DG(18:1(12Z)-O(9S,10R)/i-22:0) | 43.8 | 3.43 | ▲ |
| 424.7399 | 5.69 | HMDB0261742 | PE(18:4(6Z,9Z,12Z,15Z)/6 keto-PGF1alpha) | 48.8 | 4.93 | ▲ |
| 976.5109 | 5.69 | HMDB0284997 | PE(LTE4/DiMe(9,3)) | 46.2 | 1.77 | ▲ |
| 318.1814 | 5.74 | HMDB0028940 | Leucyl-Tryptophan | 47.6 | 0.51 | ▼ |
| 385.2428 | 5.90 | HMDB0115176 | PA(20:4(8Z,11Z,14Z,17Z)/18:0) | 44.9 | 3.96 | ▼ |
| 454.7633 | 5.95 | HMDB0281415 | PS(PGF1alpha/18:0) | 42.4 | -4.93 | ▼ |
| 797.4374 | 6.15 | HMDB0263500 | PA(PGD2/17:0) | 50.6 | 1.09 | ▲ |
| 261.1596 | 6.17 | HMDB0253025 | Phenylalanylleucine | 49.9 | -0.70 | ▼ |
| 421.7250 | 6.20 | HMDB0280931 | PS(6 keto-PGF1alpha/14:1(9Z)) | 42.8 | 0.12 | ▲ |
| 861.4458 | 6.32 | HMDB0013576 | PGP(18:3(6Z,9Z,12Z)/22:6(4Z,7Z,10Z,13Z,16Z,19Z)) | 47 | -0.91 | ▼ |
| 785.4369 | 6.35 | HMDB0266730 | PA(TXB2/P-16:0) | 51.4 | 0.51 | ▲ |
| 450.2729 | 6.37 | HMDB0275670 | PGP(i-20:0/18:1(12Z)-O(9S,10R)) | 43.1 | -2.76 | ▼ |
| 644.2938 | 6.41 | HMDB0288867 | PC(20:5(5Z,8Z,10E,14Z,17Z)-OH(12)/2:0) | 46.4 | 0.49 | ▲ |
| 385.2426 | 6.42 | HMDB0114986 | PA(18:3(6Z,9Z,12Z)/20:1(11Z)) | 44.9 | 3.45 | ▼ |
| 1032.6782 | 6.53 | HMDB0004878 | Trihexosylceramide (d18:1/9Z-18:1) | 43.4 | -4.51 | ▼ |
| 509.2725 | 6.59 | HMDB0296912 | DG(6 keto-PGF1alpha/2:0/0:0) | 51.7 | 0.73 | ▲ |
| 739.4706 | 6.59 | HMDB0264593 | PA(22:6(4Z,7Z,10Z,12E,16Z,19Z)-OH(14)/19:2(10Z,13Z)) | 52.4 | 1.11 | ▲ |
| 438.7624 | 6.62 | HMDB0284208 | PE(22:5(4Z,7Z,10Z,13Z,16Z)/22:6(4Z,8Z,10Z,13Z,16Z,19Z)-OH(7)) | 47.4 | 2.87 | ▼ |
| 542.2923 | 6.74 | HMDB0280380 | PIP(PGF1alpha/22:3(10Z,13Z,16Z)) | 53.8 | -0.61 | ▲ |
| 475.2905 | 6.97 | HMDB0242075 | N-Eicosapentaenoyl Lysine | 48.6 | -0.45 | ▼ |
| 1045.5367 | 7.00 | HMDB0001035 | Angiotensin II | 50.6 | 2.60 | ▲ |
| 487.3060 | 7.11 | HMDB0006455 | Arachidonoylcarnitine | 39.3 | 0.31 | ▼ |
| 647.3424 | 7.13 | HMDB0288829 | PC(PGD2/2:0) | 46.6 | 4.85 | ▲ |
| 436.2419 | 7.20 | HMDB0270043 | PG(PGJ2/20:4(8Z,11Z,14Z,17Z)) | 47.9 | 3.92 | ▲ |
| 815.4603 | 7.29 | HMDB0112680 | PS(20:5(5Z,8Z,11Z,14Z,17Z)/14:1(9Z)) | 51.2 | 2.82 | ▼ |
| 345.1734 | 7.34 | HMDB0278189 | PI(22:5(4Z,7Z,10Z,13Z,16Z)/22:6(4Z,7Z,11E,13Z,15E,19Z)-2OH(10S,17)) | 38.9 | 3.26 | ▼ |
| 574.2878 | 7.37 | HMDB0011523 | LysoPE(22:4(7Z,10Z,13Z,16Z)/0:0) | 50.2 | -0.45 | ▼ |
| 479.2862 | 7.44 | HMDB0242012 | N-Docosahexaenoyl Glutamine | 55.3 | -3.99 | ▼ |
| 518.2636 | 7.48 | HMDB0009982 | PIP(18:1(9Z)/18:3(6Z,9Z,12Z)) | 45.7 | 1.77 | ▼ |
| 350.4919 | 7.48 | HMDB0279377 | PIP(18:3(9Z,12Z,15Z)/6 keto-PGF1alpha) | 49.8 | 0.22 | ▼ |
| 334.2375 | 7.49 | HMDB0241325 | 3-Hydroxytrideca-4,6-dienoylcarnitine | 52.9 | -0.41 | ▼ |
| 852.5537 | 7.59 | HMDB0008518 | PC(20:5(5Z,8Z,11Z,14Z,17Z)/22:6(4Z,7Z,10Z,13Z,16Z,19Z)) | 48.4 | -0.10 | ▲ |
| 378.2377 | 7.59 | HMDB0290340 | SM(d16:2(4E,8Z)/18:3(10,12,15)-OH(9)) | 40.9 | -4.17 | ▼ |
| 568.1361 | 7.73 | HMDB0028781 | Cysteinyl-Methionine | 37.4 | -0.34 | ▲ |
| 823.6251 | 7.85 | HMDB0267310 | PA(20:3(6,8,11)-OH(5)/a-25:0) | 37.7 | 4.70 | ▲ |
| 769.4258 | 7.85 | HMDB0271103 | PG(i-12:0/18:1(12Z)-2OH(9,10)) | 40.8 | 2.70 | ▲ |
| 1042.5113 | 7.85 | HMDB0280715 | PIP(22:5(7Z,10Z,13Z,16Z,19Z)/20:4(5Z,8Z,11Z,13E)-OH(15S)) | 37.4 | -2.93 | ▲ |
| 838.4274 | 7.85 | HMDB0281039 | PS(PGJ2/15:0) | 42.7 | 0.83 | ▲ |
| 655.3457 | 7.93 | HMDB0288870 | PC(2:0/22:6(5Z,8E,10Z,13Z,15E,19Z)-2OH(7S, 17S)) | 48 | 1.95 | ▼ |
| 1059.5385 | 7.97 | HMDB0293693 | CDP-DG(20:4(5Z,8Z,10E,14Z)-OH(12S)/i-16:0) | 45.8 | -4.50 | ▼ |
| 1166.7226 | 8.00 | HMDB0117511 | CL(8:0/10:0/12:0/18:2(9Z,11Z)) | 38.7 | -1.58 | ▲ |
| 1012.7454 | 8.00 | HMDB0010553 | TG(22:5(7Z,10Z,13Z,16Z,19Z)/20:5(5Z,8Z,11Z,14Z,17Z)/22:6  (4Z,7Z,10Z,13Z,16Z,19Z)) | 38.8 | -2.52 | ▲ |
| 1009.2580 | 8.05 | HMDB0301076 | 4-Hydroxydodecanedioyl-CoA | 38.4 | -4.96 | ▲ |
| 1153.2912 | 8.05 | HMDB0252809 | Glutamine glutamate aspartate | 36.1 | 1.07 | ▲ |
| 845.4536 | 8.20 | HMDB0269419 | PG(18:3(9Z,12Z,15Z)/PGJ2) | 42.9 | -4.84 | ▲ |
| 849.3060 | 8.34 | HMDB0060786 | 6-Hydroxymelatonin glucuronide | 40.6 | 2.75 | ▲ |
| 1141.1451 | 8.36 | HMDB0013617 | Lipoyl-GMP | 35.5 | -0.02 | ▲ |
| 689.4203 | 8.56 | HMDB0263137 | PA(22:6(4Z,8Z,10Z,13Z,16Z,19Z)-OH(7)/14:1(9Z)) | 50.6 | 3.70 | ▼ |
| 505.3379 | 8.63 | HMDB0240599 | LysoPE(P-18:1(9Z)/0:0) | 46.2 | -4.74 | ▼ |
| 604.3001 | 8.73 | HMDB0060144 | Neocasomorphin (1-5) | 50.4 | 4.03 | ▼ |
| 592.8301 | 8.81 | HMDB0293224 | CDP-DG(22:6(5Z,8E,10Z,13Z,15E,19Z)-2OH(7S, 17S)/a-25:0) | 48.8 | 0.59 | ▼ |
| 785.4757 | 8.87 | HMDB0266163 | PA(22:5(7Z,10Z,13Z,16Z,19Z)/PGD1) | 55.2 | 0.56 | ▼ |
| 881.6831 | 8.87 | HMDB0008765 | PC(24:0/18:3(9Z,12Z,15Z)) | 38.7 | -0.17 | ▲ |
| 1108.5886 | 9.78 | HMDB0291796 | CDP-DG(PGF1alpha/20:1(11Z)) | 44.4 | 3.61 | ▼ |
| 1060.5458 | 9.85 | HMDB0291781 | CDP-DG(20:1(11Z)/22:6(4Z,7Z,10Z,13E,15E,19Z)-OH(17)) | 43.6 | 3.16 | ▼ |
| 493.3370 | 9.85 | HMDB0242370 | Cholylalanine | 44.4 | 1.49 | ▼ |
| 441.2798 | 10.12 | HMDB0271954 | PG(i-20:0/20:4(8Z,11Z,14Z,17Z)-2OH(5S,6R)) | 42 | 1.06 | ▼ |
| 491.2869 | 10.19 | HMDB0277828 | PI(PGJ2/22:3(10Z,13Z,16Z)) | 42.1 | -3.48 | ▼ |
| 1085.5637 | 10.32 | HMDB0291184 | CDP-DG(20:4(5Z,8Z,11Z,14Z)-OH(20)/18:1(9Z)) | 45.9 | 4.78 | ▲ |
| 422.7957 | 10.46 | HMDB0009701 | PE(22:6(4Z,7Z,10Z,13Z,16Z,19Z)/22:2(13Z,16Z)) | 44.6 | -1.05 | ▼ |
| 920.2896 | 10.58 | HMDB0304460 | phenylacetohydroximoyl-glutathione | 36.9 | -1.95 | ▲ |
| 180.1020 | 11.27 | HMDB0094738 | 2-hydroxyoct-1-enoylglycine | 51.1 | 0.60 | ▼ |
| 541.4223 | 11.38 | HMDB0294631 | DG(12:0/0:0/20:4(5Z,8Z,11Z,14Z)-OH(19S)) | 45.9 | -4.96 | ▼ |
| 456.3300 | 11.45 | HMDB0241537 | (9E)-Octadec-9-enedioylcarnitine | 43.2 | -4.41 | ▼ |
| 540.3349 | 11.56 | HMDB0304803 | Leu-Arg-Asn-Arg | 47.4 | -2.93 | ▼ |
| 348.2818 | 11.63 | HMDB0007599 | DG(22:0/18:4(6Z,9Z,12Z,15Z)/0:0) | 49 | -3.24 | ▼ |
| 812.4219 | 11.85 | HMDB0274883 | PGP(i-12:0/20:3(6,8,11)-OH(5)) | 42.4 | -2.05 | ▼ |
| 384.3472 | 11.92 | HMDB0011153 | MG(P-18:0/0:0/0:0) | 50.6 | -0.17 | ▼ |
| 869.5186 | 12.14 | HMDB0284318 | PE(22:5(7Z,10Z,13Z,16Z,19Z)/22:6(4Z,7Z,11E,13Z,15E,19Z)-2OH(10S,17)) | 39.4 | -1.81 | ▼ |
| 384.3111 | 12.28 | HMDB0047902 | TG(14:1(9Z)/14:1(9Z)/18:4(6Z,9Z,12Z,15Z)) | 48.7 | -4.55 | ▼ |
| 399.3292 | 12.60 | HMDB0240785 | 13-Methyltetradecanoylcarnitine | 36.1 | -4.03 | ▼ |
| 357.2645 | 12.63 | HMDB0290277 | SM(d16:1/18:2(10E,12Z)+=O(9)) | 46.9 | -1.59 | ▼ |
| 978.5736 | 12.68 | HMDB0287590 | PC(LTE4/20:4(8Z,11Z,14Z,17Z)) | 38.2 | 0.93 | ▲ |
| 412.3792 | 12.73 | HMDB0255949 | Oleylcarnitine | 55 | 1.60 | ▼ |
| 790.6129 | 12.80 | HMDB0009369 | PE(20:3(8Z,11Z,14Z)/22:0) | 45.6 | 2.40 | ▼ |
| 851.5458 | 12.80 | HMDB0272054 | PG(20:5(7Z,9Z,11E,13E,17Z)-3OH(5,6,15)/i-21:0) | 39.6 | 2.83 | ▼ |
| 568.4558 | 12.80 | HMDB0071469 | TG(8:0/8:0/i-12:0) | 43 | -2.55 | ▼ |
| 227.0835 | 12.89 | HMDB0240625 | 5alpha-Androstan-3beta,17alpha-diol disulfate | 48.3 | -3.14 | ▼ |
| 526.4471 | 13.14 | HMDB0092933 | DG(8:0/18:0/0:0) | 54.9 | 1.02 | ▼ |
| 390.3277 | 13.28 | HMDB0007637 | DG(22:1(13Z)/22:0/0:0) | 41.8 | -2.52 | ▼ |
| 452.2774 | 13.33 | HMDB0242022 | N-Docosahexaenoyl Threonine | 45 | 0.71 | ▼ |
| 494.3252 | 13.36 | HMDB0010383 | LysoPC(16:1(9Z)/0:0) | 44.9 | 2.18 | ▼ |
| 283.2638 | 13.48 | HMDB0061662 | 13-hydroxyoctadecanoic acid | 44.3 | 2.10 | ▼ |
| 340.3217 | 13.48 | HMDB0013208 | 9-Hexadecenoylcholine | 47.9 | 2.06 | ▼ |
| 484.4368 | 13.50 | HMDB0011588 | MG(24:0/0:0/0:0) | 50.4 | 1.81 | ▼ |
| 502.2943 | 13.75 | HMDB0242412 | Deoxycholylalanine | 46.6 | 3.02 | ▼ |
| 528.3148 | 13.75 | HMDB0296932 | DG(TXB2/2:0/0:0) | 40.5 | -3.87 | ▼ |
| 474.3282 | 13.77 | HMDB0241958 | N-Oleoyl Glutamine | 45.6 | -4.90 | ▼ |
| 508.3380 | 13.86 | HMDB0114767 | LysoPA(i-20:0/0:0) | 42 | -3.80 | ▼ |
| 454.4255 | 13.86 | HMDB0062678 | N-hexacosanoylglycine | 48 | -0.04 | ▼ |
| 524.3015 | 13.91 | HMDB0274706 | PGP(PGE2/a-25:0) | 39.5 | 4.77 | ▼ |
| 444.2714 | 13.94 | HMDB0002082 | Bisnorcholic acid | 42.4 | -1.82 | ▼ |
| 566.4413 | 13.96 | HMDB0294523 | DG(10:0/0:0/18:1(9Z)-O(12,13)) | 46.7 | -0.36 | ▼ |
| 771.9663 | 14.11 | HMDB0011874 | Ganglioside GD3 (d18:1/23:0) | 37.9 | -0.02 | ▼ |
| 770.4631 | 14.11 | HMDB0011908 | Ganglioside GM2 (d18:0/26:1(17Z)) | 35.4 | -4.02 | ▼ |
| 522.3565 | 14.19 | HMDB0010385 | LysoPC(18:1(11Z)/0:0) | 51.7 | 2.04 | ▼ |
| 512.4678 | 14.23 | HMDB0240984 | 14-Methyltricosanoylcarnitine | 48.6 | 0.93 | ▼ |
| 311.2948 | 14.23 | HMDB0061665 | 15-hydroxyicosanoic acid | 41.9 | 0.91 | ▼ |
| 448.4147 | 14.23 | HMDB0240947 | 3-Methylhenicosanoylcarnitine | 36.3 | -0.36 | ▼ |
| 470.0480 | 14.23 | HMDB0003335 | IDP | 37.7 | 1.69 | ▼ |
| 481.3126 | 14.35 | HMDB0061691 | 1-Heptadecanoylglycerophosphoethanolamine | 37.1 | -0.22 | ▼ |
| 809.4872 | 14.36 | HMDB0059147 | CL(20:4(5Z,8Z,11Z,14Z)/22:5(7Z,10Z,13Z,16Z,19Z)/22:5  (4Z,7Z,10Z,13Z,16Z)/18:2(9Z,12Z)) | 36.6 | -4.78 | ▼ |
| 289.6419 | 14.36 | HMDB0296925 | DG(2:0/0:0/LTE4) | 44.1 | 1.31 | ▼ |
| 438.2954 | 14.38 | HMDB0297051 | DG(2:0/20:3(6,8,11)-OH(5)/0:0) | 46.7 | -4.97 | ▼ |
| 530.3245 | 14.43 | HMDB0242401 | Chenodeoxycholylvaline | 45.5 | 0.59 | ▼ |
| 496.4360 | 14.56 | HMDB0241627 | (18Z)-Tricos-18-enoylcarnitine | 39.5 | -0.08 | ▼ |
| 731.6038 | 14.86 | HMDB0012088 | SM(d18:0/18:1(11Z)) | 46.1 | -3.26 | ▲ |
| 666.4451 | 15.16 | HMDB0116656 | PG(i-12:0/a-15:0) | 35.6 | 3.08 | ▼ |
| 414.4302 | 15.38 | HMDB0011626 | Dodecanol | 44.2 | -1.01 | ▼ |
| 564.2971 | 15.43 | HMDB0004987 | Aspartyllysine | 39.2 | -3.21 | ▼ |
| 357.2995 | 15.67 | HMDB0011536 | MG(0:0/18:1(11Z)/0:0) | 44 | -1.21 | ▼ |
| 461.3232 | 16.33 | HMDB0002126 | 27-Nor-5b-cholestane-3a,7a,12a,24,25-pentol | 45.4 | -1.36 | ▼ |

Table S7: List of identified metabolites between placebo and AC of HPO extracts in ESI^+^ mode.

| **m/z** | **RT (min)** | **HMDB ID** | **Accepted Description** | **Score** | **ppm** | **Trend** |
| --- | --- | --- | --- | --- | --- | --- |
| 216.1959 | 0.91 | HMDB0013239 | Heptanoylcholine | 42.1 | 0.28 | ▲ |
| 313.1868 | 2.40 | HMDB0294458 | DG(PGD2/10:0/0:0) | 41.7 | -4.00 | ▼ |
| 331.1977 | 2.40 | HMDB0241783 | Nonanedioylcarnitine | 50.1 | -3.64 | ▼ |
| 187.1078 | 2.65 | HMDB0255097 | n-carboxymethyllysine | 53.2 | 0.60 | ▼ |
| 159.1129 | 2.65 | HMDB0003355 | 5-Aminopentanoic acid | 47.8 | 0.67 | ▼ |
| 187.1079 | 3.09 | HMDB0029010 | Prolyl-Alanine | 49.9 | 0.79 | ▼ |
| 392.2438 | 3.14 | HMDB0267097 | PA(a-17:0/PGD1) | 51.5 | 2.80 | ▲ |
| 274.1489 | 3.33 | HMDB0011170 | gamma-Glutamylisoleucine | 44.8 | 0.56 | ▼ |
| 368.5429 | 3.55 | HMDB0117527 | CL(8:0/10:0/i-12:0/13:0) | 41 | -0.69 | ▼ |
| 655.4129 | 3.81 | HMDB0115036 | PA(18:4(6Z,9Z,12Z,15Z)/18:3(6Z,9Z,12Z)) | 49.5 | 1.05 | ▼ |
| 414.7309 | 3.84 | HMDB0009210 | PE(18:4(6Z,9Z,12Z,15Z)/22:6(4Z,7Z,10Z,13Z,16Z,19Z)) | 41.6 | -0.57 | ▼ |
| 600.3730 | 3.89 | HMDB0013022 | Neuromedin N | 50.4 | -4.17 | ▼ |
| 342.6787 | 4.00 | HMDB0297132 | DG(LTE4/8:0/0:0) | 40.3 | -2.54 | ▲ |
| 412.2132 | 4.32 | HMDB0274952 | PGP(20:5(5Z,8Z,11Z,14Z,16E)-OH(18)/i-13:0) | 41 | 4.15 | ▼ |
| 963.5414 | 4.40 | HMDB0241558 | 7,14-Dihydroxyoctadeca-4,8,10,12-tetraenedioylcarnitine | 42.3 | -1.03 | ▼ |
| 942.4886 | 4.62 | HMDB0283373 | PS(22:6(4Z,7Z,10Z,12E,16Z,19Z)-OH(14)/22:5(4Z,7Z,10Z,13Z,16Z)) | 49.8 | 2.01 | ▼ |
| 1016.4701 | 4.65 | HMDB0270461 | PG(LTE4/22:6(4Z,7Z,10Z,13Z,16Z,19Z)) | 50.5 | -1.91 | ▼ |
| 327.0934 | 4.88 | HMDB0029073 | Threonyltyrosine | 44.9 | 2.19 | ▼ |
| 738.4332 | 4.93 | HMDB0263091 | PA(PGJ2/14:1(9Z)) | 52.4 | -1.23 | ▼ |
| 621.3234 | 5.00 | HMDB0288847 | PC(5-iso PGF2VI/2:0) | 38.7 | -0.46 | ▲ |
| 415.2829 | 5.18 | HMDB0302621 | Dodecadienoic acid | 46.4 | 2.47 | ▲ |
| 395.5557 | 5.23 | HMDB0293197 | CDP-DG(a-25:0/20:3(8Z,11Z,14Z)-2OH(5,6)) | 45.2 | 2.24 | ▼ |
| 835.5033 | 5.23 | HMDB0290568 | SM(d18:2(4E,14Z)/PGE2) | 53.6 | 4.41 | ▼ |
| 367.7326 | 5.37 | HMDB0260615 | PE(5-iso PGF2VI/14:0) | 49.1 | -3.08 | ▼ |
| 365.2472 | 5.60 | HMDB0289906 | Cer(d17:1/LTE4) | 43.3 | 3.51 | ▲ |
| 550.8017 | 5.69 | HMDB0294354 | CDP-DG(18:1(12Z)-O(9S,10R)/i-22:0) | 43.8 | 3.43 | ▲ |
| 837.4282 | 5.98 | HMDB0268692 | PG(PGJ2/16:1(9Z)) | 53.6 | -4.09 | ▼ |
| 797.4374 | 6.15 | HMDB0263500 | PA(PGD2/17:0) | 50.6 | 1.09 | ▲ |
| 459.2802 | 6.30 | HMDB0242019 | N-Docosahexaenoyl Methionine | 37 | 0.15 | ▲ |
| 805.4424 | 6.44 | HMDB0265426 | PA(22:6(4Z,7Z,10Z,13E,15E,19Z)-OH(17)/20:5(5Z,8Z,11Z,14Z,17Z)) | 52.8 | 1.23 | ▲ |
| 629.0877 | 6.46 | HMDB0252859 | Glycerylphosphorylinositol | 38.6 | -0.17 | ▲ |
| 1030.4952 | 6.76 | HMDB0292516 | CDP-DG(18:3(10,12,15)-OH(9)/22:5(4Z,7Z,10Z,13Z,16Z)) | 50.1 | -0.15 | ▼ |
| 516.9150 | 6.76 | HMDB0251464 | Diphosphopyridine | 37.6 | 4.44 | ▼ |
| 697.5510 | 6.95 | HMDB0006752 | Dihydroceramide | 39 | 2.74 | ▲ |
| 475.2905 | 6.97 | HMDB0242075 | N-Eicosapentaenoyl Lysine | 48.6 | -0.45 | ▲ |
| 436.2419 | 7.20 | HMDB0270043 | PG(PGJ2/20:4(8Z,11Z,14Z,17Z)) | 47.9 | 3.92 | ▲ |
| 785.4516 | 7.29 | HMDB0114968 | PA(18:2(9Z,12Z)/22:5(4Z,7Z,10Z,13Z,16Z)) | 43.8 | -0.28 | ▲ |
| 1038.5568 | 7.39 | HMDB0278352 | PI(TXB2/22:6(4Z,7Z,10Z,13Z,16Z,19Z)) | 54 | 1.87 | ▼ |
| 569.3543 | 7.62 | HMDB0004891 | Ganglioside GA2 (d18:1/18:0) | 49.9 | 1.68 | ▲ |
| 852.5580 | 7.86 | HMDB0288211 | PC(PGJ2/22:4(7Z,10Z,13Z,16Z)) | 42.6 | 4.77 | ▼ |
| 398.7104 | 8.25 | HMDB0112313 | PS(14:1(9Z)/20:5(5Z,8Z,11Z,14Z,17Z)) | 46.4 | -0.08 | ▼ |
| 818.6086 | 8.41 | HMDB0008056 | PC(18:0/22:5(7Z,10Z,13Z,16Z,19Z)) | 45.7 | 3.34 | ▲ |
| 1056.5603 | 8.57 | HMDB0274612 | PGP(LTE4/a-21:0) | 43.8 | -0.36 | ▲ |
| 871.4929 | 9.10 | HMDB0276199 | PI(20:5(6E,8Z,11Z,14Z,17Z)-OH(5)/16:1(9Z)) | 43.4 | -4.36 | ▲ |
| 625.3635 | 9.31 | HMDB0117902 | CL(8:0/10:0/18:2(9Z,11Z)/18:2(9Z,11Z)) | 39.8 | -3.75 | ▲ |
| 710.4123 | 9.41 | HMDB0263090 | PA(14:1(9Z)/PGJ2) | 37.9 | 0.70 | ▲ |
| 853.5344 | 9.53 | HMDB0260912 | PE(16:0/PGF2alpha) | 52.4 | 3.84 | ▲ |
| 859.5280 | 9.66 | HMDB0285205 | PE(LTE4/P-16:0) | 38.5 | 1.62 | ▲ |
| 398.7778 | 10.68 | HMDB0289458 | PC(PGJ2/P-16:0) | 45.5 | -0.37 | ▲ |
| 919.7102 | 10.89 | HMDB0241937 | N-Stearoyl Arginine | 38.6 | 1.96 | ▼ |
| 891.7031 | 10.97 | HMDB0013456 | PC(O-22:2(13Z,16Z)/22:3(10Z,13Z,16Z)) | 35.4 | -0.98 | ▼ |
| 405.7874 | 11.10 | HMDB0261935 | PE(20:4(5Z,8Z,11Z,14Z)-OH(17)/20:1(11Z)) | 45.1 | 3.82 | ▲ |
| 456.3300 | 11.45 | HMDB0241537 | (9E)-Octadec-9-enedioylcarnitine | 43.2 | -4.41 | ▼ |
| 458.3464 | 11.85 | HMDB0240777 | O-(17-Carboxyheptadecanoyl)carnitine | 38.7 | -2.70 | ▼ |
| 495.2221 | 13.23 | HMDB0304787 | Phenylalanyl-Alanine | 40.6 | 1.46 | ▼ |
| 452.2774 | 13.33 | HMDB0242022 | N-Docosahexaenoyl Threonine | 45 | 0.71 | ▼ |
| 757.5573 | 14.86 | HMDB0008843 | PE(14:0/22:2(13Z,16Z)) | 37.6 | -1.05 | ▼ |

Table S8: List of identified metabolites between placebo and MOE of HPI extracts in ESI^+^ mode.

| **m/z** | **RT (min)** | **Accepted Compound ID** | **Accepted Description** | **Score** | **ppm** | **Trend** |
| --- | --- | --- | --- | --- | --- | --- |
| 304.1835 | 0.73 | HMDB0010330 | Cholesterol glucuronide | 47 | 2.72 | ▲ |
| 529.3783 | 0.73 | HMDB0250171 | Cholesterol glutamate | 47 | 3.96 | ▼ |
| 441.3320 | 0.73 | HMDB0241975 | N-Linoleoyl Glutamine | 49 | -0.76 | ▼ |
| 221.1698 | 0.75 | HMDB0006764 | 17a,20a-Dihydroxycholesterol | 42.4 | -3.69 | ▼ |
| 243.1343 | 1.06 | HMDB0028823 | Glutamylleucine | 53.2 | 1.58 | ▲ |
| 219.1340 | 1.19 | HMDB0028916 | Isoleucyl-Serine | 49.4 | 0.53 | ▲ |
| 229.1190 | 1.91 | HMDB0060747 | 3-O-Methyl-a-methyldopa | 43.2 | 3.60 | ▲ |
| 247.1292 | 2.40 | HMDB0028756 | Aspartyl-Isoleucine | 49.4 | 1.27 | ▲ |
| 217.1549 | 2.81 | HMDB0094801 | 2-octenoylglycine | 44.5 | 1.27 | ▲ |
| 231.1708 | 2.91 | HMDB0029131 | Valylleucine | 51.6 | 1.88 | ▲ |
| 354.1662 | 3.40 | HMDB0241751 | 6-Hydroxynon-7-enoylcarnitine | 50.9 | -4.92 | ▲ |
| 281.1134 | 3.44 | HMDB0000706 | Aspartylphenylalanine | 43 | 0.56 | ▲ |
| 281.1131 | 3.72 | HMDB0003331 | 1-Methyladenosine | 45.6 | 4.50 | ▲ |
| 295.1653 | 3.76 | HMDB0011177 | Phenylalanylproline | 48.5 | 0.30 | ▲ |
| 423.2226 | 3.96 | HMDB0266102 | PA(PGJ2/22:5(7Z,10Z,13Z,16Z,19Z)) | 41.2 | 4.87 | ▲ |
| 514.2808 | 4.00 | HMDB0279526 | PIP(20:0/20:4(8Z,11Z,14Z,17Z)-2OH(5S,6R)) | 44.5 | 2.43 | ▲ |
| 412.7127 | 4.04 | HMDB0263093 | PA(LTE4/14:1(9Z)) | 49.7 | 4.68 | ▲ |
| 454.7385 | 4.15 | HMDB0283533 | PS(PGJ2/22:6(4Z,7Z,10Z,13Z,16Z,19Z)) | 38 | 1.40 | ▲ |
| 362.7308 | 4.16 | HMDB0260661 | PE(18:3(9,11,15)-OH(13)/14:0) | 48.3 | 2.69 | ▲ |
| 420.7223 | 4.18 | HMDB0280882 | PS(22:6(4Z,7Z,10Z,13E,15E,19Z)-OH(17)/14:0) | 41.5 | -3.21 | ▲ |
| 433.2468 | 4.30 | HMDB0269418 | PG(PGF2alpha/18:3(9Z,12Z,15Z)) | 49.4 | 3.07 | ▲ |
| 428.0341 | 4.33 | HMDB0254870 | monophospho-n-acetylneuraminic acid | 38.2 | -3.49 | ▼ |
| 539.2719 | 4.49 | HMDB0280353 | PIP(22:3(10Z,13Z,16Z)/20:5(7Z,9Z,11E,13E,17Z)-3OH(5,6,15)) | 39.6 | 4.96 | ▲ |
| 345.2233 | 4.57 | HMDB0298864 | DG(22:6(5Z,8E,10Z,13Z,15E,19Z)-2OH(7S, 17S)/0:0/i-14:0) | 43.1 | 4.64 | ▲ |
| 496.8145 | 4.57 | HMDB0283700 | PS(PGF1alpha/24:0) | 40.7 | 4.45 | ▲ |
| 280.1909 | 4.67 | HMDB0116762 | PS(20:1(11Z)/20:4(8Z,11Z,14Z,17Z)) | 44.4 | -1.32 | ▼ |
| 823.9599 | 4.69 | HMDB0004858 | Ganglioside GM1 (18:1/22:0) | 48 | 1.26 | ▲ |
| 263.1389 | 4.76 | HMDB0029118 | Tyrosyl-Valine | 49.4 | -0.31 | ▲ |
| 573.9539 | 4.86 | HMDB0001294 | 2-3-Diphosphoglyceric acid | 38.2 | 2.82 | ▼ |
| 336.3794 | 4.89 | HMDB0243947 | 1-Methylspermidine | 37.9 | -4.85 | ▲ |
| 461.2523 | 4.93 | HMDB0276275 | PI(TXB2/16:2(9Z,12Z)) | 46.2 | 0.28 | ▲ |
| 410.1992 | 4.96 | HMDB0001032 | Dehydroepiandrosterone sulfate | 46.1 | -0.87 | ▲ |
| 714.5654 | 5.00 | HMDB0240638 | SM(d18:2(4E,14Z)/16:0) | 36.8 | 2.78 | ▼ |
| 431.2341 | 5.01 | HMDB0116545 | PGP(i-12:0/i-21:0) | 37.5 | -2.51 | ▲ |
| 398.2061 | 5.08 | HMDB0270575 | PG(5-iso PGF2VI/a-13:0) | 44.6 | 2.50 | ▲ |
| 421.2502 | 5.22 | HMDB0010640 | PG(18:1(9Z)/20:4(5Z,8Z,11Z,14Z)) | 43.7 | -4.43 | ▲ |
| 835.5033 | 5.23 | HMDB0113490 | PE-NMe(20:5(5Z,8Z,11Z,14Z,17Z)/18:4(6Z,9Z,12Z,15Z)) | 40.5 | 4.67 | ▲ |
| 610.0734 | 5.27 | HMDB0061132 | Celecoxib glucuronide | 37.8 | 3.47 | ▼ |
| 762.5969 | 5.27 | HMDB0296698 | DG(PGE2/0:0/22:0) | 37.4 | 0.05 | ▼ |
| 215.1275 | 5.35 | HMDB0303770 | (S)-2-Methyl-1-butanol O-beta-D-Glucopyranoside | 53.7 | -1.27 | ▼ |
| 421.2521 | 5.35 | HMDB0116600 | PG(22:6(4Z,7Z,10Z,13Z,16Z,19Z)/18:2(9Z,12Z)) | 50.3 | -2.49 | ▲ |
| 439.2043 | 5.52 | HMDB0247924 | Acetyl-arginyl-glycyl-aspartyl-serinamide | 44.4 | -0.98 | ▲ |
| 751.5507 | 5.57 | HMDB0011567 | MG(18:1(9Z)/0:0/0:0) | 36.7 | 3.13 | ▼ |
| 828.6277 | 5.64 | HMDB0007943 | PC(18:4(6Z-9Z-12Z-15Z)/24:1(15Z)) | 45.6 | 1.33 | ▼ |
| 480.2722 | 5.67 | HMDB0275720 | PGP(i-21:0/20:3(8Z,11Z,14Z)-O(5,6)) | 44.9 | -1.56 | ▲ |
| 350.2070 | 5.93 | HMDB0303977 | (5Z)-(15S)-11-alpha-hydroxy-9,15-dioxoprosta-13-enoate | 47.9 | -4.99 | ▲ |
| 451.7424 | 6.06 | HMDB0283571 | PS(20:5(7Z,9Z,11E,13E,17Z)-3OH(5,6,15)/22:6(4Z,7Z,10Z,13Z,16Z,19Z)) | 44.4 | -4.25 | ▼ |
| 530.3180 | 6.08 | HMDB0028867 | Hydroxyprolyl-Leucine | 46.4 | -0.80 | ▲ |
| 545.2657 | 6.32 | HMDB0279483 | PIP(PGF2alpha/20:0) | 55.8 | -2.03 | ▲ |
| 509.4462 | 6.41 | HMDB0006509 | Nervonyl carnitine | 40.9 | 4.51 | ▲ |
| 525.2722 | 6.42 | HMDB0280212 | PIP(PGJ2/22:2(13Z,16Z)) | 42.9 | 0.87 | ▲ |
| 494.2605 | 6.62 | HMDB0277100 | PI(PGJ2/20:1(11Z)) | 44.2 | -4.58 | ▲ |
| 890.8132 | 6.73 | HMDB0012122 | (Mannosyl)6-(N-acetylglucosaminyl)2-diphosphodolichol | 46.7 | -0.13 | ▼ |
| 445.3018 | 6.81 | HMDB0241510 | 5-Hydroxyhexadecanedioylcarnitine | 44.9 | -3.49 | ▲ |
| 660.5543 | 6.91 | HMDB0093016 | DG(10:0/0:0/i-24:0) | 38 | 0.93 | ▲ |
| 951.9516 | 6.93 | HMDB0252614 | gamma-Endorphin | 42.9 | 3.48 | ▲ |
| 1011.2803 | 7.06 | HMDB0003712 | (2E)-Dodecenoyl-CoA | 37 | -2.18 | ▼ |
| 726.1924 | 7.08 | HMDB0252788 | Glucose pyruvate lactate | 39.2 | -1.58 | ▲ |
| 378.2370 | 7.59 | HMDB0000903 | Tetrahydrocortisone | 37.4 | 1.38 | ▼ |
| 521.7419 | 7.81 | HMDB0292897 | CDP-DG(a-15:0/18:1(12Z)-2OH(9,10)) | 37.5 | 1.21 | ▼ |
| 517.9854 | 7.81 | HMDB0001541 | Guanosine hexaphosphate adenosine | 37.1 | -1.45 | ▼ |
| 508.7644 | 7.81 | HMDB0276894 | PI(LTE4/18:3(9Z,12Z,15Z)) | 42.9 | 4.91 | ▼ |
| 697.1868 | 8.18 | HMDB0010329 | Dopamine glucuronide | 37.2 | 2.36 | ▼ |
| 871.7332 | 8.18 | HMDB0008785 | PC(24:0/P-18:0) | 36.5 | -2.41 | ▼ |
| 928.4842 | 8.25 | HMDB0274110 | PGP(22:5(7Z,10Z,13Z,16Z,19Z)/18:2(9Z,11E)+=O(13)) | 44 | 1.73 | ▲ |
| 966.2809 | 8.43 | HMDB0300956 | Undec-3-enoyl-CoA | 38.2 | -3.84 | ▼ |
| 677.1402 | 8.61 | HMDB0244558 | N-(p-Toluenesulfonyl)-L-phenylalanine | 36.3 | 2.15 | ▼ |
| 532.2807 | 8.69 | HMDB0280358 | PIP(20:4(8Z,11Z,14Z,17Z)-2OH(5S,6R)/22:3(10Z,13Z,16Z)) | 46.3 | 2.04 | ▲ |
| 656.5081 | 8.87 | HMDB0299230 | DG(i-16:0/0:0/5-iso PGF2VI) | 36.3 | -2.40 | ▼ |
| 881.6840 | 8.87 | HMDB0008158 | PC(18:2(9Z,12Z)/24:1(15Z)) | 38.7 | 0.83 | ▼ |
| 705.3512 | 8.87 | HMDB0288886 | PC(2:0/22:6(4Z,7Z,11E,13Z,15E,19Z)-2OH(10S,17)) | 45.2 | 4.01 | ▼ |
| 821.4384 | 8.87 | HMDB0272485 | PGP(18:3(10,12,15)-OH(9)/16:1(9Z)) | 46.6 | 2.38 | ▼ |
| 983.5114 | 9.05 | HMDB0293524 | CDP-DG(i-14:0/18:1(9Z)-O(12,13)) | 41.7 | -0.31 | ▼ |
| 1044.5269 | 9.12 | HMDB0280499 | PIP(22:4(7Z,10Z,13Z,16Z)/20:4(5Z,8Z,11Z,14Z)-OH(19S)) | 45.7 | -2.99 | ▲ |
| 918.4550 | 9.44 | HMDB0272465 | PGP(22:6(5Z,8E,10Z,13Z,15E,19Z)-2OH(7S, 17S)/16:1(9Z)) | 37.4 | -7.62 | ▼ |
| 902.4628 | 9.48 | HMDB0272876 | PGP(20:5(5Z,8Z,10E,14Z,17Z)-OH(12)/18:2(9Z,11Z)) | 50 | -4.67 | ▼ |
| 338.5070 | 9.48 | HMDB0275758 | PGP(i-21:0/20:4(7E,9E,11Z,13E)-3OH(5S,6R,15S)) | 37.8 | -3.96 | ▼ |
| 1100.7856 | 9.71 | HMDB0004886 | Trihexosylceramide (d18:1/24:0) | 41.8 | 3.21 | ▼ |
| 620.2136 | 10.10 | HMDB0002091 | Isovalerylglucuronide | 37.9 | -4.51 | ▼ |
| 899.4883 | 10.12 | HMDB0282841 | PS(20:5(5Z,8Z,11Z,14Z,17Z)/22:6(5Z,8E,10Z,13Z,15E,19Z)-2OH(7S, 17S)) | 38.6 | -2.78 | ▼ |
| 769.6116 | 10.38 | HMDB0007499 | DG(20:3(8Z,11Z,14Z)/24:0/0:0) | 36.5 | 1.24 | ▼ |
| 962.0133 | 10.38 | HMDB0001380 | Diguanosine pentaphosphate | 38 | -2.86 | ▼ |
| 945.5081 | 10.48 | HMDB0002586 | Chenodeoxycholic acid 3-sulfate | 41.6 | 2.03 | ▼ |
| 939.0881 | 10.51 | HMDB0059613 | 7-Methylguanosine 5'-diphosphate | 36.4 | 3.55 | ▼ |
| 864.4496 | 10.53 | HMDB0275159 | PGP(i-15:0/20:5(5Z,8Z,11Z,14Z,16E)-OH(18)) | 39.9 | -2.04 | ▼ |
| 987.5118 | 10.53 | HMDB0275882 | PGP(i-22:0/20:5(5Z,8Z,11Z,14Z,16E)-OH(18R)) | 41 | -0.71 | ▼ |
| 906.7664 | 10.58 | HMDB0043752 | TG(15:0/22:5(7Z,10Z,13Z,16Z,19Z)/18:1(9Z)) | 39.6 | 3.17 | ▼ |
| 412.3788 | 12.72 | HMDB0255949 | Oleylcarnitine | 51.8 | 0.78 | ▼ |
| 788.6072 | 12.79 | HMDB0296790 | DG(20:5(7Z,9Z,11E,13E,17Z)-3OH(5,6,15)/0:0/22:0) | 49.9 | 4.97 | ▼ |
| 288.2536 | 12.97 | HMDB0030972 | 8-Oxohexadecanoic acid | 51.1 | 0.99 | ▼ |
| 402.3580 | 13.03 | HMDB0062341 | N-Stearoyl GABA | 49.4 | 0.52 | ▼ |
| 494.3246 | 13.36 | HMDB0012458 | 7alpha-Hydroxy-3-oxo-4-cholestenoate | 47.7 | 1.14 | ▼ |
| 484.4362 | 13.48 | HMDB0011558 | MG(0:0/24:0/0:0) | 53.2 | 0.44 | ▼ |
| 357.2797 | 13.56 | HMDB0000308 | 3b-Hydroxy-5-cholenoic acid | 46.6 | 2.46 | ▼ |
| 478.2982 | 13.67 | HMDB0242415 | Deoxycholylcysteine | 43.1 | -0.78 | ▼ |
| 544.3405 | 13.72 | HMDB0010396 | LysoPC(20:4(8Z,11Z,14Z,17Z)/0:0) | 49.1 | 1.28 | ▼ |
| 430.3892 | 13.79 | HMDB0241944 | N-Stearoyl Isoleucine | 55 | 0.29 | ▼ |
| 540.4263 | 13.80 | HMDB0241806 | 13-(3-Methyl-5-pentylfuran-2-yl)tridecanoylcarnitine | 43.8 | 0.92 | ▼ |
| 454.2935 | 14.07 | HMDB0000328 | 12-Ketodeoxycholic acid | 52.1 | 1.95 | ▼ |
| 267.6475 | 14.11 | HMDB0242372 | Cholylcysteine | 52.7 | 3.42 | ▼ |
| 496.3404 | 14.11 | HMDB0010382 | LysoPC(16:0/0:0) | 51.4 | 1.31 | ▼ |
| 368.3526 | 14.21 | HMDB0240596 | Oleoylcholine | 52.5 | 0.93 | ▼ |
| 442.2589 | 14.35 | HMDB0241355 | 3,10-Dihydroxytetradecanoylcarnitine | 42.3 | 5.85 | ▼ |
| 478.2358 | 14.36 | HMDB0242076 | N-Eicosapentaenoyl Methionine | 42.6 | -0.96 | ▼ |
| 140.0686 | 14.38 | HMDB0246748 | 5-Aminoimidazole-4-carboxyamide | 42.7 | -4.88 | ▼ |
| 522.3561 | 14.38 | HMDB0256159 | PC(18:1(6Z)/0:0) | 48.9 | 1.25 | ▼ |
| 572.3715 | 14.47 | HMDB0010344 | Vitamin D2 3-glucuronide | 46.5 | 1.31 | ▼ |
| 508.3754 | 14.60 | HMDB0013122 | LysoPC(P-18:0/0:0) | 41.3 | -1.49 | ▼ |
| 285.1694 | 14.79 | HMDB0059661 | QH(2) | 46 | -1.08 | ▼ |
| 482.3247 | 15.19 | HMDB0241526 | 6-Hydroxyoctadecanoylcarnitine | 47.4 | 1.16 | ▼ |
| 281.6631 | 15.23 | HMDB0242382 | Cholylmethionine | 50.5 | 3.08 | ▼ |
| 524.3717 | 15.23 | HMDB0010384 | LysoPC(18:0/0:0) | 50.6 | 1.25 | ▼ |
| 585.2262 | 15.45 | HMDB0010357 | Tetrahydroaldosterone-3-glucuronide | 44.1 | -3.83 | ▼ |
| 413.3243 | 16.37 | HMDB0011545 | MG(0:0/20:3(11Z,14Z,17Z)/0:0) | 46 | -4.80 | ▼ |
| 481.3140 | 16.38 | HMDB0242065 | N-Eicosapentaenoyl Arginine | 54.6 | -1.92 | ▼ |

Table S9: List of identified metabolites between placebo and RA of HPI extracts in ESI^+^ mode.

| **m/z** | **RT (min)** | **Accepted Compound ID** | **Accepted Description** | **Score** | **ppm** | **Trend** |
| --- | --- | --- | --- | --- | --- | --- |
| 238.1626 | 0.38 | HMDB0062386 | 4-carboxy-5-cholesta-8-en-3-ol | 42.7 | 4.95 | ▲ |
| 359.2403 | 0.56 | HMDB0012983 | Kinetensin 1-3 | 44.3 | 0.48 | ▼ |
| 298.4967 | 2.84 | HMDB0276194 | PI(16:1(9Z)/20:5(5Z,8Z,11Z,14Z,16E)-OH(18)) | 48.5 | -3.63 | ▲ |
| 306.5159 | 3.33 | HMDB0276499 | PI(18:1(12Z)-2OH(9,10)/18:1(11Z)) | 41.6 | -3.42 | ▲ |
| 335.1850 | 4.37 | HMDB0247371 | H-Tyr-gly-gly-phe-leu-arg-arg-ile-OH | 46.1 | -4.37 | ▲ |
| 328.2312 | 4.72 | HMDB0240594 | N-Palmitoyltaurine | 47 | 2.09 | ▲ |
| 505.7425 | 6.08 | HMDB0292770 | CDP-DG(PGF2alpha/a-13:0) | 42.4 | 2.60 | ▼ |
| 210.1103 | 6.25 | HMDB0013010 | N-Heptanoylglycine | 50.2 | 1.16 | ▲ |
| 592.3891 | 6.62 | HMDB0304796 | Arg-Thr-Lys-Arg | 44.5 | 0.36 | ▲ |
| 705.3751 | 6.86 | HMDB0116518 | PGP(a-13:0/i-12:0) | 40.1 | 1.81 | ▼ |
| 359.1864 | 7.22 | HMDB0000332 | 18-Oxocortisol | 44.8 | 2.80 | ▲ |
| 696.4369 | 7.22 | HMDB0263229 | PA(20:5(6E,8Z,11Z,14Z,17Z)-OH(5)/15:0) | 48.3 | 1.16 | ▲ |
| 379.2484 | 7.39 | HMDB0290328 | SM(d16:2(4E,8Z)/20:5(5Z,8Z,10E,14Z,17Z)-OH(12)) | 41.7 | 0.69 | ▲ |
| 784.4890 | 7.56 | HMDB0115221 | PA(20:5(5Z,8Z,11Z,14Z,17Z)/22:6(4Z,7Z,10Z,13Z,16Z,19Z)) | 50.9 | -2.89 | ▲ |
| 401.2616 | 7.56 | HMDB0290572 | SM(d18:2(4E,14Z)/PGJ2) | 43.5 | 0.73 | ▲ |
| 425.7514 | 7.69 | HMDB0261374 | PE(18:1(9Z)/22:6(5Z,7Z,10Z,13Z,16Z,19Z)-OH(4)) | 40.7 | -1.70 | ▲ |
| 423.2742 | 7.69 | HMDB0290519 | SM(d18:1/PGF2alpha) | 41 | 2.46 | ▲ |
| 436.7749 | 7.83 | HMDB0284095 | PE(22:6(5Z,8E,10Z,13Z,15E,19Z)-2OH(7S, 17S)/22:4(7Z,10Z,13Z,16Z)) | 45 | -1.25 | ▲ |
| 428.2622 | 7.83 | HMDB0013520 | PGP(18:1(11Z)/18:1(11Z)) | 43.1 | 2.87 | ▲ |
| 445.2869 | 7.83 | HMDB0290726 | SM(d20:1/6 keto-PGF1alpha) | 44.2 | 1.33 | ▲ |
| 198.1487 | 8.71 | HMDB0013279 | N-Nonanoylglycine | 49.1 | -0.79 | ▲ |
| 278.2465 | 11.33 | HMDB0242034 | N-Lauroyl Isoleucine | 43.4 | -4.42 | ▲ |
| 316.2850 | 12.07 | HMDB0038057 | Dehydrophytosphingosine | 49.5 | 1.22 | ▲ |
| 817.5827 | 12.10 | HMDB0000371 | 1,3,12-Trihydroxycholan-24-oic acid | 45.2 | 0.37 | ▼ |
| 305.1741 | 12.45 | HMDB0251955 | Estetrol | 40.2 | -2.25 | ▲ |
| 290.2095 | 12.84 | HMDB0040168 | Glycerol 1-(5-hydroxydodecanoate) | 48.3 | 2.53 | ▲ |
| 314.2699 | 12.87 | HMDB0004701 | 9-10-Epoxyoctadecenoic acid | 43.8 | 3.26 | ▲ |
| 254.2481 | 13.01 | HMDB0256086 | Palmitoleamide | 39.4 | 0.93 | ▲ |
| 308.2210 | 13.03 | HMDB0005049 | 10-Nitrolinoleic acid | 38.6 | -3.10 | ▲ |
| 373.3057 | 13.23 | HMDB0251406 | Dimethylsphingosine | 48.8 | -1.00 | ▲ |
| 336.2507 | 13.24 | HMDB0013034 | Palmitoylglycine | 45.1 | -0.80 | ▲ |
| 332.2045 | 13.41 | HMDB0003464 | 4-Guanidinobutanoic acid | 40.5 | 1.34 | ▲ |
| 306.2415 | 13.63 | HMDB0000826 | Pentadecanoic acid | 37.3 | 4.75 | ▲ |
| 397.1938 | 13.65 | HMDB0241735 | 3-Hydroxyoctanedioylcarnitine | 39.3 | -2.19 | ▲ |
| 294.2433 | 13.72 | HMDB0302694 | Hexadecadienoic acid | 51.4 | 2.08 | ▲ |
| 454.4253 | 13.84 | HMDB0062678 | N-hexacosanoylglycine | 49.1 | -0.36 | ▼ |
| 343.3203 | 13.87 | HMDB0002231 | 11Z-Eicosenoic acid | 41.2 | -1.35 | ▲ |
| 169.1225 | 13.92 | HMDB0013105 | trans-4-5-epoxy-2(E)-decenal | 41.2 | 1.27 | ▲ |
| 279.2326 | 14.09 | HMDB0001388 | alpha-Linolenic acid | 45 | 2.56 | ▲ |
| 613.4920 | 14.09 | HMDB0289865 | Cer(d16:1/18:1(12Z)-O(9S,10R)) | 51.4 | 0.88 | ▲ |
| 318.2409 | 14.09 | HMDB0010735 | Trans-Hexa-dec-2-enoic acid | 44 | 2.05 | ▲ |
| 496.3404 | 14.11 | HMDB0010382 | LysoPC(16:0/0:0) | 51.4 | 1.31 | ▼ |
| 395.1829 | 14.24 | HMDB0001497 | Nicotine-1'-N-oxide | 37.1 | -4.28 | ▲ |
| 341.3169 | 14.24 | HMDB0000252 | Sphingosine | 40.4 | 2.07 | ▲ |
| 522.3561 | 14.38 | HMDB0256159 | PC(18:1(6Z)/0:0) | 48.9 | 1.25 | ▼ |
| 298.2741 | 14.56 | HMDB0245914 | 3-Ketosphingosine | 52.4 | 0.10 | ▲ |
| 343.3314 | 14.56 | HMDB0002088 | Oleoylethanolamide | 51.7 | -1.53 | ▲ |
| 240.2324 | 14.65 | HMDB0004305 | Farnesol | 45.2 | 1.08 | ▲ |
| 594.3772 | 14.74 | HMDB0000347 | 16b-Hydroxyestradiol | 41.7 | -3.05 | ▲ |
| 766.5354 | 14.79 | HMDB0008994 | PE(18:0/18:2(9Z-12Z)) | 45.5 | -0.47 | ▲ |
| 501.3670 | 14.93 | HMDB0242379 | Cholyllysine | 40.9 | -3.21 | ▲ |
| 346.2715 | 14.94 | HMDB0012252 | Linoleoyl ethanolamide | 53.6 | -0.50 | ▲ |
| 228.2326 | 14.96 | HMDB0302568 | 9-Tetradecenal | 55.2 | 1.79 | ▲ |
| 644.4915 | 15.03 | HMDB0298848 | DG(20:5(5Z,8Z,11Z,14Z,16E)-OH(18R)/0:0/i-14:0) | 45.5 | 4.96 | ▲ |
| 294.2434 | 15.07 | HMDB0240796 | 5-Methyldecanoylcarnitine | 41.8 | 1.92 | ▲ |
| 503.3811 | 15.13 | HMDB0241563 | (9Z)-Nonadec-9-enoylcarnitine | 52.2 | -1.88 | ▲ |
| 139.1120 | 15.23 | HMDB0004362 | 4-Hydroxynonenal | 48.4 | 1.67 | ▲ |
| 417.2740 | 15.23 | HMDB0290630 | SM(d19:0/5-iso PGF2VI) | 44 | 1.92 | ▲ |
| 155.1070 | 15.26 | HMDB0029306 | 4-Ethylphenol | 45 | 2.66 | ▲ |
| 368.2207 | 15.26 | HMDB0240799 | 8-Methyldecanoylcarnitine | 37.1 | 2.81 | ▲ |
| 375.3213 | 15.26 | HMDB0241933 | N-Palmitoyl Threonine | 44.2 | -1.17 | ▲ |
| 625.8875 | 15.31 | HMDB0116138 | CDP-DG(a-25:0/a-25:0) | 42.8 | 0.46 | ▲ |

Table S10: List of identified metabolites between placebo and AC of HPI extracts in ESI^+^ mode.

| **m/z** | **RT (min)** | **HMDB ID** | **Accepted Description** | **Score** | **ppm** | **Trend** |
| --- | --- | --- | --- | --- | --- | --- |
| 371.2071 | 0.36 | HMDB0267689 | PA(i-15:0/20:5(6E,8Z,11Z,14Z,17Z)-OH(5)) | 45.7 | -1.26 | ▼ |
| 294.6657 | 0.38 | HMDB0252757 | Glu-Ile-Leu-Asp-Val | 46.6 | 0.24 | ▼ |
| 130.0867 | 0.40 | HMDB0029449 | (2R,3R,4R)-2-Amino-4-hydroxy-3-methylpentanoic acid | 56.2 | 3.31 | ▼ |
| 275.1718 | 0.40 | HMDB0013131 | 3-Hydroxyhexanoylcarnitine | 45.6 | -3.36 | ▼ |
| 84.0804 | 0.40 | HMDB0012815 | 5-Aminopentanal | 55.2 | -4.03 | ▼ |
| 303.1774 | 0.40 | HMDB0028723 | Arginyl-Gamma-glutamate | 43.8 | -0.35 | ▼ |
| 218.1502 | 0.40 | HMDB0028944 | Lysylalanine | 49.4 | 1.10 | ▼ |
| 301.0768 | 0.41 | HMDB0011648 | 1-(beta-D-Ribofuranosyl)-1,4-dihydronicotinamide | 44.5 | -0.95 | ▼ |
| 306.1581 | 0.45 | HMDB0028967 | Methionyl-Arginine | 52.3 | -4.27 | ▼ |
| 133.0606 | 0.47 | HMDB0000168 | L-Asparagine | 40.4 | -1.13 | ▼ |
| 240.0964 | 0.47 | HMDB0029032 | Serylalanine | 48.3 | 4.99 | ▼ |
| 116.0705 | 0.60 | HMDB0000162 | L-Proline | 43.7 | -0.89 | ▼ |
| 290.1445 | 0.61 | HMDB0000279 | Saccharopine | 40.9 | 3.05 | ▼ |
| 358.8656 | 0.65 | HMDB0294110 | CDP-DG(i-20:0/20:4(5E,8Z,12Z,14Z)-OH(11R)) | 47.5 | 2.82 | ▼ |
| 238.1251 | 0.72 | HMDB0114778 | PA(10:0/8:0) | 39.4 | -0.62 | ▼ |
| 194.1098 | 0.73 | HMDB0040901 | 13-Hydroxy-9-methoxy-10-oxo-11-octadecenoic acid | 46.1 | 1.65 | ▼ |
| 251.1090 | 0.73 | HMDB0010315 | 4-Hydroxyandrostenedione glucuronide | 42.8 | 2.43 | ▼ |
| 346.1695 | 0.73 | HMDB0292868 | CDP-DG(PGE2/a-15:0) | 43.4 | 3.29 | ▼ |
| 519.2263 | 0.73 | HMDB0029133 | Valylmethionine | 40.2 | -3.70 | ▼ |
| 221.1698 | 0.75 | HMDB0006764 | 17a,20a-Dihydroxycholesterol | 42.4 | -3.69 | ▼ |
| 159.0766 | 0.75 | HMDB0028851 | Glycyl-Threonine | 46.4 | 1.27 | ▼ |
| 213.1600 | 0.75 | HMDB0029130 | Valylisoleucine | 48.7 | 0.89 | ▲ |
| 243.1343 | 1.06 | HMDB0028823 | Glutamylleucine | 53.2 | 1.58 | ▲ |
| 197.1287 | 1.06 | HMDB0028917 | Isoleucyl-Threonine | 53.1 | 1.15 | ▲ |
| 253.1185 | 1.19 | HMDB0029004 | Phenylalanylserine | 48.8 | 0.84 | ▲ |
| 231.1707 | 1.50 | HMDB0028920 | Isoleucyl-Valine | 48.7 | 1.64 | ▲ |
| 301.1943 | 1.80 | HMDB0115692 | PA(8:0/i-19:0) | 43 | -4.66 | ▲ |
| 253.1185 | 2.19 | HMDB0029098 | Tyrosyl-Alanine | 49.8 | 0.99 | ▲ |
| 247.1292 | 2.40 | HMDB0028756 | Aspartyl-Isoleucine | 49.4 | 1.27 | ▲ |
| 406.2612 | 2.75 | HMDB0269965 | PG(18:1(9Z)-O(12,13)/20:4(5Z,8Z,11Z,14Z)) | 50.6 | 3.84 | ▼ |
| 217.1549 | 2.81 | HMDB0094801 | 2-octenoylglycine | 44.5 | 1.27 | ▲ |
| 267.1344 | 2.81 | HMDB0029068 | Threonylphenylalanine | 50.4 | 1.73 | ▲ |
| 231.1708 | 2.91 | HMDB0029131 | Valylleucine | 51.6 | 1.88 | ▲ |
| 277.1195 | 3.26 | HMDB0028994 | Phenylalanylglutamic acid | 53.1 | 3.97 | ▲ |
| 354.1662 | 3.40 | HMDB0241751 | 6-Hydroxynon-7-enoylcarnitine | 50.9 | -4.92 | ▲ |
| 527.3553 | 3.45 | HMDB0297154 | DG(5-iso PGF2VI/0:0/8:0) | 47.5 | -4.84 | ▲ |
| 217.1548 | 3.65 | HMDB0029140 | Valylvaline | 50 | 0.44 | ▲ |
| 201.1235 | 3.67 | HMDB0028938 | Leucyl-Serine | 50.7 | 0.47 | ▲ |
| 281.1131 | 3.72 | HMDB0003331 | 1-Methyladenosine | 45.6 | 4.50 | ▲ |
| 295.1653 | 3.76 | HMDB0011177 | Phenylalanylproline | 48.5 | 0.30 | ▲ |
| 500.3560 | 3.79 | HMDB0241874 | 7-[(1R,2R,3R)-3-Hydroxy-2-[(3S)-3-hydroxyoctyl]-5-oxocyclopentyl]heptanoylcarnitine | 41.7 | -4.36 | ▲ |
| 263.1033 | 3.79 | HMDB0000706 | Aspartylphenylalanine | 44.2 | 2.46 | ▲ |
| 420.7220 | 3.89 | HMDB0282000 | PS(18:1(9Z)-O(12,13)/18:4(6Z,9Z,12Z,15Z)) | 49 | -3.80 | ▲ |
| 423.2226 | 3.96 | HMDB0266102 | PA(PGJ2/22:5(7Z,10Z,13Z,16Z,19Z)) | 41.2 | 4.87 | ▲ |
| 514.2808 | 4.00 | HMDB0279526 | PIP(20:0/20:4(8Z,11Z,14Z,17Z)-2OH(5S,6R)) | 44.5 | 2.43 | ▲ |
| 343.2180 | 4.04 | HMDB0294878 | DG(6 keto-PGF1alpha/13:0/0:0) | 41.9 | 3.96 | ▲ |
| 412.7127 | 4.04 | HMDB0263093 | PA(LTE4/14:1(9Z)) | 49.7 | 4.68 | ▲ |
| 201.1235 | 4.09 | HMDB0033675 | L-Lysopine | 51.1 | 0.64 | ▲ |
| 362.7308 | 4.16 | HMDB0260661 | PE(18:3(9,11,15)-OH(13)/14:0) | 48.3 | 2.69 | ▲ |
| 420.7223 | 4.18 | HMDB0280882 | PS(22:6(4Z,7Z,10Z,13E,15E,19Z)-OH(17)/14:0) | 41.5 | -3.21 | ▲ |
| 373.2445 | 4.20 | HMDB0242036 | N-Lauroyl Lysine | 53.5 | 2.40 | ▼ |
| 539.2539 | 4.30 | HMDB0280246 | PIP(20:5(6E,8Z,11Z,14Z,17Z)-OH(5)/22:2(13Z,16Z)) | 38.4 | -4.47 | ▲ |
| 539.2719 | 4.49 | HMDB0280353 | PIP(22:3(10Z,13Z,16Z)/20:5(7Z,9Z,11E,13E,17Z)-3OH(5,6,15)) | 39.6 | 4.96 | ▲ |
| 302.1975 | 4.57 | HMDB0294457 | DG(10:0/PGD2/0:0) | 42.6 | 1.75 | ▼ |
| 345.2233 | 4.57 | HMDB0298864 | DG(22:6(5Z,8E,10Z,13Z,15E,19Z)-2OH(7S, 17S)/0:0/i-14:0) | 43.1 | 4.64 | ▲ |
| 407.7357 | 4.57 | HMDB0112656 | PS(20:4(8Z,11Z,14Z,17Z)/15:0) | 47.8 | 4.59 | ▼ |
| 496.8145 | 4.57 | HMDB0283700 | PS(PGF1alpha/24:0) | 40.7 | 4.45 | ▲ |
| 432.7402 | 4.65 | HMDB0270565 | PG(LTE4/a-13:0) | 43.3 | 4.52 | ▼ |
| 419.7744 | 4.67 | HMDB0009639 | PE(22:5(4Z,7Z,10Z,13Z,16Z)/22:6(4Z,7Z,10Z,13Z,16Z,19Z)) | 41.5 | 4.08 | ▼ |
| 280.1909 | 4.67 | HMDB0116762 | PS(20:1(11Z)/20:4(8Z,11Z,14Z,17Z)) | 44.4 | -1.32 | ▼ |
| 798.4395 | 4.69 | HMDB0000418 | 18-Hydroxycortisol | 41.4 | -3.66 | ▲ |
| 671.3703 | 4.69 | HMDB0006254 | 4-Hydroxyretinoic acid | 47.9 | -0.80 | ▲ |
| 823.9599 | 4.69 | HMDB0004858 | Ganglioside GM1 (18:1/22:0) | 48 | 1.26 | ▲ |
| 345.2231 | 4.72 | HMDB0298893 | DG(i-14:0/22:6(4Z,7Z,11E,13Z,15E,19Z)-2OH(10S,17)/0:0) | 45.6 | 3.90 | ▲ |
| 263.1389 | 4.76 | HMDB0029118 | Tyrosyl-Valine | 49.4 | -0.31 | ▲ |
| 332.2147 | 4.84 | HMDB0295095 | DG(14:0/0:0/PGJ2) | 41.8 | 2.22 | ▼ |
| 171.1131 | 4.89 | HMDB0033926 | (3xi,6xi)-Cyclo(alanylvalyl) | 51.4 | 1.54 | ▲ |
| 336.3794 | 4.89 | HMDB0243947 | 1-Methylspermidine | 37.9 | -4.85 | ▲ |
| 143.1178 | 4.89 | HMDB0006009 | Isoputreanine | 46.6 | -0.44 | ▲ |
| 571.8086 | 4.91 | HMDB0294382 | CDP-DG(22:6(4Z,7Z,11E,13Z,15E,19Z)-2OH(10S,17)/i-22:0) | 46.9 | 4.12 | ▼ |
| 714.5654 | 5.00 | HMDB0240638 | SM(d18:2(4E,14Z)/16:0) | 36.8 | 2.78 | ▼ |
| 524.2692 | 5.15 | HMDB0266539 | PA(2:0/18:1(12Z)-2OH(9,10)) | 37.9 | -3.35 | ▲ |
| 367.7323 | 5.22 | HMDB0260614 | PE(14:0/5-iso PGF2VI) | 49.1 | -4.08 | ▼ |
| 185.1285 | 5.23 | HMDB0028690 | Alanylisoleucine | 51.1 | 0.35 | ▲ |
| 835.5033 | 5.23 | HMDB0113490 | PE-NMe(20:5(5Z,8Z,11Z,14Z,17Z)/18:4(6Z,9Z,12Z,15Z)) | 40.5 | 4.67 | ▲ |
| 762.5969 | 5.27 | HMDB0296698 | DG(PGE2/0:0/22:0) | 37.4 | 0.05 | ▼ |
| 201.1235 | 5.30 | HMDB0242765 | (2R)-2-Amino-2-[[(1S)-1-carboxyethyl]amino]-4-methylpentanoic acid | 49.1 | 0.55 | ▲ |
| 475.2765 | 5.30 | HMDB0277958 | PI(22:4(10Z,13Z,16Z,19Z)/20:5(5Z,8Z,11Z,14Z,16E)-OH(18R)) | 48.6 | 2.16 | ▲ |
| 421.2521 | 5.35 | HMDB0116600 | PG(22:6(4Z,7Z,10Z,13Z,16Z,19Z)/18:2(9Z,12Z)) | 50.3 | -2.49 | ▲ |
| 367.7322 | 5.37 | HMDB0260615 | PE(5-iso PGF2VI/14:0) | 47.4 | -4.30 | ▲ |
| 413.2714 | 5.40 | HMDB0290367 | SM(d17:1/TXB2) | 42 | -1.24 | ▲ |
| 1012.4978 | 5.42 | HMDB0274188 | PGP(22:6(4Z,7Z,10Z,13Z,16Z,19Z)/PGD2) | 51.9 | 3.26 | ▲ |
| 729.5628 | 5.47 | HMDB0011207 | PC(P-16:0/16:1(9Z)) | 36.4 | -0.51 | ▼ |
| 439.2043 | 5.52 | HMDB0247924 | Acetyl-arginyl-glycyl-aspartyl-serinamide | 44.4 | -0.98 | ▲ |
| 801.3658 | 5.52 | HMDB0248578 | Arg-Pro-Pro-Gly-Phe-Ser-Pro | 42.1 | 3.67 | ▲ |
| 585.7929 | 5.55 | HMDB0294380 | CDP-DG(22:6(4Z,7Z,10Z,13E,15E,19Z)-OH(17)/i-22:0) | 47.7 | 3.74 | ▲ |
| 480.2722 | 5.67 | HMDB0275720 | PGP(i-21:0/20:3(8Z,11Z,14Z)-O(5,6)) | 44.9 | -1.56 | ▲ |
| 741.4853 | 5.69 | HMDB0290062 | Cer(d18:2(4E,14Z)/LTE4) | 48.5 | 0.88 | ▲ |
| 543.2801 | 5.69 | HMDB0280208 | PIP(6 keto-PGF1alpha/22:2(13Z,16Z)) | 45.1 | -4.11 | ▲ |
| 963.5604 | 5.74 | HMDB0277819 | PI(22:3(10Z,13Z,16Z)/PGE2) | 53.8 | 1.05 | ▲ |
| 652.3989 | 5.88 | HMDB0241733 | Octanedioylcarnitine | 41.2 | -4.03 | ▲ |
| 350.2070 | 5.93 | HMDB0303977 | (5Z)-(15S)-11-alpha-hydroxy-9,15-dioxoprosta-13-enoate | 47.9 | -4.99 | ▲ |
| 644.3299 | 5.98 | HMDB0266658 | PA(20:5(7Z,9Z,11E,13E,17Z)-3OH(5,6,15)/8:0) | 44.7 | 2.21 | ▲ |
| 419.2651 | 6.20 | HMDB0271678 | PG(i-18:0/20:4(6E,8Z,11Z,14Z)-OH(5S)) | 41.2 | -2.71 | ▲ |
| 451.2256 | 6.22 | HMDB0248576 | Arg-His-Phe-Trp-Gln-Gln | 45.8 | 1.39 | ▲ |
| 902.4387 | 6.22 | HMDB0274814 | PGP(6 keto-PGF1alpha/i-12:0) | 49.7 | -4.61 | ▲ |
| 594.3667 | 6.32 | HMDB0242374 | Cholyltryptophan | 46 | 0.63 | ▼ |
| 861.4339 | 6.32 | HMDB0269315 | PG(18:3(6Z,9Z,12Z)/PGJ2) | 43.7 | 3.01 | ▲ |
| 545.2657 | 6.32 | HMDB0279483 | PIP(PGF2alpha/20:0) | 55.8 | -2.03 | ▲ |
| 525.2722 | 6.42 | HMDB0280212 | PIP(PGJ2/22:2(13Z,16Z)) | 42.9 | 0.87 | ▲ |
| 528.2821 | 6.49 | HMDB0275950 | PGP(i-24:0/PGE2) | 52 | -0.32 | ▼ |
| 510.7921 | 6.59 | HMDB0116059 | CDP-DG(18:2(9Z,11Z)/i-19:0) | 39.5 | 8.20 | ▲ |
| 976.4916 | 6.64 | HMDB0275330 | PGP(i-17:0/PGD2) | 43.1 | -0.76 | ▲ |
| 890.8132 | 6.73 | HMDB0012122 | (Mannosyl)6-(N-acetylglucosaminyl)2-diphosphodolichol | 46.7 | -0.13 | ▼ |
| 445.3018 | 6.81 | HMDB0241510 | 5-Hydroxyhexadecanedioylcarnitine | 44.9 | -3.49 | ▲ |
| 1101.5487 | 6.81 | HMDB0293915 | CDP-DG(PGJ2/i-18:0) | 48.7 | -4.59 | ▲ |
| 813.6215 | 6.88 | HMDB0009008 | PE(18:0/22:2(13Z,16Z)) | 36.1 | 1.10 | ▲ |
| 566.2370 | 6.91 | HMDB0293500 | CDP-DG(i-14:0/LTE4) | 42 | -4.15 | ▲ |
| 660.5543 | 6.91 | HMDB0093016 | DG(10:0/0:0/i-24:0) | 38 | 0.93 | ▲ |
| 951.9516 | 6.93 | HMDB0252614 | gamma-Endorphin | 42.9 | 3.48 | ▲ |
| 1011.2803 | 7.06 | HMDB0003712 | (2E)-Dodecenoyl-CoA | 37 | -2.18 | ▼ |
| 608.8264 | 7.08 | HMDB0293184 | CDP-DG(6 keto-PGF1alpha/a-25:0) | 41.9 | 4.96 | ▲ |
| 475.7889 | 7.25 | HMDB0284896 | PE(DiMe(13,5)/TXB2) | 43.6 | -2.50 | ▲ |
| 910.1560 | 7.48 | HMDB0001564 | CDP-ethanolamine | 38.1 | 1.57 | ▼ |
| 1120.5562 | 7.49 | HMDB0294170 | CDP-DG(22:6(4Z,7Z,10Z,12E,16Z,19Z)-OH(14)/i-20:0) | 53.5 | -4.43 | ▲ |
| 1062.5094 | 7.68 | HMDB0290900 | CDP-DG(PGJ2/16:1(9Z)) | 47.7 | 2.96 | ▲ |
| 521.7419 | 7.81 | HMDB0292897 | CDP-DG(a-15:0/18:1(12Z)-2OH(9,10)) | 37.5 | 1.21 | ▼ |
| 517.9854 | 7.81 | HMDB0001541 | Guanosine hexaphosphate adenosine | 37.1 | -1.45 | ▼ |
| 508.7644 | 7.81 | HMDB0276894 | PI(LTE4/18:3(9Z,12Z,15Z)) | 42.9 | 4.91 | ▼ |
| 718.6449 | 7.85 | HMDB0007658 | DG(22:2(13Z,16Z)/20:0/0:0) | 38.7 | 2.14 | ▼ |
| 813.4411 | 7.85 | HMDB0115168 | PA(20:4(5Z,8Z,11Z,14Z)/22:6(4Z,7Z,10Z,13Z,16Z,19Z)) | 37.1 | -3.95 | ▼ |
| 766.5408 | 7.95 | HMDB0285575 | PE(PGF1alpha/P-18:1(9Z)) | 44.6 | 3.39 | ▼ |
| 791.5916 | 8.06 | HMDB0290583 | SM(d18:2(4E,14Z)/18:1(12Z)-2OH(9,10)) | 48.4 | 0.88 | ▼ |
| 697.1868 | 8.18 | HMDB0010329 | Dopamine glucuronide | 37.2 | 2.36 | ▼ |
| 871.7332 | 8.18 | HMDB0008785 | PC(24:0/P-18:0) | 36.5 | -2.41 | ▼ |
| 928.4842 | 8.25 | HMDB0274110 | PGP(22:5(7Z,10Z,13Z,16Z,19Z)/18:2(9Z,11E)+=O(13)) | 44 | 1.73 | ▲ |
| 917.5064 | 8.36 | HMDB0268694 | PG(LTE4/16:1(9Z)) | 42.9 | 1.85 | ▼ |
| 966.2809 | 8.43 | HMDB0300956 | Undec-3-enoyl-CoA | 38.2 | -3.84 | ▼ |
| 689.3436 | 8.44 | HMDB0240734 | Taurodeoxycholic acid 3-glucuronide | 38.9 | 4.66 | ▼ |
| 804.7548 | 8.51 | HMDB0240648 | Stearoylcholine | 36.7 | 2.67 | ▼ |
| 1097.5726 | 8.56 | HMDB0241822 | (5Z)-7-[(1R,2R,3R)-3-Hydroxy-2-[(1E,3S)-3-hydroxy-5-phenylpent-1-en-1-yl]-5-oxocyclopentyl]hept-5-enoylcarnitine | 45.3 | 1.41 | ▲ |
| 754.5905 | 8.85 | HMDB0242027 | N-Lauroyl Arginine | 35.2 | -1.10 | ▲ |
| 656.5081 | 8.87 | HMDB0299230 | DG(i-16:0/0:0/5-iso PGF2VI) | 36.3 | -2.40 | ▼ |
| 881.6840 | 8.87 | HMDB0008158 | PC(18:2(9Z,12Z)/24:1(15Z)) | 38.7 | 0.83 | ▼ |
| 705.3512 | 8.87 | HMDB0288886 | PC(2:0/22:6(4Z,7Z,11E,13Z,15E,19Z)-2OH(10S,17)) | 45.2 | 4.01 | ▼ |
| 821.4384 | 8.87 | HMDB0272485 | PGP(18:3(10,12,15)-OH(9)/16:1(9Z)) | 46.6 | 2.38 | ▼ |
| 246.2430 | 9.02 | HMDB0000806 | Myristic acid | 41.7 | 1.26 | ▼ |
| 983.5114 | 9.05 | HMDB0293524 | CDP-DG(i-14:0/18:1(9Z)-O(12,13)) | 41.7 | -0.31 | ▼ |
| 840.4218 | 9.05 | HMDB0112503 | PS(18:4(6Z,9Z,12Z,15Z)/20:5(5Z,8Z,11Z,14Z,17Z)) | 40.9 | 0.75 | ▲ |
| 1044.5269 | 9.12 | HMDB0280499 | PIP(22:4(7Z,10Z,13Z,16Z)/20:4(5Z,8Z,11Z,14Z)-OH(19S)) | 45.7 | -2.99 | ▲ |
| 918.4550 | 9.44 | HMDB0272465 | PGP(22:6(5Z,8E,10Z,13Z,15E,19Z)-2OH(7S, 17S)/16:1(9Z)) | 37.4 | -7.62 | ▼ |
| 207.1749 | 9.51 | HMDB0030982 | 6-Ketomyristic acid | 49.5 | 2.37 | ▼ |
| 1100.7856 | 9.71 | HMDB0004886 | Trihexosylceramide (d18:1/24:0) | 41.8 | 3.21 | ▼ |
| 1036.5213 | 9.73 | HMDB0279589 | PIP(PGJ2/20:1(11Z)) | 41.5 | -3.46 | ▲ |
| 620.2136 | 10.10 | HMDB0002091 | Isovalerylglucuronide | 37.9 | -4.51 | ▼ |
| 899.4883 | 10.12 | HMDB0282841 | PS(20:5(5Z,8Z,11Z,14Z,17Z)/22:6(5Z,8E,10Z,13Z,15E,19Z)-2OH(7S, 17S)) | 38.6 | -2.78 | ▼ |
| 962.0133 | 10.38 | HMDB0001380 | Diguanosine pentaphosphate | 38 | -2.86 | ▼ |
| 945.5081 | 10.48 | HMDB0002586 | Chenodeoxycholic acid 3-sulfate | 41.6 | 2.03 | ▼ |
| 939.0881 | 10.51 | HMDB0059613 | 7-Methylguanosine 5'-diphosphate | 36.4 | 3.55 | ▼ |
| 987.5118 | 10.53 | HMDB0275882 | PGP(i-22:0/20:5(5Z,8Z,11Z,14Z,16E)-OH(18R)) | 41 | -0.71 | ▼ |
| 261.1104 | 10.77 | HMDB0029083 | Tryptophyl-Glycine | 46.8 | -1.62 | ▼ |
| 318.2805 | 10.94 | HMDB0241931 | N-Palmitoyl Proline | 47.5 | 3.77 | ▼ |
| 290.2695 | 11.05 | HMDB0006294 | 16-Hydroxyhexadecanoic acid | 56.1 | 1.87 | ▼ |
| 272.2590 | 11.09 | HMDB0242181 | Hexadecasphingosine | 45.2 | 2.08 | ▼ |
| 272.2589 | 11.33 | HMDB0037647 | (E)-11-Hexadecenoic acid | 45.2 | 1.98 | ▼ |
| 302.3058 | 11.85 | HMDB0000269 | Sphinganine | 43.1 | 1.42 | ▼ |
| 318.3000 | 11.96 | HMDB0304183 | 4-hydroxysphinganine | 56.9 | -0.93 | ▼ |
| 870.4965 | 12.10 | HMDB0264547 | PA(LTE4/19:2(10Z,13Z)) | 36.8 | 1.79 | ▲ |
| 799.5718 | 12.10 | HMDB0290754 | SM(d20:1/22:6(4Z,7Z,11E,13Z,15E,19Z)-2OH(10S,17)) | 51.2 | -3.61 | ▲ |
| 367.3322 | 12.46 | HMDB0241929 | N-Palmitoyl Lysine | 51 | 0.74 | ▼ |
| 330.3370 | 12.67 | HMDB0304489 | sphinganine (C20) | 45.5 | 1.05 | ▼ |
| 288.2536 | 12.97 | HMDB0030972 | 8-Oxohexadecanoic acid | 51.1 | 0.99 | ▼ |
| 478.2982 | 13.67 | HMDB0242415 | Deoxycholylcysteine | 43.1 | -0.78 | ▼ |
| 546.3564 | 14.07 | HMDB0010394 | LysoPC(20:3(8Z,11Z,14Z)/0:0) | 50.9 | 1.85 | ▼ |
| 267.6475 | 14.11 | HMDB0242372 | Cholylcysteine | 52.7 | 3.42 | ▼ |
| 442.2589 | 14.35 | HMDB0241355 | 3,10-Dihydroxytetradecanoylcarnitine | 42.3 | 5.85 | ▼ |
| 478.2358 | 14.36 | HMDB0242076 | N-Eicosapentaenoyl Methionine | 42.6 | -0.96 | ▼ |
| 262.1443 | 14.36 | HMDB0259061 | Thyronamine | 50.1 | 2.32 | ▼ |
| 140.0686 | 14.38 | HMDB0246748 | 5-Aminoimidazole-4-carboxyamide | 42.7 | -4.88 | ▼ |
| 508.3754 | 14.60 | HMDB0013122 | LysoPC(P-18:0/0:0) | 41.3 | -1.49 | ▼ |
| 301.1416 | 14.68 | HMDB0001518 | alpha-CEHC | 45.7 | 2.22 | ▼ |
| 285.1694 | 14.79 | HMDB0059661 | QH(2) | 46 | -1.08 | ▼ |
| 309.2040 | 15.23 | HMDB0041287 | 16-Hydroxy-10-oxohexadecanoic acid | 50.2 | 1.40 | ▼ |
| 448.2909 | 15.31 | HMDB0241373 | (5Z)-3-Hydroxytetradec-5-enedioylcarnitine | 40.6 | 0.95 | ▼ |
| 185.0812 | 15.31 | HMDB0000895 | Acetylcholine | 41.8 | -0.56 | ▼ |
| 422.2066 | 15.31 | HMDB0274812 | PGP(PGD2/i-12:0) | 41.2 | 0.47 | ▼ |
| 369.3520 | 15.49 | HMDB0245858 | (5Z,7E)-9,10-Seco-5,7,10(19)-cholestatriene | 44.9 | 1.26 | ▼ |
| 413.3243 | 16.37 | HMDB0011545 | MG(0:0/20:3(11Z,14Z,17Z)/0:0) | 46 | -4.80 | ▼ |

Table S11: Metabolic pathways of placebo in comparison with MOE of HPO extracts in ESI^+^ mode.

| Pathway name | Pathway source | Overlapping metabolites | All metabolites | Pmetabolites |
| --- | --- | --- | --- | --- |
| Cholesterol metabolism | Wikipathways | 2 | 4 | 0.001 |
| Arginine and proline metabolism | KEGG | 4 | 60 | 0.002 |
| γ-glutamyl cycle | HumanCyc | 3 | 29 | 0.002 |
| leukotriene biosynthesis | HumanCyc | 3 | 29 | 0.002 |
| Plasma lipoprotein remodeling | Reactome | 2 | 10 | 0.004 |
| HDL remodeling | Reactome | 2 | 10 | 0.004 |
| Steroid biosynthesis | KEGG | 3 | 40 | 0.006 |
| Cytochrome P450 - arranged by substrate type | Reactome | 4 | 87 | 0.009 |
| Phase I - Functionalization of compounds | Reactome | 5 | 143 | 0.010 |
| Amino Acid metabolism | Wikipathways | 4 | 92 | 0.010 |
| Urea cycle and metabolism of arginine. proline. glutamate. aspartate and asparagine | EHMN | 4 | 93 | 0.011 |
| Membrane Trafficking | Reactome | 2 | 20 | 0.015 |
| Arginine Proline metabolism | INOH | 3 | 58 | 0.017 |
| Cytosolic and mitochondrial tRNA aminoacylation | Reactome | 2 | 22 | 0.018 |
| tRNA Aminoacylation | Reactome | 2 | 22 | 0.018 |
| Biological oxidations | Reactome | 6 | 231 | 0.019 |
| Aminoacyl-tRNA biosynthesis | KEGG | 2 | 23 | 0.020 |
| Ovarian steroidogenesis | KEGG | 2 | 23 | 0.020 |
| Transport of small molecules | Reactome | 5 | 172 | 0.021 |
| tRNA charging | HumanCyc | 2 | 24 | 0.021 |
| Mineral absorption | KEGG | 2 | 27 | 0.027 |
| Na+/Cl- dependent neurotransmitter transporters | Reactome | 2 | 28 | 0.029 |
| Translation | Reactome | 2 | 29 | 0.031 |
| Amino acid transport across the plasma membrane | Reactome | 2 | 29 | 0.031 |
| Endogenous sterols | Reactome | 2 | 31 | 0.035 |
| Vesicle-mediated transport | Reactome | 2 | 31 | 0.035 |
| G alpha (i) signalling events | Reactome | 3 | 77 | 0.036 |
| S-methyl-5-thio-α-D-ribose 1-phosphate degradation | HumanCyc | 2 | 34 | 0.041 |

Table S12: Metabolic pathways of placebo in comparison with RA of HPO extracts in ESI^+^ mode.

| Pathway name | Pathway source | Overlapping metabolites | All metabolites | Pmetabolites |
| --- | --- | --- | --- | --- |
| Phosphatidylcholine catabolism | Wikipathways | 2 | 6 | 0.0002 |
| Phospholipid Biosynthesis | SMPDB | 2 | 27 | 0.0036 |
| Glycosphingolipid metabolism | Reactome | 2 | 31 | 0.0047 |
| Sphingolipid metabolism | Reactome | 2 | 54 | 0.0138 |

| Pathway name | Pathway source | Overlapping metabolites | All metabolites | Pmetabolites |
| --- | --- | --- | --- | --- |
| Sphingolipid de novo biosynthesis | Reactome | 3 | 31 | 0.00001 |
| Sphingolipid metabolism | Reactome | 3 | 54 | 0.00008 |
| Sphingolipid pathway | Wikipathways | 2 | 13 | 0.00023 |
| Glycosphingolipid metabolism | EHMN | 2 | 40 | 0.00222 |

Table S13: Metabolic pathways of placebo in comparison with AC of HPO extracts in ESI+ mode.

Table S14: Metabolic pathways of placebo compared to MOE of HPI extracts in ESI^+^ mode.

| Pathway name | Pathway source | Overlapping metabolites | All metabolites | Pmetabolites |
| --- | --- | --- | --- | --- |
| Glycosphingolipid metabolism | Reactome | 2 | 31 | 0.0070 |
| Sphingolipid metabolism | Reactome | 2 | 54 | 0.0205 |
| Steroid hormone biosynthesis | KEGG | 2 | 87 | 0.0497 |

Table S15: Metabolic pathways of placebo compared to RA of HPI extracts in ESI^+^ mode.

| Pathway name | Pathway source | Overlapping metabolites | All metabolites | Pmetabolites |
| --- | --- | --- | --- | --- |
| Linoleate metabolism | EHMN | 2 | 21 | 0.0033 |
| Free fatty acid receptors | Reactome | 2 | 23 | 0.0039 |
| Signal Transduction | Reactome | 4 | 206 | 0.0080 |
| sphingosine and sphingosine-1-phosphate metabolism | HumanCyc | 2 | 37 | 0.0099 |
| Biosynthesis of unsaturated fatty acids | KEGG | 2 | 39 | 0.0110 |
| GPCR downstream signalling | Reactome | 3 | 118 | 0.0114 |
| Signaling by GPCR | Reactome | 3 | 142 | 0.0188 |
| G alpha (q) signalling events | Reactome | 2 | 56 | 0.0220 |

Table S16: Metabolic pathways of placebo compared to AC of HPI extracts in ESI^+^ mode.

| Pathway name | Pathway source | Overlapping metabolites | All metabolites | Pmetabolites |
| --- | --- | --- | --- | --- |
| Lysine degradation | KEGG | 4 | 40 | 0.0002 |
| Glycerophospholipid metabolism | KEGG | 3 | 25 | 0.0008 |
| α-tocopherol degradation | HumanCyc | 2 | 8 | 0.0015 |
| Metabolism of Spingolipids in ER and Golgi apparatus | Wikipathways | 2 | 10 | 0.0024 |
| Glycerophospholipid biosynthesis | Reactome | 3 | 47 | 0.0050 |
| Phospholipid metabolism | Reactome | 3 | 50 | 0.0060 |
| Arginine and proline metabolism | KEGG | 3 | 60 | 0.0100 |
| Glycerophospholipid metabolism | EHMN | 3 | 62 | 0.0109 |
| Cytosolic tRNA aminoacylation | Reactome | 2 | 22 | 0.0118 |
| Mitochondrial tRNA aminoacylation | Reactome | 2 | 22 | 0.0118 |
| tRNA Aminoacylation | Reactome | 2 | 22 | 0.0118 |
| Aminoacyl-tRNA biosynthesis | KEGG | 2 | 23 | 0.0128 |
| tRNA charging | HumanCyc | 2 | 24 | 0.0139 |
| Mineral absorption | KEGG | 2 | 27 | 0.0175 |
| Phospholipid Biosynthesis | SMPDB | 2 | 27 | 0.0175 |
| Na+/Cl- dependent neurotransmitter transporters | Reactome | 2 | 28 | 0.0187 |
| Translation | Reactome | 2 | 29 | 0.0200 |
| γ-glutamyl cycle | HumanCyc | 2 | 29 | 0.0200 |
| Amino acid transport across the plasma membrane | Reactome | 2 | 29 | 0.0200 |
| leukotriene biosynthesis | HumanCyc | 2 | 29 | 0.0200 |
| S-methyl-5-thio-α-D-ribose 1-phosphate degradation | HumanCyc | 2 | 34 | 0.0270 |
| Neurotransmitter release cycle | Reactome | 2 | 41 | 0.0383 |
| Transport of small molecules | Reactome | 4 | 172 | 0.0384 |
| Lysine metabolism | EHMN | 2 | 42 | 0.0400 |
| Metabolism of lipids | Reactome | 6 | 352 | 0.0426 |
| Protein digestion and absorption | KEGG | 2 | 44 | 0.0435 |
| Transport of inorganic cations/anions and amino acids/oligopeptides | Reactome | 2 | 46 | 0.0472 |
